# Aspartate/asparagine-β-hydroxylase crystal structures reveal an unexpected epidermal growth factor-like domain substrate disulfide pattern

**DOI:** 10.1038/s41467-019-12711-7

**Published:** 2019-10-28

**Authors:** Inga Pfeffer, Lennart Brewitz, Tobias Krojer, Sacha A. Jensen, Grazyna T. Kochan, Nadia J. Kershaw, Kirsty S. Hewitson, Luke A. McNeill, Holger Kramer, Martin Münzel, Richard J. Hopkinson, Udo Oppermann, Penny A. Handford, Michael A. McDonough, Christopher J. Schofield

**Affiliations:** 1 Chemistry Research Laboratory, University of Oxford, Mansfield Road, Oxford, OX1 3TA UK; 2 Structural Genomics Consortium, University of Oxford, Old Road Campus, Roosevelt Drive, Headington, OX3 7DQ UK; 3 Department of Biochemistry, University of Oxford, South Parks Road, Oxford, OX1 3QU UK; 4 Department of Physiology, Anatomy and Genetics, University of Oxford, South Parks Road, Oxford, OX1 3QX UK; 5 NDORMS, Botnar Research Centre, University of Oxford, Old Road, Oxford, OX3 7LD UK

**Keywords:** Biochemistry, Chemical biology, Structural biology

## Abstract

AspH is an endoplasmic reticulum (ER) membrane-anchored 2-oxoglutarate oxygenase whose C-terminal oxygenase and tetratricopeptide repeat (TPR) domains present in the ER lumen. AspH catalyses hydroxylation of asparaginyl- and aspartyl-residues in epidermal growth factor-like domains (EGFDs). Here we report crystal structures of human AspH, with and without substrate, that reveal substantial conformational changes of the oxygenase and TPR domains during substrate binding. Fe(II)-binding by AspH is unusual, employing only two Fe(II)-binding ligands (His679/His725). Most EGFD structures adopt an established fold with a conserved Cys1–3, 2–4, 5–6 disulfide bonding pattern; an unexpected Cys3–4 disulfide bonding pattern is observed in AspH-EGFD substrate complexes, the catalytic relevance of which is supported by studies involving stable cyclic peptide substrate analogues and by effects of Ca(II) ions on activity. The results have implications for EGFD disulfide pattern processing in the ER and will enable medicinal chemistry efforts targeting human 2OG oxygenases.

## Abstract (web summary)

AspH catalyses hydroxylation of asparagine and aspartate residues in epidermal growth factor-like domains (EGFDs). Here, the authors present crystal structures of AspH with and without substrates and show that AspH uses EFGD substrates with a non-canonical disulfide pattern.

## Introduction

**Fig. 1:**
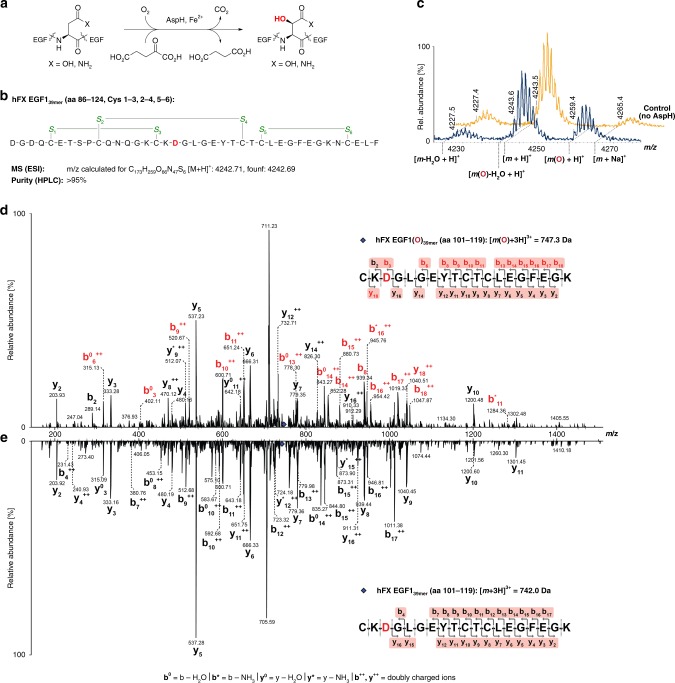
EGF1 of human coagulation factor X undergoes AspH-catalysed hydroxylation at Asp103_hFX_. MS/MS-analysis was performed using a Bruker Daltonics amaZon^TM^ Ion Trap LC-MS/MS system equipped with a Dionex^TM^ UltiMate^®^ 3000 HPLC machine. Endpoint turnover assays were performed under standard (non-redox) conditions (for details see Methods section). For MS/MS-analysis, after quenching, cystine disulfides were reduced (dithiothreitol) and cysteine thiols derivatized with iodoacetamide. **a** Reaction scheme for diastereospecific AspH-catalysed hydroxylation of Asp/Asn-residues in EGFDs. **b** Schematic structure and calculated mass of the expected disulfide isomer of hFX EGF1_39mer_ (aa 86–124) featuring a canonical disulfide connectivity pattern (Cys1–3, 2–4, 5–6; green); the hydroxylation site (Asp103_hFX_) is in red. **c** His_6_-AspH_315–758_ catalysed hydroxylation of hFX EGF1_39mer_ proceeds with ~40% conversion under standard (non-redox) conditions; the light orange graph represents a control in which AspH was replaced by buffer. **d** LC-MS/MS analysis of the relevant digestion fragment of hFX EGF1_39mer_ (aa 101–119), which is partially hydroxylated (~40%) after treatment with His_6_-AspH_315–758_, confirms AspH-catalysed hydroxylation takes place at Asp103_hFX_ as anticipated based on the AspH-substrate consensus sequence (fragments bearing β-hydroxy-Asp are in red). **e** LC-MS/MS analysis of the relevant digestion fragment of hFX EGF1_39mer_ (aa 101–119) before exposure to His_6_-AspH_315–758_

The discovery that specific asparagine- and aspartate-residues in epidermal growth factor-like domains (EGFDs) undergo hydroxylation was a landmark in post-translational modification (PTM) research, because it demonstrated proteins other than collagen type domains are directly modified by O_2_ (Fig. 1a)^1,2^. EGFD hydroxylation is catalysed by the aspartate/asparagine-β-hydroxylase (AspH, BAH), a non-haem ferrous iron and 2-oxoglutarate (2OG) oxygenase^3–5^, which produces succinate and carbon dioxide as co-products. Human *ASPH* (NCBI ID: 444 [https://www.ncbi.nlm.nih.gov/gene/?term=444], A*β*H-J-J locus) has two promoters and undergoes extensive alternative splicing^6,7^ resulting in >10 AspH isoforms (Supplementary Fig. 1). An *ASPH* mutation resulting in the R735W substitution correlates with Traboulsi syndrome (OMIM 601552/refSNP rs374385878), which manifests as facial dysmorphism and lens dislocation^8^; another mutation (G434V) affects human kidney function resulting in vesicoureteral reflux^9^. AspH and its truncated, likely non-catalytic, isoform Humbug are overexpressed in cancers^10–14^; *AspH* is strongly upregulated by hypoxia^15^, consistent with its clinical use as a tumour biomarker^16,17^. Loss of murine EGFD Asp/Asn β-hydroxylation correlates with increased tumour incidence and developmental defects similar to those caused by disrupted Notch signalling^18^. AspH localises to the endoplasmic reticulum (ER) in normal cells, but (at least part of AspH) localises to the surface of tumour cells^16,17^ and its hydroxylase activity was reported to enhance cell migration^19,20^.

EGFDs are ~40 residue domains often present in membrane protein extracellular regions; their structures are primarily comprised of β-strands stabilised by 3 conserved internal disulfides, which, as revealed by multiple crystal and NMR structures, normally adopt a thermodynamically preferred canonical Cys1–3, 2–4, 5–6 disulfide pattern^21–23^. This disulfide pattern is a functional requirement for some EGFDs^24^ and for Ca(II) ion binding^25–27^. AspH catalyses (3*R*)-hydroxylation of specific Asp/Asn residues in EGFDs, some of which are involved in binding Ca(II) in medicinally relevant proteins, e.g. as in coagulation factors, Notch, Jagged, and Fibrillins^28,29^.

There are about 60 human 2OG oxygenases with a variety of functional roles including in collagen biosynthesis, fatty acid metabolism/carnitine biosynthesis, nucleic acid repair/modification, O_2_ sensing, and transcriptional/translational regulation^30^. Some 2OG oxygenases are therapeutic targets, including those involved in carnitine biosynthesis and the hypoxia-inducible factor hydroxylases^31^. Structurally informed sequence alignments reveal most 2OG oxygenases possess a conserved His-Xxx-Asp/Glu…His Fe(II)-binding motif located on β-strands II and VII of the double-stranded β-helix (DSBH) core fold^32^. By contrast, sequences of AspH indicate a His-Xxx-Gly…His Fe(II)-binding motif^33^, though it has been unclear if an additional residue from another part of AspH could be involved in Fe(II) binding. Despite its importance in healthy biology and cancer, few biochemical studies and no efficient substrates of isolated recombinant AspH have been reported to date.

Here, we report crystallographic and biochemical results on the structure and substrate selectivity of AspH. The results reveal a key role for the TPR domain of AspH in substrate binding and, unexpectedly, that the preferred EGFD-substrates of AspH have a non-canonical (Cys3–4) disulfide pattern.

## Results

### AspH oxygenase domain structure

As precedented in work with other multi-domain 2OG oxygenases^34,35^, we initially prepared the isolated 2OG oxygenase domain of AspH for studies on catalysis. Recombinant N-terminally hexa-His-tagged (His_6_) AspH_562–758_ (AspH-Ox) was prepared in *Escherichia coli* (Supplementary Fig. 2) and tested for catalytic activity using the N-terminal EGFD of human coagulation factor X (hFX EGF1_39mer_; aa 86–124, Fig. 1b), a known cellular AspH substrate^2,36^, which was prepared by solid-phase peptide synthesis followed by thiol oxidation in air-saturated buffer to give disulfides. However, no hydroxylation of hFX EGF1_39mer_ was detected by mass spectrometry (MS). Using a [1-^14^C]-2OG based assay, we observed AspH-Ox catalysed turnover of 2OG to CO_2_ in the absence of substrate_,_ demonstrating oxygenase activity, albeit uncoupled from substrate hydroxylation as precedented with some other 2OG oxygenases under certain conditions (Supplementary Fig. 3).

To investigate the predicted unusual Fe(II)-binding motif of AspH, we initiated crystallographic studies. A structure of the AspH oxygenase domain (AspH-Ox) was determined with Ni(II), substituting for Fe(II), and with L-malate (from the crystallisation buffer) bound in the predicted 2OG binding pocket (Supplementary Fig. 4). As expected, the AspH-Ox structure is comprised of 8 β-strands forming a double-stranded beta-helix (DSBH) fold, with its active site located at the more open end of the β-sandwich. In contrast to most 2OG oxygenases, the active site metal is only coordinated by two histidine residues (His679, His725), located on DSBH strands II and VII. Two water molecules and a malate molecule complete octahedral coordination of the metal ion (Supplementary Fig. 4). Overall, the Asp-Ox structure validates the expected DSBH fold with intact Fe(II) and 2OG-binding sites, consistent with the observed 2OG turnover. To further investigate the lack of EGFD substrate hydroxylation by the isolated AspH-Ox domain, we produced a longer AspH construct containing the AspH-Ox and a predicted N-terminal tetratricopeptide repeat (TPR) domain.

### AspH oxygenase and TPR domain structure and activity

In contrast to AspH-Ox, the longer AspH_315–758_ construct containing both 2OG oxygenase and TPR domains (AspH-TPR-Ox, Supplementary Fig. 5) catalysed hydroxylation of hFX EGF1_39mer_ at the anticipated Asp-residue (Asp103_hFX_, Fig. 1c–e). However, incomplete substrate turnover (40%) was observed even with prolonged incubation times and at higher enzyme-to-substrate ratios using hFX EGF1_39mer_ synthesized by thiol oxidation in air-saturated buffer. We next set out to obtain crystal structures of AspH-TPR-Ox, in part aiming to rationalise the observed incomplete substrate hydroxylation.

**Fig. 2:**
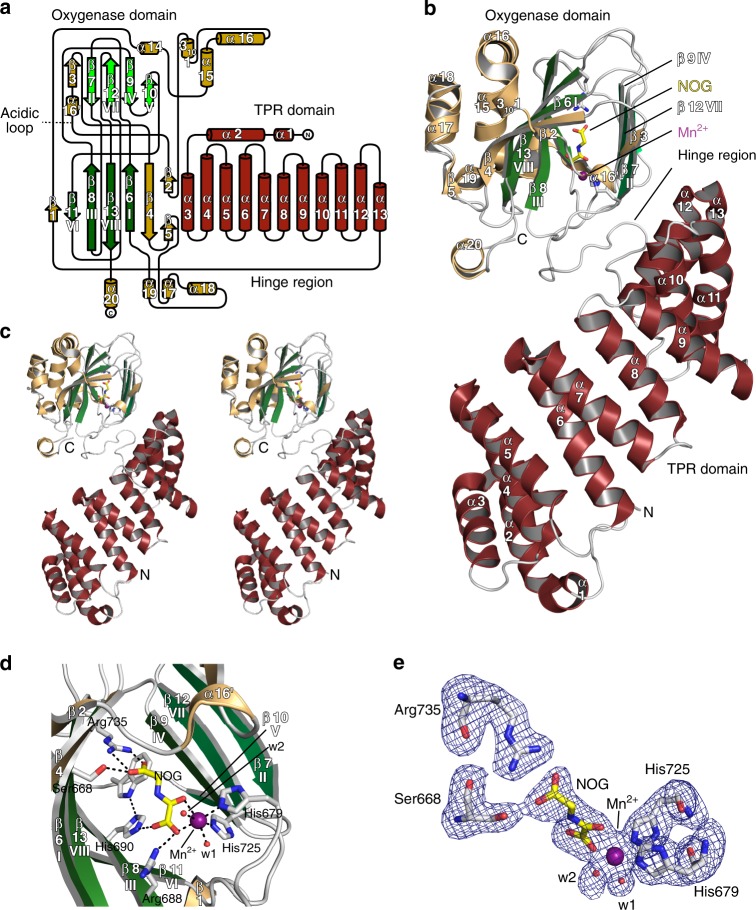
Crystal structure of AspH-TPR-Ox reveals Fe(II)-binding involving only two residues. Color code: grey: His_6_-AspH_315–758_; yellow: carbon-backbone of *N*-oxalylglycine (NOG); violet: Mn; red: oxygen; blue: nitrogen. w: water. **a** Topology diagram of human AspH_315–758_. **b** Overview of the AspH-TPR-Ox crystal structure. **c** Stereoview of the AspH-TPR-Ox crystal structure. **d** Close-up of the AspH-active site: Arg735 forms a salt bridge with the distal (C-5) carboxylate oxygens of NOG (2.4 and 3.1 Å) while Ser668 interacts with one oxygen lone pair through a hydrogen bond (2.7 Å). His690 is positioned to hydrogen bond to the C-1 carboxylate of NOG (2.8 Å). Mn(II) is bound to His679 (2.3 Å) and His725 (2.2 Å) of His_6_-AspH_315–758_ and coordinates two water molecules (both 2.2 Å) as well as the C-1 (2.2 Å) and C-2 (2.5 Å) carbonyl oxygens of NOG. **e** Representative OMIT electron density map (mFo–DFc) contoured to 3σ around NOG, Mn, water, and relevant AspH-active site residues are shown

The longer AspH-TPR-Ox construct was crystallised in the presence of Mn(II), substituting for the Fe(II) co-factor, and the 2OG analogue, *N*-oxalylglycine (NOG); L-malate was not included in the crystallization buffer (Fig. 2 and Supplementary Fig. 6). The oxygenase domain fold conformation in the AspH-TPR-Ox structure is near identical to that of the AspH-Ox structure (main chain RMSD 0.20–0.22 Å), with the largest conformational differences occurring in an acidic loop (aa 614–620) which forms part of the active site (Supplementary Figs. 7 and 8).

The solenoid-like TPR domain (aa 330–555) comprises 6 tandem repeating pairs of anti-parallel helices (12 helices in total, *α*1–*α*12) and is connected to the AspH-Ox domain by a hinge-loop region (aa 556–577) (Fig. 2a–c). Like that of AspH-Ox, the AspH-TPR-Ox structure shows His679, His725, and two waters coordinating the metal, but instead of malate, NOG is bound in the 2OG binding pocket. The NOG C-5 carboxylate is positioned to salt bridge with Arg735 and to hydrogen-bond with the Ser668 hydroxyl (which form a “RXS motif” as observed in some other 2OG oxygenases)^31^. The NOG oxalate is bound to the active site via bidentate metal coordination and forms hydrogen bonds with the side chains of His690 and Arg688 (Fig. 2d, e). This structure indicates that the R735W substitution, present in a Traboulsi syndrome patient^8^, likely interferes with 2OG binding.

The major β-sheet of the DSBH core is flanked by seven helices (*α*13–*α*19, Fig. 2a, b); the minor β-sheet is partially flanked by two helices derived from the C-terminal repeats (repeat 6) of the TPR domain. The 5 N-terminal TPR repeats form a right handed superhelix, the concave face of which partially aligns to the shorter linking loop end of repeats 2–4 that point towards the oxygenase domain and help to form the active site (Fig. 2b, c). The hinged oxygenase-TPR domain arrangement forms a large and open cavity, in which we envisaged EGFD substrates may fit. Comparison of the AspH TPR domain with other TPR domain structures identified by a DALI search reveals variations in the extent of curvature, likely due to the differences in intra-repeat interactions and loop lengths. The AspH TPR repeats 1–5 align particularly well with 5 repeats of the *Candidatus magnetobacterium bavaricum* magnetosome-associated (MAMA) TPR-containing protein (RMSD over 117 Cα atoms: 3.7 Å)^37^. The AspH-TPR-Ox structure suggested that the TPR domain may be directly involved in substrate binding. To investigate, we crystallized AspH-TPR-Ox in the presence of the hFX EGF1_39mer_ substrate.

**Fig. 3:**
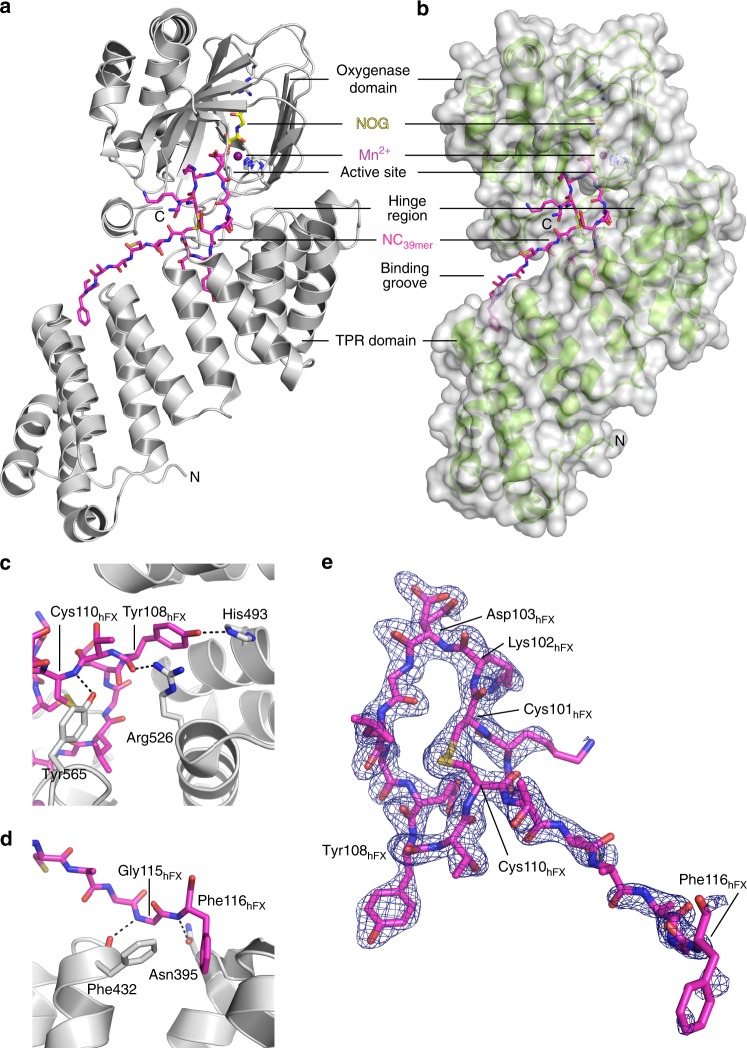
Structure of AspH-TPR-Ox:hFX features a non-canonical EGFD disulfide connectivity. Color code: magenta: carbon-backbone of NC_39mer_ peptide; yellow: carbon-backbone of *N*-oxalylglycine (NOG); violet: Mn; red: oxygen; blue: nitrogen; pale yellow: sulfur. **a** Overview of the AspH-TPR-Ox:hFX structure; His_6_-AspH_315–758_ in grey. **b** Surface representation (grey) of the AspH-TPR-Ox:hFX structure showing the substrate-binding groove, His_6_-AspH_315–758_ in green. **c** Interactions of the TPR domain residues His493 and Arg526 as well as the hinge region residue Tyr565 with the NC_39mer_ peptide. **d** Additional interactions of the TPR domain with the NC_39mer_ peptide: Asn395 forms a hydrogen bond with Phe116_hFX_ (3.0 Å) and Phe432 forms a hydrogen bond with Gly115_hFX_ (3.2 Å). **e** OMIT electron density map (mFo–DFc) contoured to 3σ around the hFX derived NC_39mer_ peptide supports the presence of a non-canonical disulfide bridge between Cys101_hFX_ and Cys110_hFX_ as a substrate requirement for AspH (canonical EGFD disulfide isomer: Cys1–3, 2–4, 5–6; non-canonical EGFD disulfide isomer: Cys1–2, 3–4, 5–6; see Fig. 5a). Note that electron density for two alternative conformation of the Asp103_hFX_ side chain is observed

**Fig. 4:**
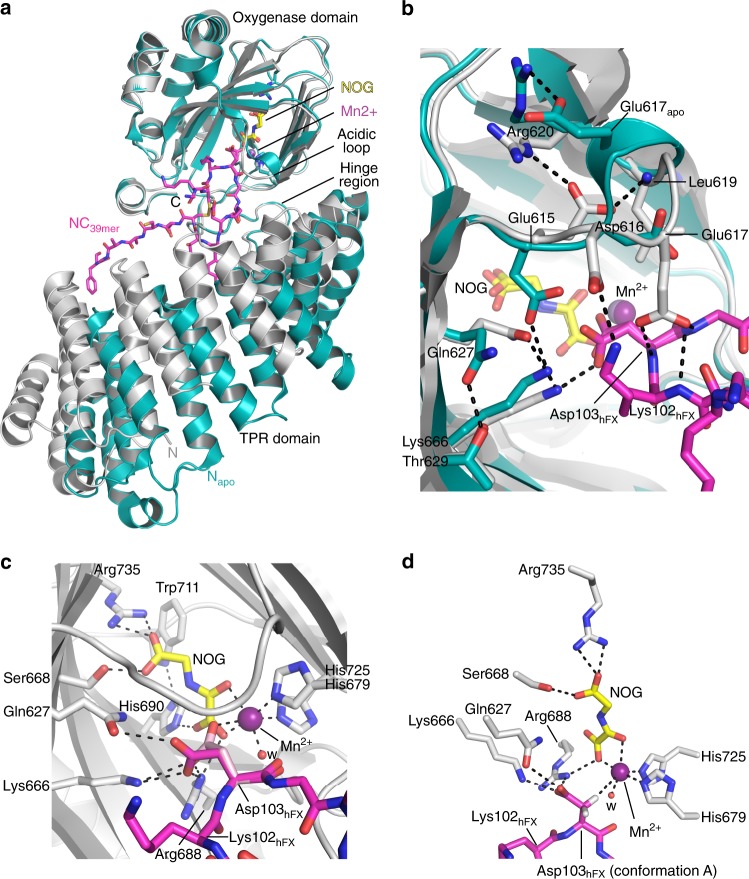
Significant conformational changes occur in AspH upon substrate binding. Color code: grey: His_6_-AspH_315–758_ (AspH-TPR-Ox:hFX); turquoise: His_6_-AspH_315–758_ (AspH-TPR-Ox); magenta: carbon-backbone of NC_39mer_ peptide; yellow: carbon-backbone of *N*-oxalylglycine (NOG); violet: Mn; red: oxygen; blue: nitrogen; pale yellow: sulfur. w: water. **a** Superimposition of the AspH-TPR-Ox:hFX (grey) and AspH-TPR-Ox (turquoise) structures indicate conformational changes in the TPR domain, the hinge region, and the oxygenase acidic loop on substrate binding. **b** Superimposition of the AspH-active sites of the AspH-TPR-Ox:hFX (grey) and AspH-TPR-Ox (turquoise) structures: The interaction between Glu617_apo_ and Arg620 (2.5 Å) in the AspH-TPR-Ox structure is lost on substrate binding; both Asp616 and Glu617 interact with the substrate in the AspH-TPR-Ox:hFX structure. Gln627 (3.2 Å) and Lys666 (2.7 Å) bind to the Asp103_hFX_ carboxylate of the active conformer of the AspH-substrate. On substrate binding, the side chain of Glu615 rotates by ~90° to interact with the side chain of Arg620 (2.8 Å) and the main chain of Leu619 (2.8 Å), rather than Lys666 (2.7 Å) as in the substrate unbound state. **c** The side chain of the Asp103_hFX_ residue undergoing hydroxylation is observed in two conformations (A: magenta and B: pink; see Supplementary Fig. 10 for details). The Asp103_hFX_ side chain carboxylate of conformation B (pink) is positioned (2.6 Å) to interact with the Mn. **d** Close-up of the AspH-active site: The *pro-R* hydrogen at the Asp103_hFX_ β-position of the likely productive NC_39mer_ conformation A (magenta) is positioned to interact with the Mn (distance Cβ-Mn: 4.2 Å), consistent with hydroxylation at this position

AspH-TPR-Ox was successfully co-crystallized with a 39-residue fragment of hFX EGF1 (AspH-TPR-Ox:hFX, Fig. 3 and Supplementary Fig. 9). The AspH-TPR-Ox structure reveals electron density at the active site corresponding to 18 of the 39 hFX EGF1 substrate residues (aa 99–116: -GKC_3_K**D**GLGEYTC_4_TC_5_LEGF-) and for a disulfide linkage between Cys101_hFX_ and Cys110_hFX_ (Fig. 3e). Highly specific interactions, including multiple protein–protein/peptide interactions with both AspH-Ox and TPR domains are apparent (Fig. 3b–d). The N-terminal region of hFX (aa 100–105) interacts with the AspH-Ox domain and the C-terminal region of hFX (aa 106–116) interacts with the TPR domain. Comparison of AspH structures with and without substrate implies that induced fit involving substantial conformational changes during formation of the AspH-TPR-Ox:hFX protein:protein complex occurs; the distance between Leu433 on repeat 3 of the TPR domain to Pro756 of the oxygenase domain near the C-terminus is reduced from 20 to ~14 Å on substrate binding. This movement is apparently enabled by the hinge region (aa 556–577) linking the TPR and AspH-Ox domains (Fig. 4a, b).

The side chain of Asp103_hFX_, the substrate asparagine undergoing hydroxylation, is at the i+2 position of a Type I β-turn adjacent to the active site metal and was modelled in two conformations (A,B; see Supplementary Fig. 10 for details), both of which are positioned to form salt bridges with the side chains of Arg688 and Lys666 (Figs. 4c and 3e). The Asp103_hFX_ carboxylate side chain in conformation A is positioned to form a hydrogen bond (3.2 Å) with the side chain of Gln627, whilst in conformation B the carboxylate is positioned to interact with the active site metal (2.6 Å) *trans* to His725. Asp103_hFX_ conformation A is poised for C-3 hydroxylation such that its *pro*-3*R*-hydrogen projects towards the metal (Cβ-metal distance ~4.5 Å) (Fig. 4d), consistent with reported (3*R*)-stereoselectivity for AspH catalysis^1,36^. The Asp103_hFX_ conformation B displaces one of the two metal coordinating waters that are observed in the AspH-TPR-Ox structure without bound substrate.

Substrate binding to AspH also involves major changes within an acidic loop which forms part of the active site (aa 614–620), with the side chain carboxylates of Glu615, Asp616 and Glu617 all being shifted towards the substrate (Fig. 4b). The Glu617 side chain is positioned to form a hydrogen bond (2.8 Å) with the main chain amide nitrogen of Asp103_hFX_; the Asp616 side chain is positioned to form a salt bridge with the Lys102_hFX_ side chain; and Glu615 folds into the inner apex of the acidic loop forming a salt bridge with Arg620 and a hydrogen bond with the main chain amide nitrogen of Leu619. In the AspH-Ox structure, the side chain of Glu617 appears to mimic the Asp103_hFX_ side chain interactions with Lys666 and Arg688, possibly reflecting a function for this residue in protecting the active site from oxidation in the absence of substrate. In AspH-TPR-Ox:hFX structure, the Arg686 side chain is positioned to form a hydrogen bond with the main chain carbonyl oxygen of Lys102_hFX_ and a salt bridge with the Asp721 side chain. The position of the side chains of Arg686, Arg688 and Asp721 in AspH-TPR-Ox:hFX does not change in comparison with AspH-TPR-Ox.

### Role of the TPR domain

Notably, the highly-conserved hFX tyrosine, Tyr108_hFX_, that is part of the consensus EGFD sequence for AspH hydroxylation^38^, binds in a mostly hydrophobic pocket located between TPR repeats 5 and 6 located on the concave surface of the TPR domain. The Tyr108_hFX_ side chain hydroxyl is positioned to form a hydrogen bond to the His493 side chain Nε2 (3.0 Å) and its main chain carbonyl to form a hydrogen bond (2.8 Å) with the Arg526 side chain. The Tyr108_hFX_ side chain is apparently positioned to form skewed π-π-stacking interactions with the side chains of Phe496 and Arg526. The main chain nitrogen of Cys110_hFX_ (2.9 Å) interacts with the Tyr565 side chain (Fig. 3c).

At the hFX C-terminus, the phenyl ring of Phe116_hFX_ is buried in a hydrophobic pocket on the TPR domain formed by Ala389, Leu398, and Phe432, which are located between TPR repeats 2 and 3. The phenyl ring of Phe116_hFX_ is positioned to π-stack with the Arg393 guanidine side chain and the peptide bonds linking Arg393 with Ser394 and Asn395 (Fig. 3d). The Phe116_hFX_ main chain amide NH forms a hydrogen bond with the Asn395 side chain Oδ1 (2.6 Å). Sequence alignment of known hydroxylated EGFDs shows that the aromatic residues at the Phe116_hFX_ position are highly conserved (Supplementary Fig. 11).

Unexpectedly, in the AspH-TPR-Ox substrate complex we observed a non-canonical disulfide bridge, i.e. between Cys101_hFX_ and Cys110_hFX_ for the hFX substrate, in effect forming a ten-residue ring (Fig. 3e). The substrate disulfide is positioned in a hydrophobic pocket formed by Tyr565, Pro682, and Ile758. This pocket is shared with Leu105_hFX_ and expanded by Phe529 and Leu564 of AspH.

The observed hFX disulfide corresponds to a non-canonical Cys3–4 EGFD disulfide link, suggesting this pattern is required for AspH catalysis rather than the established (Cys1–3, 2–4, 5–6) disulfide pattern. The canonical EGFD disulfide pattern (Cys1–3, 2–4, 5–6) has been observed in all reported crystal and NMR structures of EGFDs bearing the AspH-substrate consensus sequence, including for hFX^39,40^. A non-canonical disulfide pattern (Cys1–2, 3–4, 5–6) has been reported in crystalline and solution state studies on the EGFD-5 of human thrombomodulin^41–43^; this, however, does not have the AspH substrate consensus sequence. MS^44^ and computational^45^ work also suggests the possibility of a non-canonical disulfide connectivity pattern for EGFDs bearing the consensus sequence for AspH-catalysed hydroxylation. It was, therefore, important to investigate AspH activity towards disulfide patterns with the non-canonical 3–4 link (i.e. Cys1–2, 3–4, 5–6; 1–5, 3–4, 2–6; or 1–6, 2–5, 3–4).

### AspH accepts an unexpected EGFD disulfide pattern

**Fig. 5:**
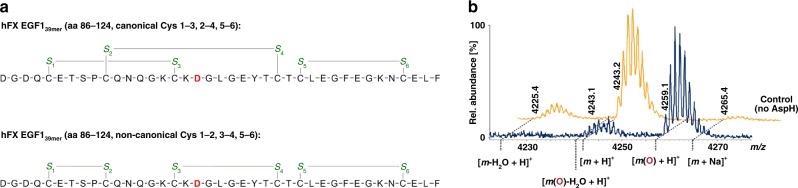
AspH fully hydroxylates a mixture of canonical and non-canonical EGFD disulfides under redox conditions. End-point turnover reactions were performed as in the Methods Section. **a** Schematic structures of the two major hFX EGF1_39mer_ disulfide isomers identified in a single batch of hFX EGF1_39mer_ obtained by thiol oxidation in air-saturated buffer (see Supplementary Information); disulfides are in green (canonical isomer, Cys1–3, 2–4, 5–6, top; non-canonical isomer, Cys1–2, 3–4, 5–6, bottom), the hydroxylation sites (Asp103_hFX_) are in red. **b** >95% Hydroxylation of hFX EGF1_39mer_ was observed under redox conditions as opposed to partial hydroxylation under standard (non-redox) conditions (Fig. 1c), indicating that a ‘non-canonical’ EGFD-disulfide pattern (Cys 1–2, 3–4, 5–6) is the actual AspH-substrate; the light orange graph represents a control in which AspH was replaced by buffer

MS analyses imply that the disulfide pattern arising from thiol oxidation of the linearly synthesised hFX EGF1_39mer_ in air-saturated buffer predominantly comprises a mixture of the canonical (Cys1–3, 2–4, 5–6) and non-canonical (Cys1–2, 3–4, 5–6) disulfide patterns (Fig. 5a); other additional disulfide isomers may be present in the mixture but were not detected. The canonical and non-canonical disulfide isomers co-elute as a single peak in MS coupled HPLC (Supplementary Figs. 12 and 13). Similar results have been reported for the synthesis of EGFD5 of human thrombomodulin^46^. The hFX EGF1_39mer_ mixture underwent partial hydroxylation (~40%, Fig. 1c) by AspH-TPR-Ox under normal assay conditions. However, it underwent complete hydroxylation (>95%) when incubated with enzyme in a redox buffer containing a mixture of reduced and oxidized glutathione (GSH and GSSG). The conditions were employed to enable reversible thiol-disulfide interchange of the canonical and non-canonical hFX forms (Fig. 5b).

**Fig. 6:**
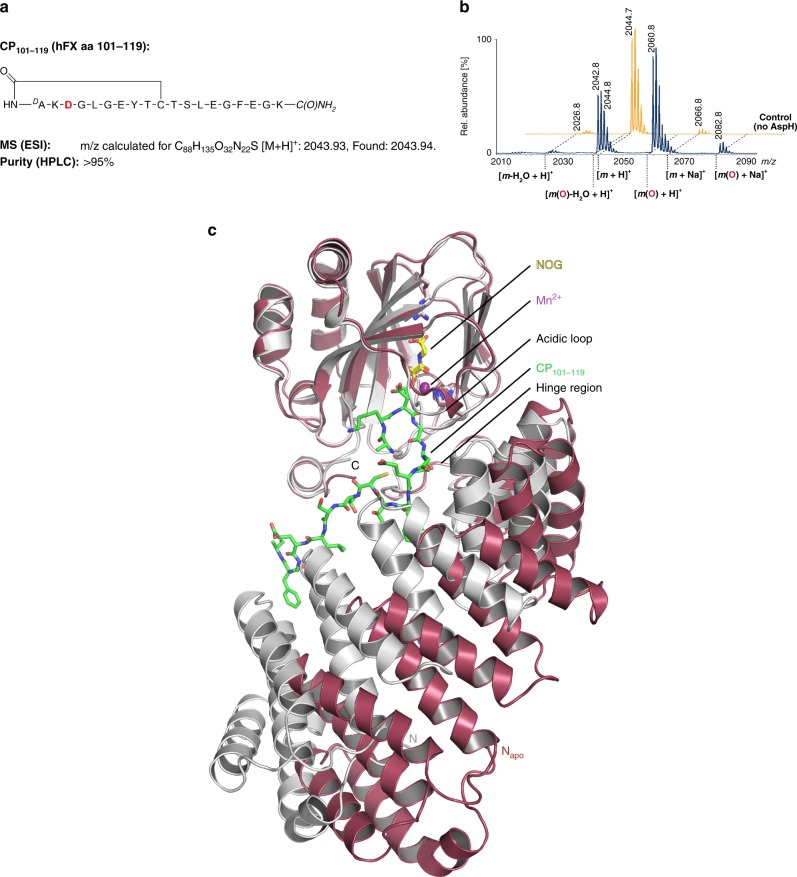
A cyclic peptide mimicking the non-canonical Cys3–4 EGFD is an excellent AspH-substrate. End-point turnover reactions were performed as in the Methods Section. **a** Schematic structure and calculated mass of the cyclic peptide CP_101–119_ mimicking the central macrocyclic disulfide of the non-canonical isomer of hFX EGF1_39mer_ (Cys3–4); the hydroxylation site (Asp103_hfX_) is in red. **b** >95% Hydroxylation of the CP_101–119_ peptide was observed under standard (non-redox) conditions; the light orange graph represents a control in which AspH was replaced by buffer. **c** Two His_6_-AspH_315–758_-molecules are present in the asymmetric unit of the AspH-TPR-Ox:CP_101–119_ crystal structure, only one binds the CP_101–119_ peptide (details in the Supplementary Information): The superimposition of the two independent AspH-molecules of this crystal structure (colour code: unbound AspH: raspberry; AspH bound to CP_101–119_: grey; CP_101–119_: green) highlights an induced fit mechanism of AspH upon substrate binding: Major conformational changes occur in the TPR domain, the hinge region and the acidic loop whilst the overall oxygenase domain conformation is hardly affected. The thioether linker of the cyclic peptide could not be accurately modelled into the electron density; however, complementary analytical methods imply the presence of a cyclic thioether linkage (see Supplementary Figure 16 for further details)

The complexities of the reversible nature of thiol-disulfide interchange in solution render definitive identification of the preferred disulfide pattern of the AspH EGFD substrate difficult. We thus synthesised an irreversibly-linked cyclic analogue of the Cys3–4 EGFD disulfide substrate; A 10mer cyclic peptide (CP_101–110_) comprising the core residues (aa 101–110) of hFX EGF1_39mer_, wherein the Cys3–4 disulfide was replaced by a stable thioether, was prepared using the method of Suga et al.^47,48^. CP_101–110_ was efficiently Asp-β-hydroxylated by AspH-TPR-Ox (~85%, Supplementary Figs. 14a and 15), whereas cyclic peptides with variations in consensus sequence residues or a linear 10mer peptide were not (Supplementary Fig. 14), further supporting the crystallographically observed Cys3–4 disulfide as the preferred substrate form for AspH catalysis. An extended 19mer CP (CP_101–119_, Fig. 6a) was designed based on the enzyme-substrate contacts observed in the AspH-TPR-Ox:hFX structure and found to be a better substrate than CP_101–110_ (>95%, Fig. 6b). A structure of AspH co-crystallised with the CP_101–119_ (AspH-TPR-Ox:CP_101–119_) reveals this substrate bound to one of the two AspH molecules in the asymmetric unit (chain A). Conformational differences between the bound (chain A) and unbound (chain B) (Cα RMSD 3.82 Å) are consistent with afore-described observations implying an AspH induced fit mechanism with hFX (Fig. 6c and Supplementary Fig. 16).

### Substrate variant analysis

**Fig. 7:**
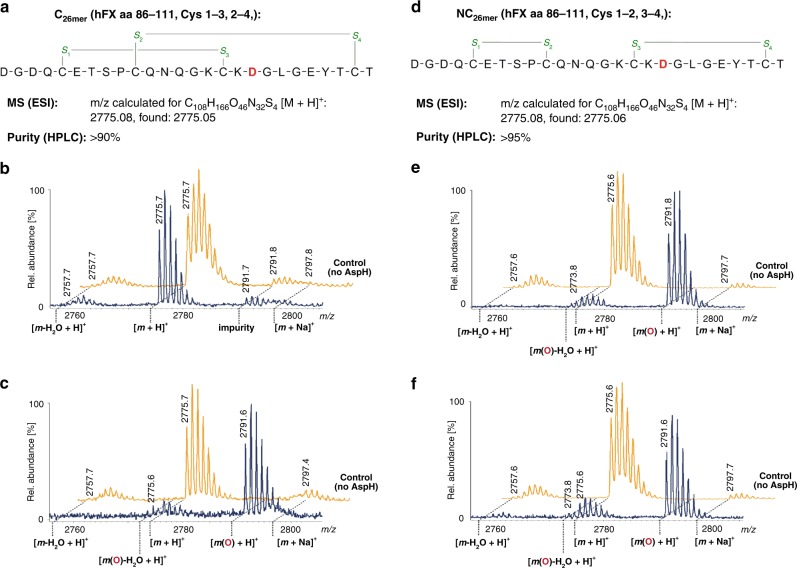
The EGFD disulfide connectivity determines the degree of AspH-catalysed hydroxylation under non-redox conditions. End-point turnover reactions were performed as in the Methods Section. Light orange graphs represent controls in which AspH was replaced by buffer. **a** Schematic structure and calculated mass of the C_26mer_ peptide, bearing a canonical disulfide arrangement (Cys1–3, 2–4); disulfides are shown in green, the hydroxylation site (Asp103_hFX_) is indicated in red. **b** No hydroxylation of the C_26mer_ peptide was observed under standard (non-redox) conditions; ~7% impurity (might correspond to an oxidized byproduct) was observed in this sample, including the control samples. **c** >95% Hydroxylation of the C_26mer_ peptide was observed under redox conditions. **d** Schematic structure and calculated mass of the NC_26mer_ peptide, bearing a non-canonical disulfide arrangement (Cys1–2, 3–4); disulfides are in green, the hydroxylation site (Asp103_hFX_) is in red. **e** >95% Hydroxylation of the NC_26mer_ peptide was observed under standard (non-redox) conditions. **f** ~83% Hydroxylation of the NC_26mer_ peptide was observed under redox conditions

**Fig. 8:**
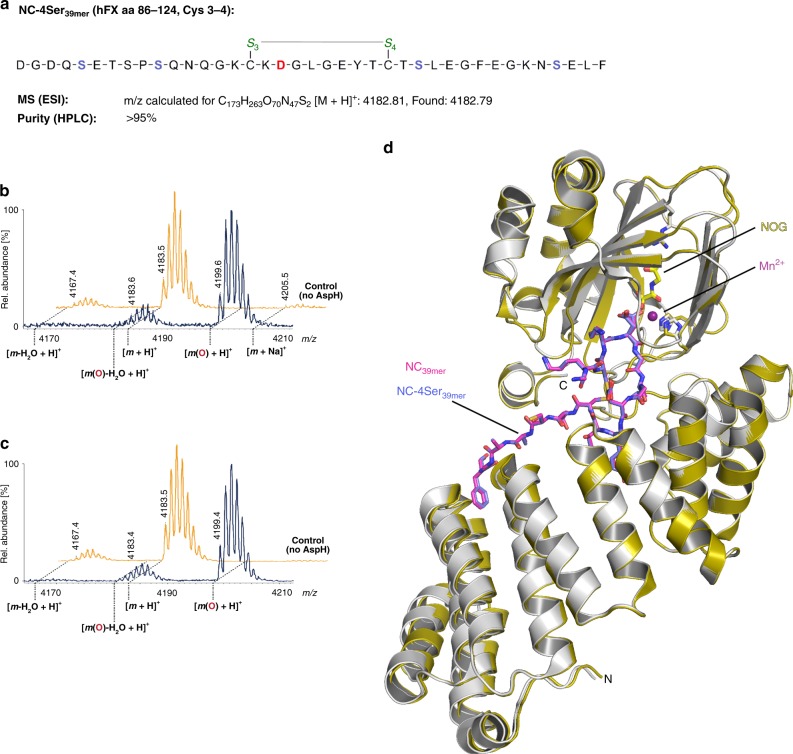
The AspH-substrate requirement is a ring composed of 10 amino acid residues. End-point turnover reactions were performed as in the Methods Section. Light orange graphs represent controls in which AspH was replaced by buffer. **a** Schematic structure and calculated mass of the NC-4Ser_39mer_ peptide featuring a single disulfide between Cys3–4 (green); its sequence is identical to the one of hFX EGF1_39mer_ (see Fig. 1a), except that Cys90_hFX_, 95, 112, 121 are substituted for Ser (light blue) to avoid disulfide scrambling; the hydroxylation site (Asp103_hFX_) is in red. **b** >95% Hydroxylation was observed under standard (non-redox) conditions. **c** >95% Hydroxylation was observed under redox conditions. **d** Superimposition of the AspH-TPR-Ox:hFX crystal structure (color code: AspH: grey; NC_39mer_ peptide: magenta) with the AspH-TPR-Ox:NC-Ser_39mer_ crystal structure (color code: AspH: gold; NC-4Ser_39mer_ peptide: slate blue) shows a high conservation of the conformations of both enzyme and ligands

Next, we assayed AspH for its ability to hydroxylate truncated 26mer hFX EGF1 substrates (aa 86–111) lacking the Cys5–6 disulfide-forming residues, synthesized using orthogonal cysteine protection to yield selectively both the pure canonical (C_26mer_, Fig. 7a) or non-canonical (NC_26mer_, Fig. 7d) disulfide isomers. Only the NC_26mer_ peptide was hydroxylated under normal assay conditions (Fig. 7b, e), whereas both 26mer peptides were hydroxylated using redox buffer conditions (Fig. 7c, f). A structure of AspH in complex with NC_26mer_ was obtained (AspH-TPR-Ox:NC_26mer_, Supplementary Fig. 17). Comparison of AspH-TPR-Ox:NC_26mer_ and AspH-TPR-Ox:hFX structures reveals little overall difference (Cα RMSD 0.13 Å). The presence of a Cys3–4 disulfide bond indicates that the additional residues present in the longer 39mer substrate, which were not observed in electron density maps of AspH-TPR-Ox:NC_26mer_ due to disorder, are likely not essential for substrate recognition. The efficient hydroxylation of the synthetic hFX EGF1_39mer_ with cysteines 1, 2, 5 and 6 (aa 90, 95, 112 and 121, respectively) all being replaced by serines (NC-4Ser_39mer_) further supports the proposal that only the substrate Cys3–4 disulfide is required for AspH-catalysed hydroxylation (Fig. 8a–c). A structure of AspH in complex with the NC-4Ser_39mer_ substrate was obtained (AspH-TPR-Ox:NC-Ser_39mer_, Supplementary Fig. 18). Comparison of the AspH-TPR-Ox:NC-Ser_39mer_ and AspH-TPR-Ox:hFX structures reveals little overall difference (Cα RMSD 0.08 Å, Fig. 8d), including for the Ser112_NC-Ser39mer_ and Cys112_hFX_ residues, suggesting that Cys112_hFX_ may not necessarily form a disulfide during productive AspH-catalysis.

### AspH with other EGFD protein substrates

To investigate the generality of the hFX results, we tested recombinant Ca(II)-binding EGFDs (cbEGFDs) (~10–15 kDa) from human Fibrillin-1 (hFib1); a two EGFD containing construct, cbEGF32-33^49^, a three EGFD construct, cbEGF41-43^50^, and the single EGFD construct, TB4cbEGF23 (with a cbEGFD preceded by a transforming growth factor *β*-binding protein-like (TB) domain)^51^, and a three EGFD construct, cbEGF11-13, of Notch-1 (hNotch1)^52^. All these were hydroxylated by AspH-TPR-Ox (30–85%) under redox buffer conditions as determined by MS after proteolytic digestion, but were much poorer substrates under non-redox conditions (0–30%) (Supplementary Fig. 19).

### Effect of calcium ions on EFGD stability and AspH activity

**Fig. 9:**
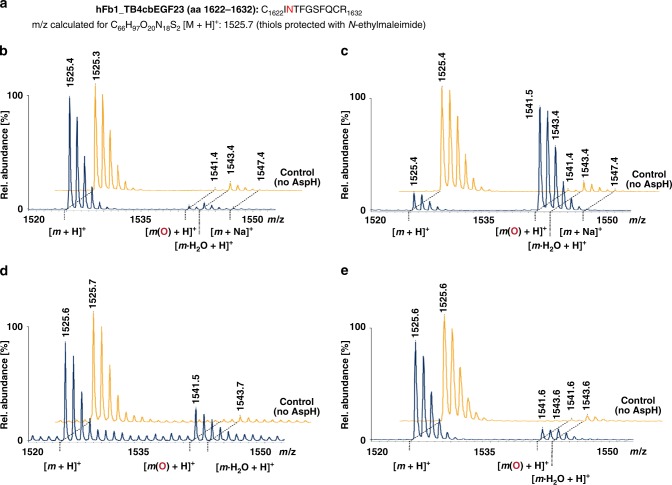
Ca(II) ions inhibit AspH-catalysed hydroxylation of a human fibrillin-1 fragment. End-point turnover reactions were performed with an incubation time of 180 min (See Methods Section). Prior to analysis, the substrate was reduced and cysteine thiols were derivatized with *N*-ethylmaleimide, then digested using trypsin, and analysed using MALDI-ToF-MS. Light orange graphs represent controls in which AspH was replaced by buffer. **a** Sequence of the fragment of human fibrillin-1, TB4cbEGF23 (hFIB1_TB4cbEGF23, aa 1527–1647; Uniprot Database entry: FBN1_HUMAN), bearing the hydroxylation site (Asn1624_hFIB1_, red). **b** <5% Hydroxylation was observed under standard (non-redox) conditions. **c** ~85% Hydroxylation was observed under redox conditions. **d** ~25% Hydroxylation was observed under redox conditions in the presence of 1 mM Ca(II). **e** ~10% Hydroxylation was observed under redox conditions in the presence of 3 mM Ca(II)

As most AspH-substrate EGFDs bear a Ca(II)-binding site nearby the AspH-hydroxylation site^28^, we next investigated if Ca(II) ions inhibit EGFD hydroxylation, i.e. by stabilising the canonical EGFD fold which is apparently not an AspH-substrate. No effect on AspH-TPR-Ox activity was observed on addition of 3 mM Ca(II) ions with any of the tested substrates in the absence of redox buffer. By contrast, in the presence of redox buffer, hydroxylase activity was not significantly affected for the recombinant multi-domain proteins hFib1 (cbEGF32–33 and cbEGF41–43) and hNotch 1 (cbEGF11–13). However, AspH hydroxylase activity was substantially inhibited for the recombinant hFib1 fragment, TB4cbEGF23 (aa 1527–1647, Fig. 9a), which binds Ca(II) tightly (*K*_d_ = 16 ± 1 nM)^51^ and which is a poor AspH substrate under non-redox hydroxylation conditions (<5% hydroxylation, Fig. 9b), but an efficient one in redox buffer (~85% hydroxylation, Fig. 9c). The addition of Ca(II) ions at 1 mM final concentration in redox buffer reduced the extent of TB4cbEGF23 hydroxylation from ~85 to ~25%; in the presence of 3 mM Ca(II) ions in redox buffer TB4cbEGF23 hydroxylation was further reduced to ~10% (Fig. 9d, e). No inhibitory effect was observed when 3 mM Mg(II) was added instead of Ca(II) (Supplementary Fig. 20a). Ca(II) ions do not directly inhibit AspH, as the irreversibly formed cyclic thioether, CP2_101–110_, was fully hydroxylated in the presence of 10 mM Ca(II) (Supplementary Fig. 20b).

Since Ca(II) ions preferably bind to and stabilise the canonical EGFD fold^27^, these results further support assignment of the non-canonical 3–4 EGFD disulfide form as a preferred AspH substrate. The effects of the neighbouring domains and the different intrinsic Ca(II) affinities of EGFD proteins complicates the understanding of the influence of Ca(II) ions on AspH-catalysis. Nonetheless, the results clearly reveal the potential for regulation of AspH activity by Ca(II) ions by stabilisation of cbEGFDs in the canonical disulfide pattern fold, so making them unavailable for disulfide shuffling to the non-canonical Cys3–4 pattern.

## Discussion

There is an unmet need for further investigations into the biological functions of human AspH and its role in genetic diseases and cancer. Such work is challenging given the membrane bound nature of AspH^5^, the presence of multiple different isoforms/splice variants (>10 in humans)^6,7^, its location in the ER^5^, and the redox sensitive nature of its disulfide substrates^53^. Our work defining the first substrates for isolated AspH and the roles for the TPR domain in its catalysis provides a structural basis for future work on the cellular and physiological roles of AspH.

Whilst the oxygenase domain fold of AspH is typical for the 2OG oxygenase superfamily (Fig. 2), details of its active site and the role of the TPR domain in EGFD-substrate binding are remarkable. Key insights arising from our structures include: the presence of an active site metal bound by only two protein ligands, the role of the TPR domain in substrate binding, and the fact that AspH only accepts EGFD-substrates with the non-canonical Cys3–4, instead of the canonical Cys1–3, 2–4, 5–6, disulfide pattern. The latter finding was unexpected given the prevalence of the canonical EGFD disulfide pattern (at least outside of the ER) reported in the literature^54^. The lack of in vitro hydroxylation assays for AspH has hindered progress on its characterisation; our results will enable further detailed kinetic, mechanistic, and inhibition studies on AspH.

Comparison of the AspH structures with and without substrates implies substantial induced fit during substrate binding (Fig. 4a, b); experience with NMR studies on single oxygenase domain 2OG oxygenases^55–57^ implies that in the case of AspH, even more substantial changes in conformation may occur in solution, likely involving both oxygenase and TPR domains, as well as AspH-substrates. Whatever the precise extent of conformational changes during catalysis, the results reveal a central role for the TPR domain in enabling AspH catalysis—without it, 2OG turnover occurs, but EGFDs are not hydroxylated (Fig. 4 and Supplementary Fig. 3).

TPR domains are involved in protein-protein interactions and substrate recognition in other proteins/enzymes^58^ including human 2OG oxygenases, i.e. the procollagen C-4 (CP4Hs) and C-3 (Leprecans) prolyl- and C-5 (PLODs) lysyl-hydroxylases, which contain (or are predicted to contain) TPR domains *N*-terminal to the oxygenase domain^59^. As yet, there are no structures reported for the oxygenase domains of eukaryotic members of these TPR containing enzymes, which like AspH are ER localised, though the TPR domain of CPH in complex with a collagen-like peptide has been structurally characterised^60^. Our work reveals how the AspH oxygenase and TPR domains recognise and bind a non-canonical 3–4 disulfide EGFD and, through conformational changes to the enzyme, guide and position the Asp/Asn residue to be hydroxylated to the active site in a catalytically productive manner. It would seem probable that some of the TPR domains in other ER-localised 2OG oxygenases have analogous roles.

The role of the TPR domain in AspH catalysis is precedented in the roles of some non-catalytic domains in substrate targeting by the 2OG dependent JmjC *N*^ε^-methyl lysine histone demethylases (KDMs), though this is only understood in detail from a structural perspective for two KDM7 subfamily members (KDM7A/B)^61^. Binding of a highly-conserved EGFD tyrosine (e.g. Tyr108_hFX_) to a hydrophobic pocket on the TPR domain surface is reminiscent of H3K4me3 binding to the plant homeobox domain (PHD) of KDM7A^61^. Analogous recognition domains to that of KDM7A/B occur in other JmjC KDMs, involving different domain types (e.g. Tudor domains), though to our knowledge TPR domains are not present in the chromatin modifying 2OG oxygenases; they thus appear to be characteristic of the ER localised 2OG oxygenases.

The combined results reveal that AspH accepts EGFD substrates with a non-canonical Cys3–4 disulfide pattern, rather than the canonical Cys1–3, 2–4, 5–6 pattern. Evidence for this comes from structural data, MS turnover assays under different redox conditions, inhibition by Ca(II) ions (which presumably bind and stabilise the canonical but not the non-canonical disulfide pattern), and the use of a stable disulfide cyclic peptide analogue. These observations are interesting because they raise the possibility of EGFDs manifesting dynamic disulfide patterns in the redox active environment of the ER, rather than being limited to the canonical form normally observed by in vitro biophysics and which current evidence implies would likely be dominating in the oxidising extracellular environment. Indeed, the combined crystallographic evidence suggests the canonical form is the most thermodynamically stable^21–23^. AspH could act as a chaperone regulating EGFD folding or recognising misfolded EGFDs, possibly as part of the redox sensitive unfolded protein response (UPR) pathway^62^. This is consistent with the identification of various degrees of β-hydroxylation in purified native proteins containing EGFDs, most notably human coagulation proteins hFIX (30%) and hFX (100%)^63^, which implies they have been through a folding cycle prior to secretion into the bloodstream. Interestingly, purified hFVII does not show any β-hydroxylation, despite having the AspH-substrate consensus sequence, which suggests a possible role of AspH in recognising misfolded rather than functionally active folded EGFDs. One role of AspH could be to regulate EGFD folding with hydroxylation being an oxygen sensitive element of this process rather than an end in itself; this proposal is consistent with the lack of observed effect of Asn/Asp hydroxylation on the canonical EGFD fold conformation and on Ca(II) ion binding^63^. Extracellular AspH could play a role in recognising and labelling mis-folded EGFDs on the cell surface, possibly in a manner relating to over-production of AspH in cancer cells^10,11,13,16,17^, since the latter can have dysregulated EGFD proteins involved in signalling (e.g. Notch)^64,65^. The structures reported here may also of be interest with respect to defining factors involved in AspH localisation, in particular to the surfaces of some tumour cells^16,17^. They raise the possibility of AspH localisation being regulated by EGFD binding in a manner dependent on the EGFD disulfide pattern and redox environment. The results also suggest that AspH activity may be regulated by Ca(II) ions; in this regard it is interesting that there are predicted Ca(II) binding EF-hand motifs in human AspH itself.

The results clearly reveal that, at least under the analysed conditions, AspH has an unusual Fe(II) coordination chemistry for a 2OG-dependent hydroxylase (Fig. 2d, e). It should be noted that all the AspH crystal structures presented here are in complex with Ni(II) or Mn(II) and a 2OG mimetic, i.e. NOG or L-malate. We considered the possibility that in the presence of Fe(II) and 2OG a third protein-derived ligand interacts with the metal. However, this scenario  seems unlikely given that the metal coordination geometries and substrate positions observed in many other 2OG-dependent oxygenase crystal structures in complex with Fe(II) and 2OG compared to Ni(II)/Mn(II) and NOG are very similar, with no evidence for additional enzyme derived ligands being observed^31^.

The reason why AspH employs two- rather than three-protein derived metal ligands is unclear, but sequence comparisons suggest this is a highly-conserved feature (Supplementary Fig. 6). It also appears to be linked to other characteristic active site features involving 2OG and substrate binding, which may compensate for the lack of a third protein ligand. It is of interest that in the structure of AspH-TPR-Ox complexed with a 39-residue fragment of hFX EGF1 (AspH-TPR-Ox:hFX, Fig. 4) the Asp residue positioned for AspH-catalysed hydroxylation is observed in two conformations. One conformer is apparently positioned for stereoselective hydroxylation in a manner precedented for other 2OG oxygenases (Supplementary Fig. 21). By contrast, the Asp side chain carboxylate of the other conformer is positioned to coordinate with the active site metal (Fig. 4). Whilst the latter conformation may be involved in catalysis, it may be that within the endoplasmic reticulum, AspH substrates are involved in maintaining Fe(II) at its active site.

The unusual coordination chemistry of AspH may relate to an as yet undetermined physiological or disease role as it is the case for the 2OG dependent hypoxia-inducible factor (HIF) prolyl hydroxylases (PHDs), where it is proposed that the unusual kinetic properties of PHDs, i.e. their slow reaction with O_2_, both reflect their active site chemistry and roles as hypoxia sensors^31^. Given the unusual coordination chemistry of AspH, detailed kinetic and solution biophysical studies to investigate dioxygen binding and water ligand displacement are of considerable interest and are the subject of ongoing work.

We compared the AspH structures with the human HIF Asn-hydroxylase, factor inhibiting HIF (FIH, PDB: 1H2K)^66^, and a prokaryotic asparagine hydroxylase, AsnO (PDB: 2OG7)^67^ (Supplementary Fig. 21). Analysis of enzyme-substrate complex structures reveals similar substrate side chain orientations at the active sites of AsnO and FIH, consistent with knowledge that they produce the same (3*S*)-stereochemistry products^67,68^, which contrasts with the (3*R*)-product of AspH^1,36^. AsnO has an active site arginine (Arg305_AsnO_), which interacts with the Cα carboxylate of the asparagine substrate, similarly to the interaction of AspH Arg688 with the side chain of Asp103_hFX_ (Supplementary Fig. 20). However, Arg305_AsnO_ is located on β-strand VIII and AspH Arg688 is located on β-strand III of their respective DSBHs, possibly reflecting convergent evolution within the 2OG oxygenase superfamily. Interestingly, FIH also interacts with the side chain of its Asn (and Asp) substrates using an arginine, Arg238_FIH_, but one which is located on an insert linking DSBH β-strands βIV-V (Supplementary Fig. 21). Biochemical and structural studies on FIH metal binding carboxylate variants D201A and D201G reveal coordination by only two His residues and that the D201G variant is catalytically viable^69^. Comparison of these structures with AspH shows the main chain Cα of the carboxylate ligand positions are very similar, but that the hFX substrate residue side chain likely causes changes in metal geometry. It is probable that second sphere residues, including the substrate side chain, contribute to catalysis in the two-His metal binding 2OG oxygenases. 2OG dependent halogenases (SyrB2/CytC3/WelO5/AmbO5) also have two His metal binding ligands (His-Xxx-Ala…His motif), an arrangement enabling halide coordination to the active site metal^70^. We have not observed halogenase activity with AspH in the presence of chloride ions, implying other factors in addition to the number of metal ligands control substrate hydroxylation versus halogenation.

The ability to carry out assays with isolated AspH and knowledge of its active site and substrate binding mode provide a platform from which to develop small molecule AspH inhibitors. These will be useful in dissecting its biological functions and will help to enable new cancer treatments and the development of selective inhibitors for other human 2OG oxygenases, some of which are current medicinal chemistry targets^71^.

## Methods

### Recombinant protein production and purification

A pET-28a(+) vector encoding for N-terminally His_6_-tagged AspH_315–758_ was transformed into *E. coli* BL21 (DE3) cells. Cells were grown in 2TY media supplemented with kanamycin (0.05 mM; 30 mg/ml) at 37 °C with shaking (180 rpm). AspH production was induced by adding isopropyl ß-*D*-thiogalactopyranoside to a final concentration of 0.1 mM when the OD_600_ reached 1.2 at 18 °C. Cells were shaken for 16 h at 18 °C, then harvested by centrifugation (10,967 × *g*, 8 min, 4 °C); the resultant cell pellets were stored at –80 °C. The frozen cell pellets were resuspended in ice-cold (30 g/100 mL) 50 mM HEPES buffer (pH 7.5, 500 mM NaCl, 5 mM imidazole) containing EDTA-free protease inhibitor cocktail tablets (1 tablet/50 mL, Roche Diagnostics) and DNAse I (bovine pancreas, grade II, Roche Diagnostics). Cells were lysed by sonication on ice (8 × 30 s bursts; Sonics Vibra-Cell VCX500, amplitude: 60%) and the lysate was centrifuged (48,384 × *g*, 30 min, 4 °C). The supernatant containing AspH was purified at 4 °C using Ni(II)-affinity chromatography (HisTrap HP column, GE Healthcare) on an ÄKTA Pure machine (GE Healthcare) with a step elution gradient (to 50 mM HEPES, pH 7.5, 500 mM NaCl, 40 mM imidazole) and elution buffers (50 mM HEPES, pH 7.5, 500 mM NaCl, 500 mM imidazole; AspH typically eluted at around 150-200 mM imidazole). Eluted fractions containing AspH were pooled, concentrated using Amicon Ultra centrifugal filters (3082 × *g*, 4 °C), and further purified by size-exclusion chromatography using a HiLoad 26/60 Superdex 75 pg 300 mL column with a flow rate of 1 mL/min and 50 mM HEPES (pH 7.5, 500 mM NaCl) as elution buffer. The protein was subsequently concentrated and buffer exchanged into 50 mM HEPES, pH 7.5 for storage at −80 °C until further use. Purity of the proteins was assessed by SDS-PAGE and MS (Supplementary Fig. 5).

### AspH-substrates

A 39mer peptide based on the amino acid sequence of the first EGFD of hFX (hFX EGF1_39mer_, aa 86–124) was synthesized by solid phase peptide synthesis (SPPS) and purified by Peptide Synthetics (Peptide Protein Research Ltd, UK). Regioselective disulfide formation to yield exclusively (within our analytical limits) the canonical disulfide pattern (Cys1–3, 2–4, 5–6) was achieved using a sequence of orthogonal cysteine protection and selective cysteine oxidation^72^: However, no hydroxylation of this peptide was observed when exposed to His_6_-AspH_315–758_ and cofactors. Therefore, the 39mer peptide was resynthesized with the disulfide bridges being formed by thiol-oxidation in air-saturated buffer. This method has previously been applied to synthesize bovine FX^4^ and FIX^24^ peptides that were successfully hydroxylated by bovine AspH. Using this protocol, a mixture of the canonical (Cys1–3, 2–4, 5–6; C_39mer_) and non-canonical (Cys1–2, 3–4, 5–6; NC_39mer_) disulfide-isomers was obtained (Supplementary Fig. 12 and 13), which was used in all subsequent hydroxylation assays. Derivatives of hFX EGF1_39mer_ containing either canonical (C_26mer_) or non-canonical (NC_26mer_; NC-4Ser_39mer_) disulfides were synthesized as single disulfide isomers by GL Biochem (Shanghai) Ltd using orthogonal cysteine protection. The Ca(II)-binding EGFD-containing multi-domain AspH-substrates (hFIB1_cbEGFD32–33^49^, hFIB1_cbEGFD41–43^50^, hFIB1_TB4cbEGF23^51^, hNotch1_cbEGFD11–13^52^) were produced recombinantly in *E. coli* and purified as reported in the cited literature.

### Design and synthesis of cyclic peptides

Based on the AspH-TPR-Ox:hFX crystal structure, stable cyclic peptides comprising the core ring residues (aa 101–110) of the hFX EGF1_39mer_-substrate with a thioether replacing the Cys3–4 disulfide were synthesized. The *D*-stereochemistry of the N-terminal amino acid (aa 101) aligns the peptide side chain with the main chain of the original substrate.

Commercial Fmoc-protected amino acids (AGTC Bioproducts; Alfa Aesar; CSBio; Iris Biotech; Novabiochem; Sigma-Aldrich; TCI) were used as received. SPPS was performed using a CSBio CS336X automated peptide synthesizer following standard Fmoc-strategy: linear peptides were synthesized from the C- to N-termini on a 0.1 mmol scale using a Rink amide linker using *N*,*N*-diisopropylcarbodiimide/1-hydroxybenzotriazole for coupling.

To obtain cyclic peptides, the *N*-terminal Fmoc-protecting group was cleaved on the resin, and the resin suspended in DMF (4 mL) containing *N*-chloroacetylsuccinimide^73^ (150 mg). The mixture was gently shaken for 3 h, filtered, and the dried resin treated with a solution of trifluoroacetic acid, triisopropylsilane, and water (4 mL; 95/2.5/2.5). After 3 h, the mixture was filtered and the solution diluted with 45 mL cold diethyl ether. The suspension was centrifuged (4000 rpm, 10 min, 4 °C), decanted, and then taken up in 1.5 mL aqueous triethylammonium acetate buffer (1 M, pH 8.5, pH readjusted with trimethylamine) and heated for 10 min at 100 °C in a microwave reactor (Biotage Initiator). Directly afterwards, the crude cyclic peptides were purified by semi-preparative HPLC (DionexTM UltiMate^®^ 3000, Thermo Scientific) using a reverse phase column (Grace Vydac^®^ 218TP101522) and a gradient of acetonitrile in milliQ water (each containing 0.1%_v/v_ trifluoroacetic acid) specified in Supplementary Fig. 14.

### AspH activity assays

All reagents were obtained from commercial sources (Alfa Aesar; Sigma-Aldrich; TCI). For all turn-over experiments, His_6_-tagged AspH-constructs were used, as no effect upon cleavage of the His_6_-tag was observed in initial activity assays. Hydroxylation assays were initialised by mixing a solution containing AspH (10 μM), L-ascorbic acid (400 μM), and (NH_4_)_2_Fe(SO_4_)_2_·7H_2_O (50 μM) in the appropriate buffer with a solution containing substrate (100 μM), disodium 2-oxoglutarate (300 μM), and, if desired, additional reagents (CaCl_2_, EGTA) in the appropriate buffer. The buffers used comprised: Standard, non-redox, Buffer (50 mM HEPES pH 7.5, 150 mM NaCl); Redox Buffer (50 mM Tris, pH 8.5, 3.0 mM reduced L-glutathione, 0.3 mM oxidized L-glutathione, 150 mM NaCl); Refolding Buffer (50 mM Tris, pH 8.5, 3.0 mM L-cysteine, 0.3 mM L-cystine, 10 mM CaCl_2_). Reactions were performed at 37 °C for 60 min unless otherwise noted, and then quenched by the addition of an equal volume of 1%_v/v_ aqueous formic acid. All assays were performed in triplicate alongside an additional no-enzyme control (His_6_-AspH substituted by buffer).

The hydroxylase activity of AspH was assayed by MALDI-ToF MS in the positive ion reflectron mode (Bruker Daltonics Ultraflex I machine with 32–60% laser energy; Waters Micromass MALDI micro MX with flight tube voltage: 12 kV, reflectron voltage: 5.2 kV, laser fire rate: 10 Hz, pulse voltage: 1950 V, detector voltage: 2750 V): the relative quantities of substrate and hydroxylated product were analysed with respect to no-enzyme controls. A 1:4 volume ratio of sample to MALDI-matrix was used for sample preparation (20 mg/mL 2,5-dihydroxybenzoic acid, 10 mg/mL α-cyano-4-hydroxycinnamic acid or 10 mg/mL sinapinic acid in aqueous 50% CH_3_CN containing 0.1%_v/v_  trifluoroacetic acid). For the disulfide containing substrates a matrix composed of a 10:1 mixture of 10 mg/mL α-cyano-4-hydroxycinnamic acid:10 mg/mL 2-(4′-hydroxybenzeneazo)benzoic acid in aqueous 50%_v/v_ CH_3_CN containing 0.1%_v/v_ trifluoroacetic acid was used. Mass spectra were analysed using MassLynx software (Version 4.1). Apart from the AspH-substrate peaks, peaks corresponding to the desired AspH-substrate mass reduced by 18 Da are frequently observed in the MALDI-MS spectra; these peaks result from the ionization induced liberation of H_2_O upon intramolecular cyclization of aspartates to form aspartyl succinimides (−18 Da)^74^ and are taken into account when integrating the peaks to determine conversions.

### Crystallography

Crystals of *N*-terminally His_6_-tagged AspH_562–758_ were grown by the vapour diffusion method at 20 °C in 150 nL sitting drops. The drops were prepared by mixing 100 nL of protein solution (9.5 mg/mL containing 1 mM *N*-oxalylglycine (NOG) and 10 mM NiSO_4_) and 50 nL of precipitant (50 mM disodium malate, 30%_v/v_ PEG-3350, 30% dextran sulfate sodium salt) with a protein to well ratio of 1:2. Crystals were cryo-cooled in liquid N_2_ with 25% glycerol as cryoprotectant and data were collected at 100 K using synchroton radiation at the Diamond Light Source (DLS).

For the remaining structures, high-throughput crystallization screens were set up using a Phoenix RE liquid dispensing robot (Art Robbins Instruments) on 96-well, 3-subwell low profile Intelliplates (Art Robbins Instruments) using Hampton Research Molecular Dimensions crystallization screens (JCSG plus, PACT, INDEX, PEG/Ion, Structure, Salt, MIDAS) or optimization screens. *N*-Terminally His_6_-tagged AspH_315–758_ (18 mg/mL in 50 mM HEPES buffer, pH 7.5) was mixed with 1 mM MnCl_2_ (pH 7.5), 2 mM *N*-oxalylglycine (pH 7.5), and, when appropriate, an AspH-substrate (see Supplementary Table 1 for details). Crystals were grown by the vapour diffusion method at 4 °C in 200 or 300 nL sitting drops using 2:1, 1:1 or 1:2 sample:well solution ratios. Crystals were cryoprotected using mother liquor supplemented with 25%_v/v_ glycerol before cryo-cooling in liquid N_2_. Data were collected at 100 K at DLS. Data were indexed, integrated, and scaled using HKL-3000^75^, XDS^76^, SCALA^77^, Xia2^78^ or CrystalClear (Rigaku). Detailed crystallization conditions for all crystal structures and a summary of data collection parameters are given in Supplementary Table 1.

### Structure solution and refinements

The structure of AspH-Ox was determined by single isomorphous replacement with anomalous scattering (SIRAS). Briefly, AspH crystals were derivatised by incubating in a reservoir solution supplemented with 10 mM K_2_PtCl_4_ for 5 h. Crystals were transferred to and backsoaked for several seconds in a reservoir solution supplemented with 25% ethylene glycol instead of Pt(II) and crystals were immediately cryo-cooled in liquid N_2_.

The AspH-Ox (native and Pt-derivatised) datasets were integrated (XDS^76^) and subsequently scaled (SCALA). SHELXD^79^ was used to locate three platinum positions in the Pt-dataset. Heavy atom positions were refined and initial phases calculated with SHARP^80^ using the SIRAS method. The final electron density map after phase extension and solvent flattening with SOLOMON^81^ was of excellent quality and automated model building with ARP/wARP^82^ resulted in a 95% complete model. Refinement with PHENIX^83^ and several rounds of manual rebuilding in COOT^84^ resulted in a model with a final R_factor_/R_free_ of 15.3/18.6%, respectively.

The AspH-TPR-Ox structure was determined by molecular replacement (MR) using the AutoMR (PHASER^85^) subroutine in PHENIX. The coordinates of the AspH oxygenase domain (His_6_-tagged AspH_562–758_ as a search model, PDB: 5APA) were first used to successfully identify a rotation and translation solution for the oxygenase domain. Initial attempts to find a rotation and translation solution for the TPR using the C-terminal TPR domain of peroxisomal targeting signal 1 receptor (PEX5)^86^ were complicated by the repeating nature of the TPR fold. In order to overcome problems associated with the repeating fold the search model was trimmed to include three sequential repeats (PEX5 Gln300-Tyr395, PDB: 1FCH). The 3 C-terminal TPR repeats of AspH were identified in a search after the oxygenase domain was fixed. This allowed manual fitting of 3 further TPR repeats to the N-terminus of AspH. Rigid body refinement of the three separate fragments followed by simulated annealing refinement (both cartesian and torsion) led to a model with R_free_ 42.15. Iterative rounds of model building and fitting in COOT^84^ and refinement in PHENIX^83^ were performed until the decreasing R and R_free_ no longer converged and a final model was obtained. Due to domain movements between different crystal forms and complexes, all other structures were determined by MR using PHASER with the oxygenase and TPR domains searched for independently. The oxygenase domain was always searched first followed by the TPR domain. For data from crystals grown in the presence of substrate, up to 18 residues of bound substrate were modelled. Refinement statistics are given in Supplementary Table 2. Representative electron density (with contour level and map types defined in legends) is shown in Figs. 2 and 3 and in Supplementary Figs. 4, 7, 16–18.

### Reporting summary

Further information on research design is available in the Nature Research Reporting Summary linked to this article.

## Supplementary information

Supplementary Information

Reporting Summary

## Data Availability

Crystal structure data for *N*-terminal His_6_-tagged AspH_562–758_ and *N*-terminal His_6_-tagged AspH_315–758_ (apo and in complex with different AspH-substrates) are deposited in the protein databank with PDB accession codes: 5APA (AspH-Ox), 5JZA (AspH-TPR-Ox), 5JZ6 (AspH-TPR-Ox:malate), 5JZ8 (AspH-TPR-Ox:hFX), 5JQY (AspH-TPR-Ox:NC-Ser_39mer_), 5JZU (AspH-TPR-Ox:NC_26mer_), 6RK9 (AspH-TPR-Ox:CP_101–119_). Other data are available from the corresponding author upon reasonable request.
