# Supplementary material for: Aspartate/asparagine-β-hydroxylase crystal structures reveal an unexpected epidermal growth factor-like domain substrate disulfide pattern
Source: Nat Commun. 2019 Oct 28;10:4910. doi: 10.1038/s41467-019-12711-7 (PMC6817910; doi:10.1038/s41467-019-12711-7)
Supplement: Supplementary file 1 — Supplementary Information [file 41467_2019_12711_MOESM1_ESM.pdf]

Aspartate/Asparagine-β-Hydroxylase Crystal Structures Reveal an Unexpected Epidermal Growth Factor-like Domain Substrate Disulfide Pattern

Inga Pfeffer, Lennart Brewitz, Tobias Krojer, Sacha A. Jensen, Grazyna T. Kochan, Nadia J. Kershaw, Kirsty S. Hewitson, Luke A. McNeill, Holger Kramer, Martin Münzel, Richard J. Hopkinson, Udo Oppermann, Penny A. Handford, Michael A. McDonough, and Christopher J. Schofield*

*christopher.schofield@chem.ox.ac.uk

Supplementary Figure 1. Sequence alignment of reported isoforms of human AspH. By 2019, 12 human AspH isoforms (A-L) are present in the National Center for Biotechnology Information (NCBI) database[1] (gene ID: 444). Only AspH_A (residues 562-758) and AspH_F contain the catalytic oxygenase domain. AspH isoforms B (junctate), C (humbug), and D (junctin) and E (junctin isoform 1) have biological functions apparently not involving Asp/Asn-β-hydroxylation. The sequence numbering of the AspH constructs used in this study is based on the longest full-length sequence of human AspH isoform A (AspH_A); the oxygenase domain (residues 562-758) and the tetratricopeptide repeat domain (residues 315-561) were predicted in the National Center for Biotechnology Information (NCBI) database[1]. Sequences were obtained from the NCBI database[1] and aligned using GeneDoc 2.7[2]; black shading indicates high sequence conservation; metal binding residues are indicated in red; 2OG binding residues are indicated in blue.

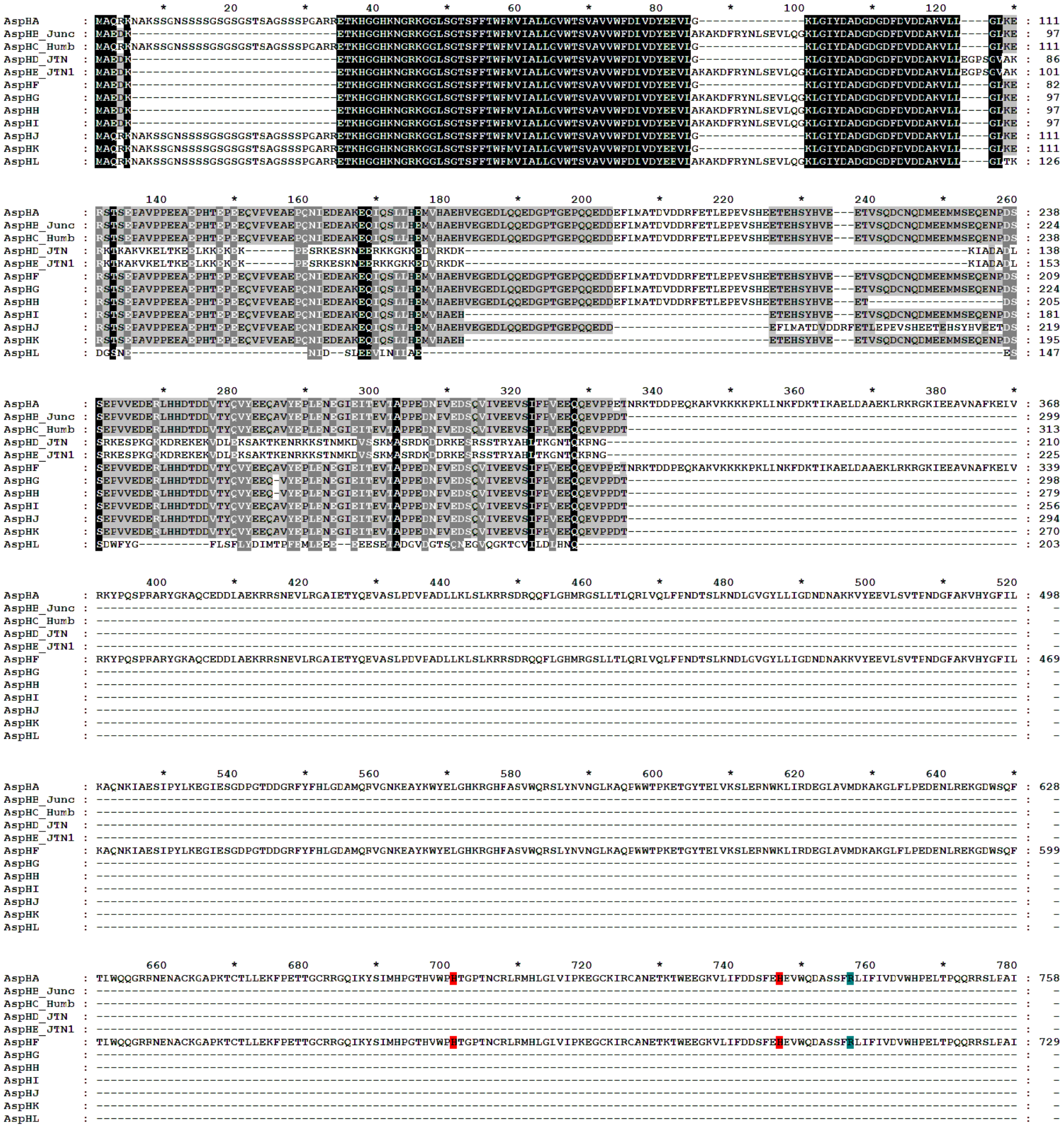

Supplementary Figure 2 (continues on the following page). N-Terminally His$_6$-tagged AspH$_{562-758}$ (AspH-Ox) was obtained in highly purified form as analyzed by sodium dodecyl sulfate polyacrylamide gel electrophoresis (SDS-PAGE) with Coomassie staining and electrospray ionization mass spectrometry (ESI-MS).

Human His$_6$-tagged AspH$_{562-758}$ was produced and purified as follows: DNA encoding for human AspH$_{562-758}$ with a N-terminal His$_6$-tag was cloned into a pNIC28-Bsa4 plasmid. After transformation of the plasmid into *E. coli* BL21 (DE3)-R3-pRARE2 cells, expression was performed at 37 °C in TB medium, supplemented with 20 g/L glycerol, 50 µg/mL kanamycin, and 34 µg/mL chloramphenicol. AspH-production was induced at an OD$_{600}$ of ≈1.5 at 18 °C by adding isopropyl ß-D-thiogalactopyranoside (1 M) to a final concentration of 0.1 mM. Cultures were grown for a further 18 hours. Cells were centrifuged at 5000 rpm for 10 min and the resulting cell pellet stored at -80 °C.

The cell pellets were suspended in 50 mM HEPES (pH = 7.5, 500 mM NaCl, 5 mM imidazole, 5% glycerol) containing EDTA-free protease inhibitor cocktail tablets (1 tablet/50 mL, Roche Diagnostics) and lysed by high pressure homogenization. Following cell lysis, the insoluble cell debris was removed by centrifugation (36000 x g, 1 h, 4 °C). His$_6$-tagged AspH$_{562-758}$ was purified using HisTrap CrudeFF 5ml resin (GE Healthcare) equilibrated with binding buffer (50 mM HEPES, pH 7.5, 500 mM NaCl, 5% glycerol, 20 mM imidazole, 1 mM PMSF, 0.5 mM TCEP). After a wash step with binding buffer supplemented with 40mM imidazole, protein was eluted with binding buffer supplemented with 250mM imidazole. The fractions containing His$_6$-AspH$_{562-758}$ were pooled, concentrated, and purified further by size-exclusion chromatography using a Superdex S200 column, HiPrep 16/60 (Amersham) equilibrated with 10 mM HEPES, pH 7.5; 500 mM NaCl; 5% glycerol; 0.5 mM TCEP. ASPH containing fractions were pooled and diluted with IEX buffer A (25 mM Tris, pH 7.0; 25 mM NaCl) to a NaCl concentration of 25 mM. The protein was loaded onto a HiTrap SP Sepharose column, and eluted with a NaCl gradient (0-25% buffer B). Fractions containing protein were analyzed by SDS-PAGE. ASPH containing fractions were pooled, concentrated and stored at -80°C.

(a) 10-12% SDS-PAGE analysis of His$_6$-AspH$_{562-758}$ after Ni(II)-affinity chromatography (HisTrap column); pooled fractions are boxed in red; **(b)** 10-12% SDS-PAGE gel analysis of His$_6$-AspH$_{562-758}$ after subsequent size-exclusion chromatography (Superdex 75); pooled fractions are boxed in red.

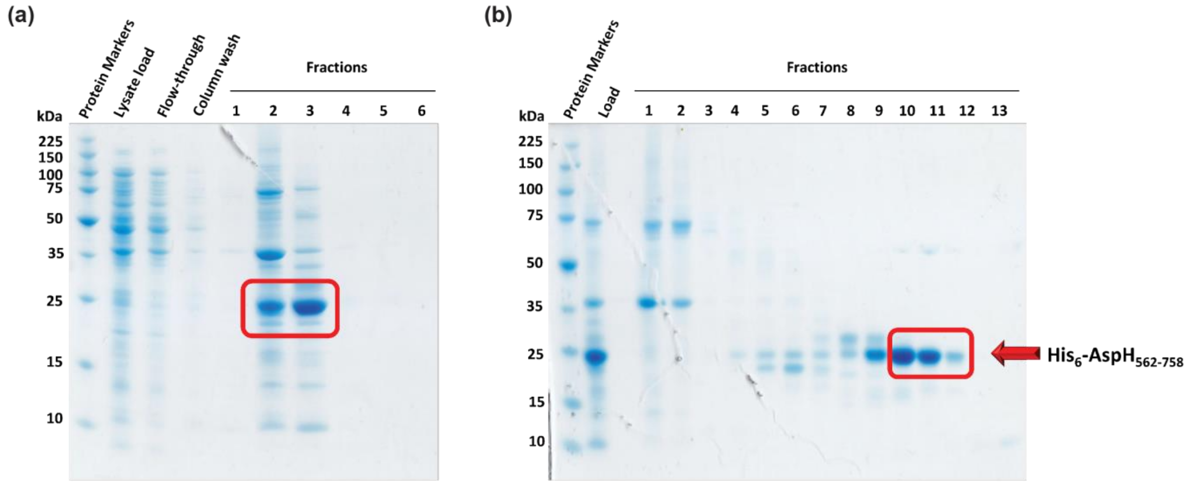

(c) Combined ion series (ESI-MS) of the purified His$_6$-AspH$_{562-758}$-construct after size-exclusion chromatography; **(d)** Deconvoluted mass spectrum of the purified His$_6$-AspH$_{562-758}$-construct after size-exclusion chromatography shows the expected calculated mass of His$_6$-AspH$_{562-758}$ (25.601 kDa) and an additional species (+ 178 Da) corresponding to His$_6$-AspH$_{562-758}$ containing a gluconoylated His$_6$-tag, a common covalent modification for recombinant N-terminal His$_6$-tagged proteins produced in *E. coli*[3,4], which does not impact substantially (if at all) on AspH-catalysis.

(c)

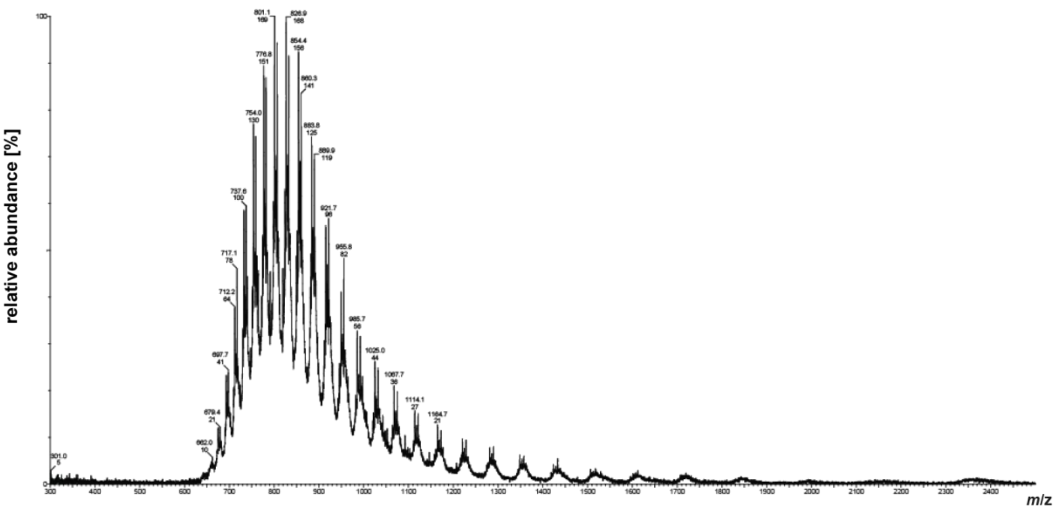

(d)

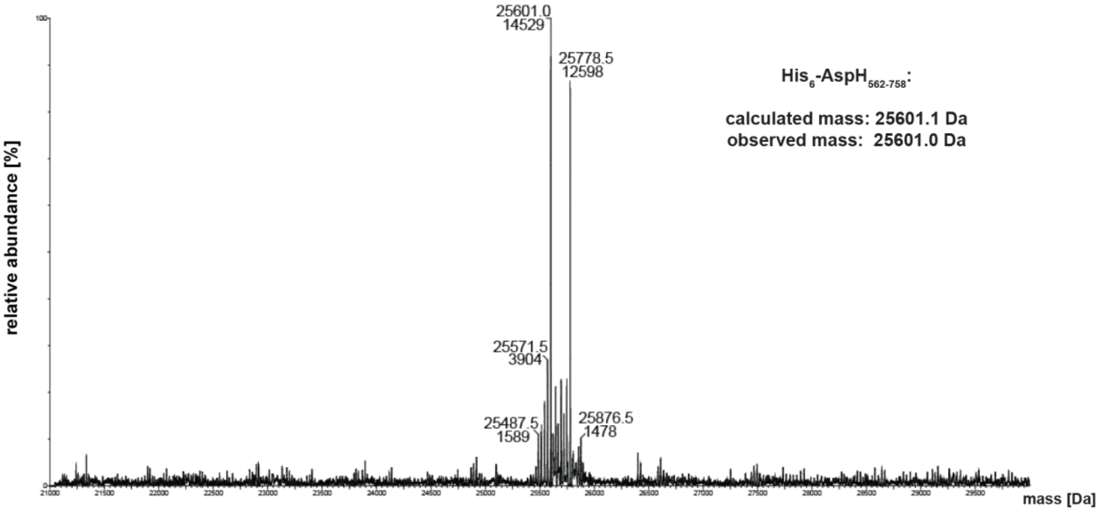

Supplementary Figure 3. [1-^{14}C]-2-Oxoglutarate (2OG) turnover assay indicates His$_6$-AspH$_{562\text{-}758}$-catalyzed turnover of 2OG is uncoupled from Asp-β-oxidation of EGF1 of human Coagulation Factor X (hFX EGF1$_{39mer}$). (a) Schematic depiction of the radiochemical [1-^{14}C]-2OG turnover assay: hyamine hydroxide functions as $^{14}CO_2$-trapping agent,[5] the β-emission of hyamine [^{14}C]-bicarbonate was measured by liquid scintillation counting; **(b)** Little turnover is observed in the absence of His$_6$-AspH$_{562\text{-}758}$, whereas the addition of His$_6$-AspH$_{562\text{-}758}$ results in an approximately 10 fold increase of hyamine-trapped [^{14}C]-bicarbonate formation; the presence of hFX-EGF1$_{39mer}$-substrate (aa 86-124) does not influence 2OG oxidation (p = 0.368). Data (background substracted) is shown as the mean of triplicates with error bars indicating the standard derivation (n = 3; mean ± SD), individual data points are shown as black circles; **(c)** The ^{1}H NMR spectrum of 2OG exposed to His$_6$-AspH$_{562\text{-}758}$ in the absence of hFX-EGF1$_{39mer}$-substrate shows the formation of succinate. Note, the extent of 2OG-turnover which is uncoupled from AspH-substrate hydroxylation is small (see Supplementary Figures 14 and 19 for comparison). The assay was initiated by mixing a solution containing His$_6$-AspH$_{562\text{-}758}$ (10 µM final concentration) with a solution containing L-ascorbic acid (1 mM final concentration), $(NH_4)_2Fe(SO_4)_2\cdot 7H_2O$ (100 µM final concentration), and disodium 2-oxoglutarate (2 mM final concentration) in 50 mM [$^2H_{11}$]-Tris-^{2}HCL buffer (pH = 7.1) and incubated at 25 °C for 2 h (75 µL total volume). The mixture was analysed using a Bruker Avance III 700 MHz NMR spectrometer; **(d)** Oxidation of the hFX-EGF1$_{39mer}$-substrate (mixture of disulfide isomers) was not observed in the presence of His$_6$-AspH$_{562\text{-}758}$; the end-point turnover assay was performed as outlined in the Methods Section. The light orange graph represents a parallel control in which AspH was replaced by buffer (no-enzyme control).

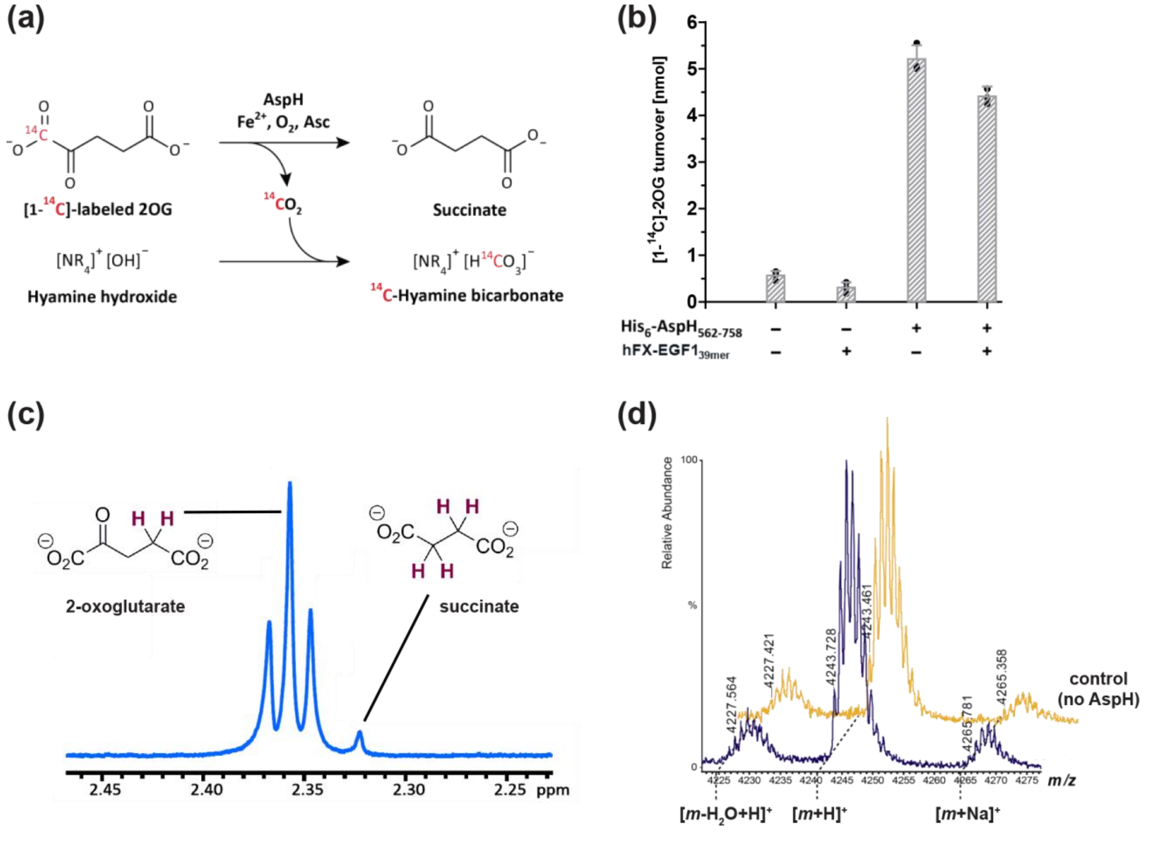

Supplementary Figure 4 (continues on the following page). View from crystal structure of AspH-Ox (PDB entry: 5APA). Color code: grey: His_6-$AspH_{315\text{-}758}$; cyan: carbon-backbone of L-malate; bronze: Ni(II); red: oxygen; blue: nitrogen. w: water.

(a) Overview of the AspH-Ox crystal structure: L-malate replaces 2OG in the 2OG binding pocket of the AspH-active site. Roman numerals label the 8 β-strands forming the 8 β-stranded double-stranded beta-helix (DSBH) fold.

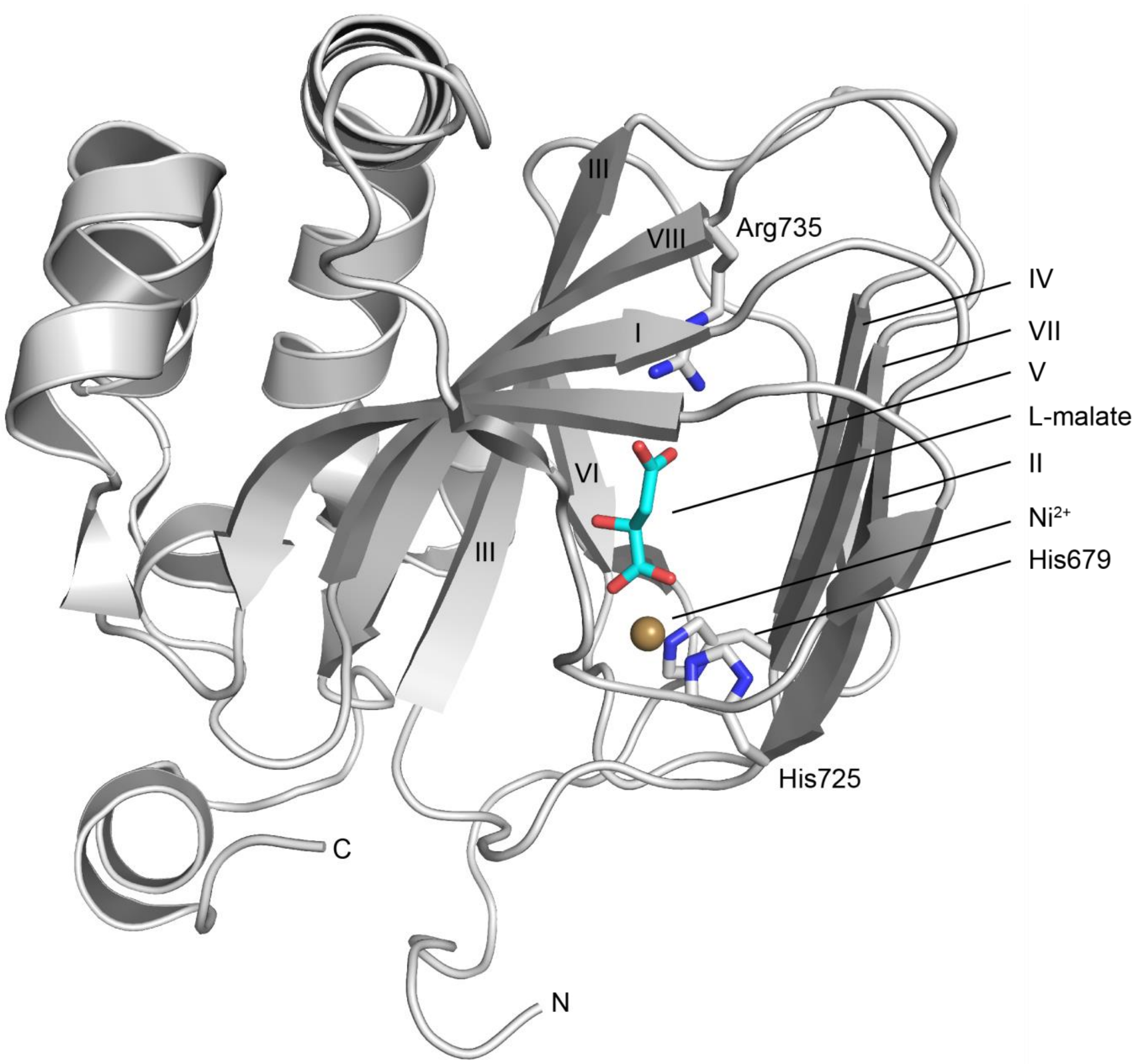

(b) Close-ups of the AspH-active site: **(1)** Arg735 is positioned to form a salt bridge with the 'distal' (C-4) carboxylate of L-malate (2.8 Å); Ser668 is positioned to interact with one oxygen lone pair via a hydrogen bond (2.7 Å). His690 forms a hydrogen bond with the hydroxyl group of L-malate (2.7 Å). Ni(II) binds His_6-$AspH_{315-758}$ by coordinating to His679 and His725 (2.1 Å), it also coordinates two water molecules (2.2 Å) and both oxygen atoms of the C-1 carboxylate of L-malate (2.2 Å); **(2)** Representative OMIT electron density map (mF_o-DF_c) contoured to 3σ around L-malate and Ni(II) from the AspH-TPR-Ox:malate crystal structure.

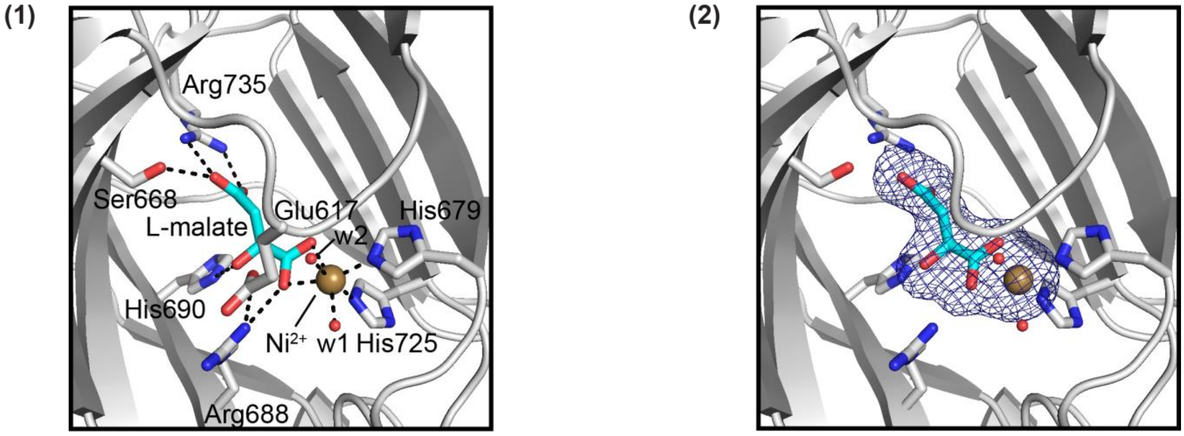

(c) Stereoview of the AspH-Ox crystal structure.

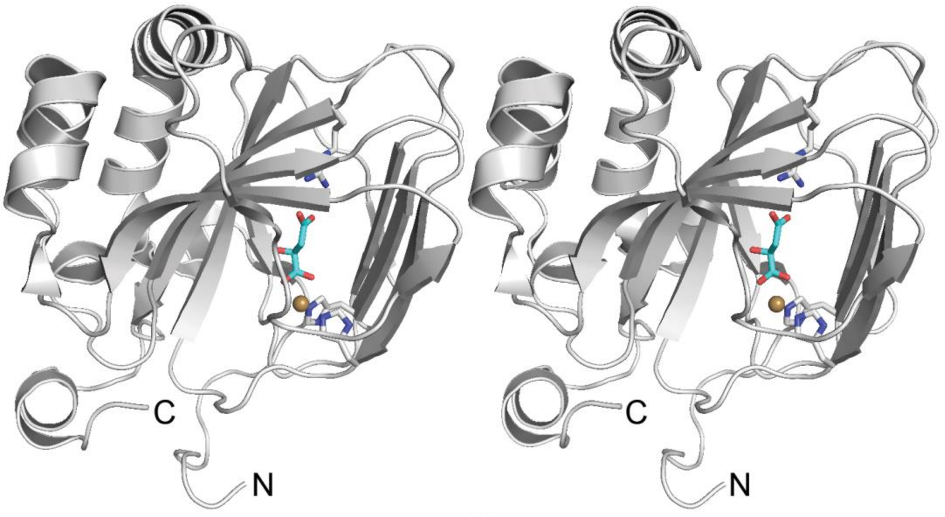

Supplementary Figure 5. N-Terminally His$_6$-tagged AspH$_{315-758}$ (AspH-TRP-Ox) was obtained in highly purified form as analyzed by SDS-PAGE with Coomassie staining and electrospray ionization mass spectrometry (ESI-MS). (a) 10-12% SDS-PAGE analysis of His$_6$-AspH$_{315-758}$ after Ni(II)-affinity chromatography (HisTrap column); pooled fractions are boxed in red; **(b)** 10-12% SDS-PAGE analysis of His$_6$-AspH$_{315-758}$ after subsequent size-exclusion chromatography (Superdex 75); pooled fractions are boxed in red; **(c)** Combined ion series (ESI-MS) of the purified His$_6$-AspH$_{315-758}$-construct after size-exclusion chromatography; **(d)** Deconvoluted mass spectrum of the purified His$_6$-AspH$_{315-758}$-construct after size-exclusion chromatography shows the expected calculated mass of His$_6$-AspH$_{315-758}$ (54.519 kDa) and an additional species (+ 178 Da) corresponding to His$_6$-AspH$_{315-758}$ containing a gluconoylated His$_6$-tag, a common covalent modification for recombinant N-terminal His$_6$-tagged proteins produced in *E. coli*[3,4], which does not impact substantially (if at all) on AspH-catalysis.

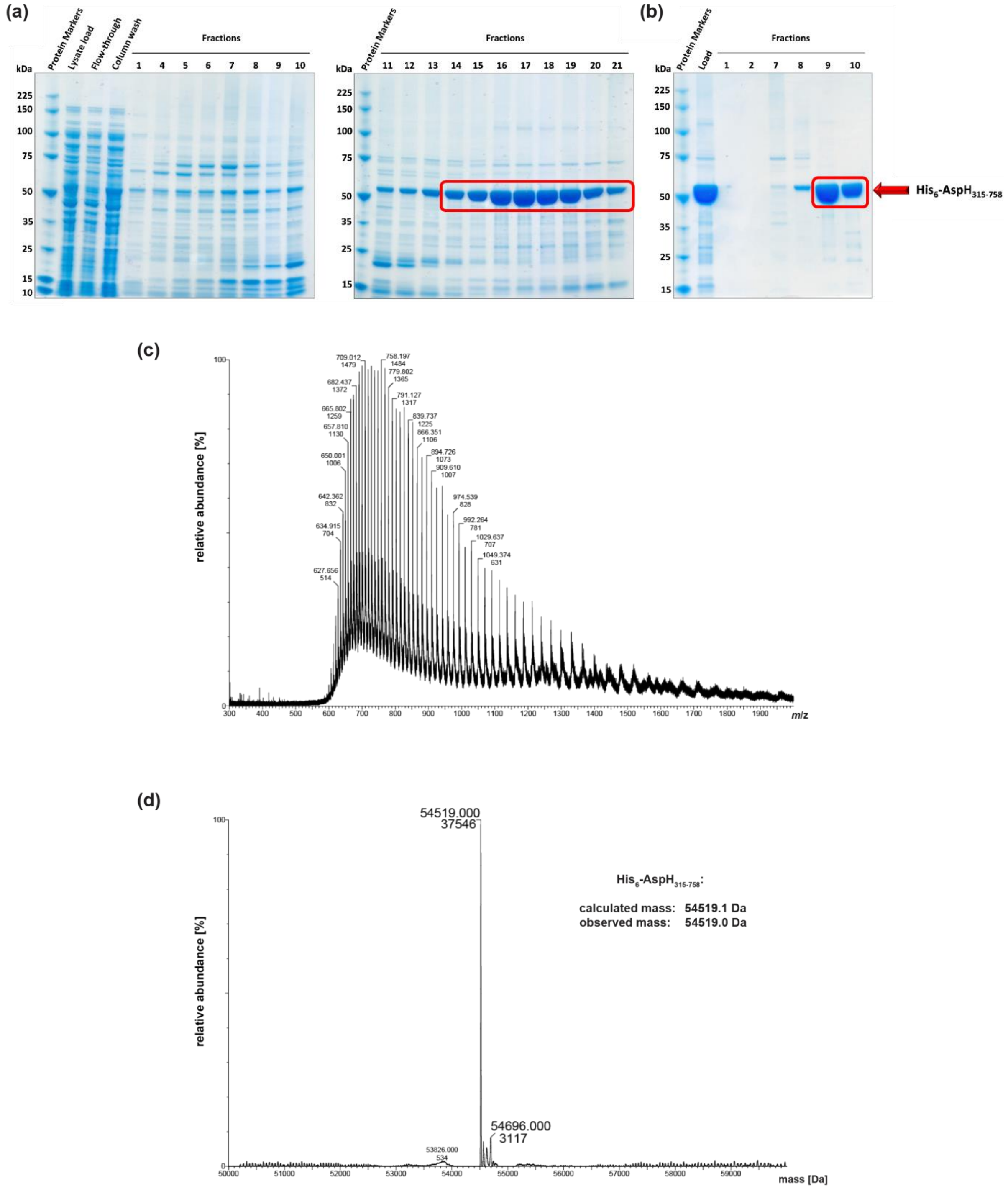

Supplementary Figure 6. Multi-species sequence alignment of AspH. The sequence of human $AspH_{315-758}$ was aligned to AspH sequences from different organisms. Yellow or red shading indicates sequence similarity or identity, respectively. The human $AspH_{315-758}$ secondary structure was generated using ESPript 3.0[6] and is shown above the sequences: Helices as helix, strands are arrows with coloring as in Figure 2 (TPR domain secondary structure in red, oxygenase domain in gold with core DSBH strands in green – major sheet in dark green, minor sheet in light green). Metal binding residues indicated with a red star; 2OG binding residues indicated with a blue star; The acidic loop is indicated by an orange oval; The hinge region is indicated by violet hexagons. National Center for Biotechnology Information database[1] or Uniprot database reference sequences were used: ***Hs***: *Homo sapiens* (human, NP_004309.2); ***Bt***: *Bos taurus* (cow, NP_777182.1); ***Gg***: *Gallus gallus* (chicken, A0A1D5NWS0 entry 16 as of 16.01.2019); ***Dr***: *Danio rerio* (zebrafish, XP_021333678.1); ***Dm***: *Drosophila melanogaster* (fly, Q9GQ82); ***Aa***: *Aedes albopictus* (mosquito, KXJ81530.1); ***Ta***: *Trichoplax adhaerens* (Placozoan, simplest animal, XP_002117061.1); ***Aq***: *Amphimedon queenslandica* (sponge, XP_019853498.1). AspH is apparently conserved in a wide variety of species, including early animals such as *Trichoplax adhaerens* and sponges, supporting an important evolutionary conserved biological function of AspH. Note; the conserved hinge region enables an induced fit mechanism upon AspH substrate binding and residues involved in metal and 2OG binding are highly conserved.

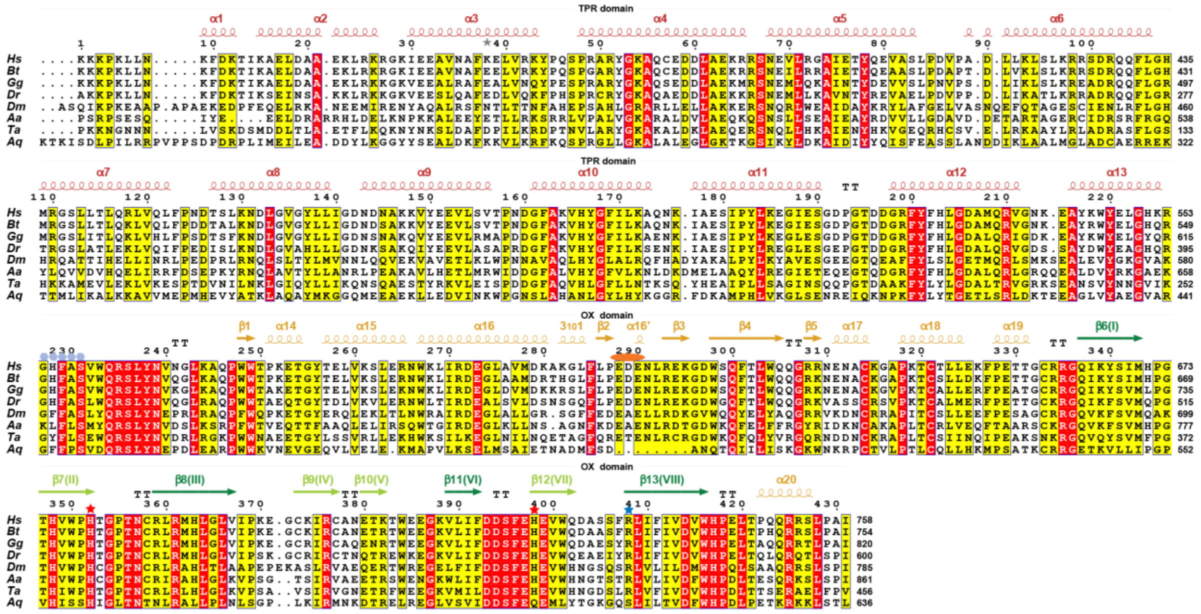

Supplementary Figure 7 (continues on the following page). Crystal structure of AspH-TPR-Ox:malate (PDB entry: 5JZ6). Color code: grey: His_6-$AspH_{315\text{-}758}$; cyan: carbon-backbone of L-malate; violet: Mn(II); red: oxygen; blue: nitrogen. w: water.

(a) L-Malate replaces 2OG in the 2OG binding pocket in the AspH-TPR-Ox:malate crystal structure: **(1)** Overview of the AspH-TPR-Ox:malate crystal structure; **(2)** Close-up of the AspH-active site: Arg735 forms a salt bridge with the distal (C-4) carboxylate of L-malate (3.0 Å) while Ser668 interacts with one oxygen lone pair through a hydrogen bond (2.7 Å). His690 forms hydrogen bonds with Trp711 (3.1 Å) and the hydroxyl group of L-malate (2.8 Å). Mn(II) is bound to His679 (2.3 Å) and His725 (2.2 Å) of His_6-$AspH_{315\text{-}758}$ and coordinates two water molecules (1.9 and 2.2 Å) as well as both oxygen atoms of the C-1 carboxylate of L-malate (2.2 Å); **(3)** Representative OMIT electron density map ($mF_o - DF_c$) contoured to 3σ around L-malate and Mn(II) from the AspH-TPR-Ox:malate crystal structure.

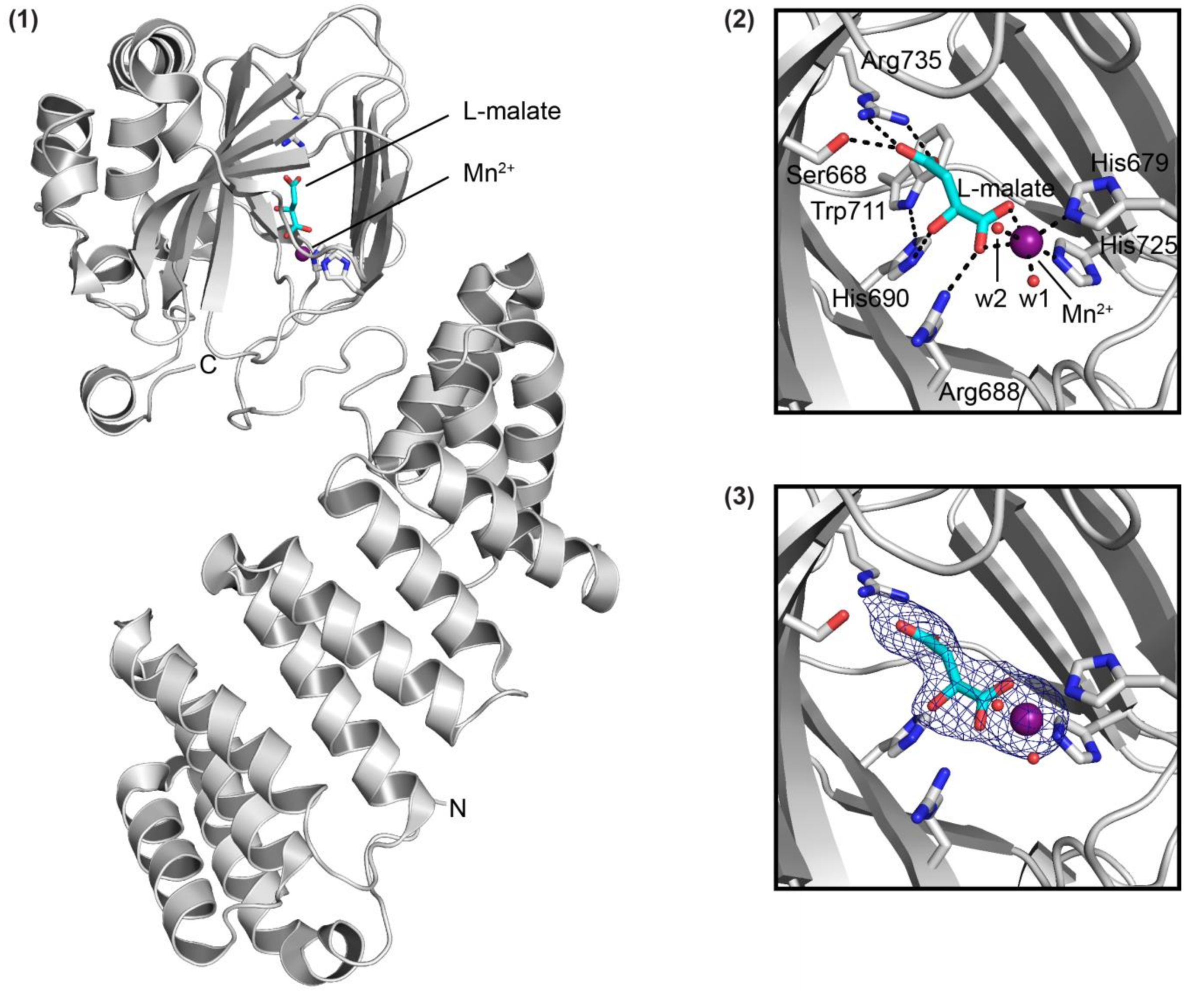

(b) Stereoview from the AspH-TPR-Ox:malate crystal structure.

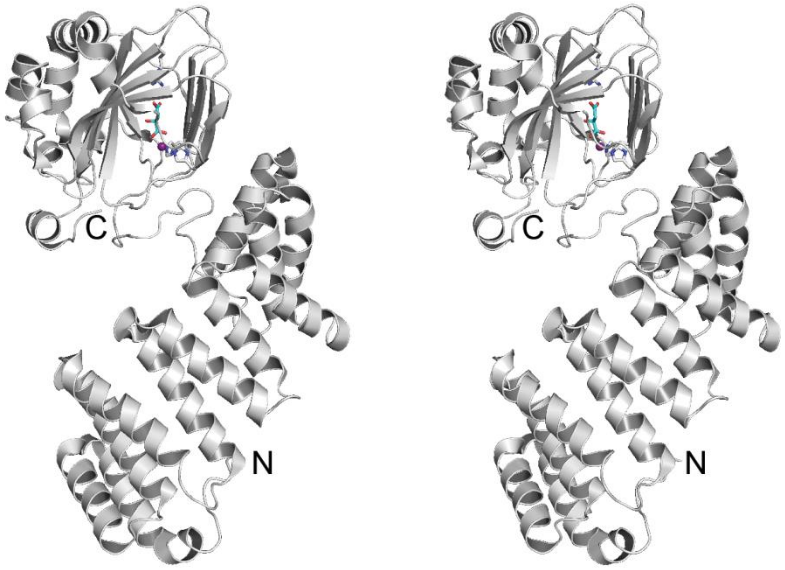

(c) Superimposition of the oxygenase domains of the AspH-TPR-Ox:malate (grey) and AspH-Ox (metallic green, PDB entry: 5APA) crystal structures. The 2 structures are highly similar; the binding mode of L-malate in the active site is apparently identical. The most pronounced structural differences are observed for the N-terminal region of the oxygenase domain, the flexible acidic loop region connecting β-strands 2 and 3 (Pro614 to Arg620, see topology diagram in Figure 2 for details) and the amino acid residues forming 3_{10}-helix 1 (Met605 to Leu613, see topology diagram in Figure 2 for details).

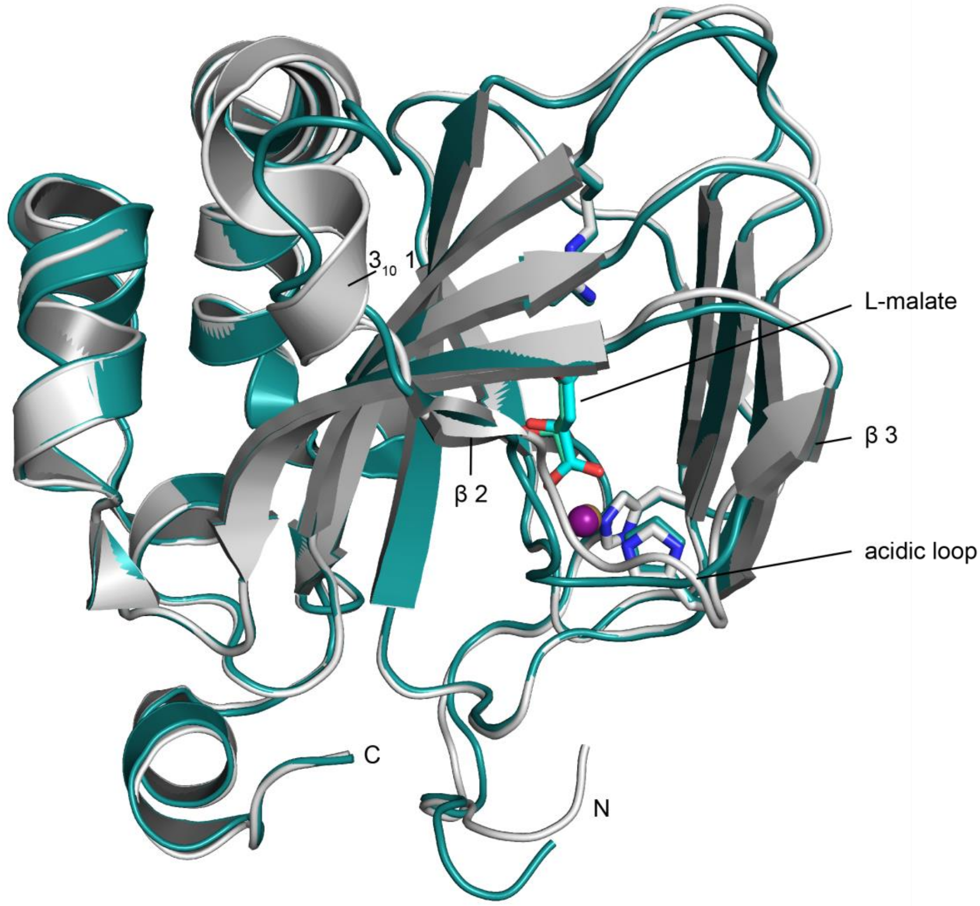

Supplementary Figure 8. Superimposition of the AspH-TPR-Ox:malate (PDB entry: 5JZ6) and AspH-TPR-Ox (PDB entry: 5JZA) crystal structures. Color code: cyan: carbon-backbone of L-malate; yellow: carbon-backbone of *N*-oxalylglycine; violet: Mn(II); red: oxygen; blue: nitrogen.

(a) The AspH-oxygenase domains of AspH-TPR-Ox:malate (grey cartoon) and AspH-TPR-Ox (limon cartoon) structures overlap well, while the AspH-TPR domains show a slight deviation of the N-terminal repeats.

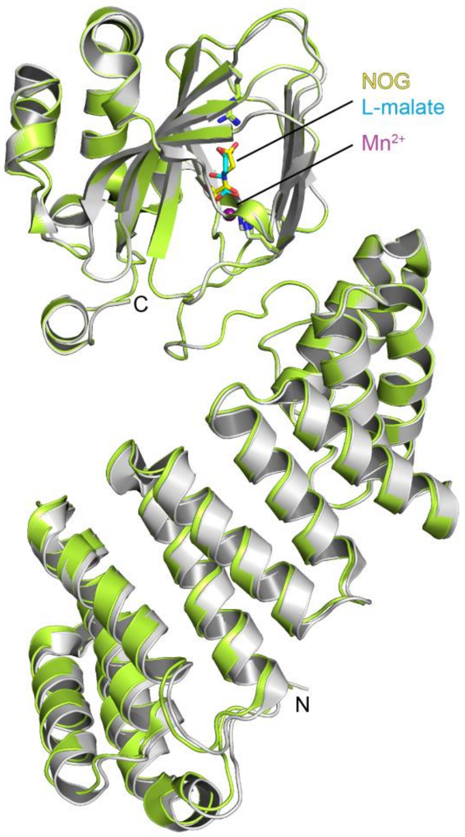

(b) Superimposition of the AspH-TPR-Ox (limon sticks) and AspH-TPR-Ox:malate (grey sticks) active sites shows similar spatial arrangement of important amino acid side chains. However, a different binding mode of the ligand replacing 2OG in the active sites is observed: While L-malate (cyan sticks) coordinates the metal by its two C-1 oxygen atoms and interacts with His690 via its C-2 hydroxyl group, *N*-oxalylglycine (yellow sticks) coordinates to the metal (purple sphere) with one C-1 oxygen atom and the C-2 carbonyl group. 2-OG binding to the AspH-active site might be more accurately reflected by *N*-oxalylglycine with which it is near isosteric.

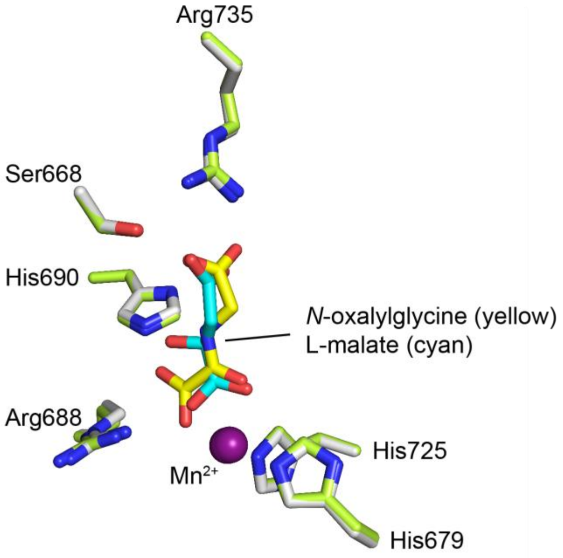

Supplementary Figure 9. Substrate binding groove and stereoview of the AspH-TPR-Ox:hFX crystal structure (PDB entry: 5JZ8). Color code: magenta: carbon-backbone of the NC_{39mer} peptide; yellow: carbon-backbone of NOG; violet: Mn(II); red: oxygen; blue: nitrogen; pale yellow: sulfur.

(a) Three different orientations of surface representations of the AspH-TPR-Ox:hFX crystal structure (two 90° rotations around the y-axis of His_6-$AspH_{315-758}$) indicate the presence of a substrate binding groove (the structure of His_6-$AspH_{315-758}$ is shown in green); **(b)** Stereoview of the AspH-TPR-Ox:hFX crystal structure; His_6-$AspH_{315-758}$ is in grey.

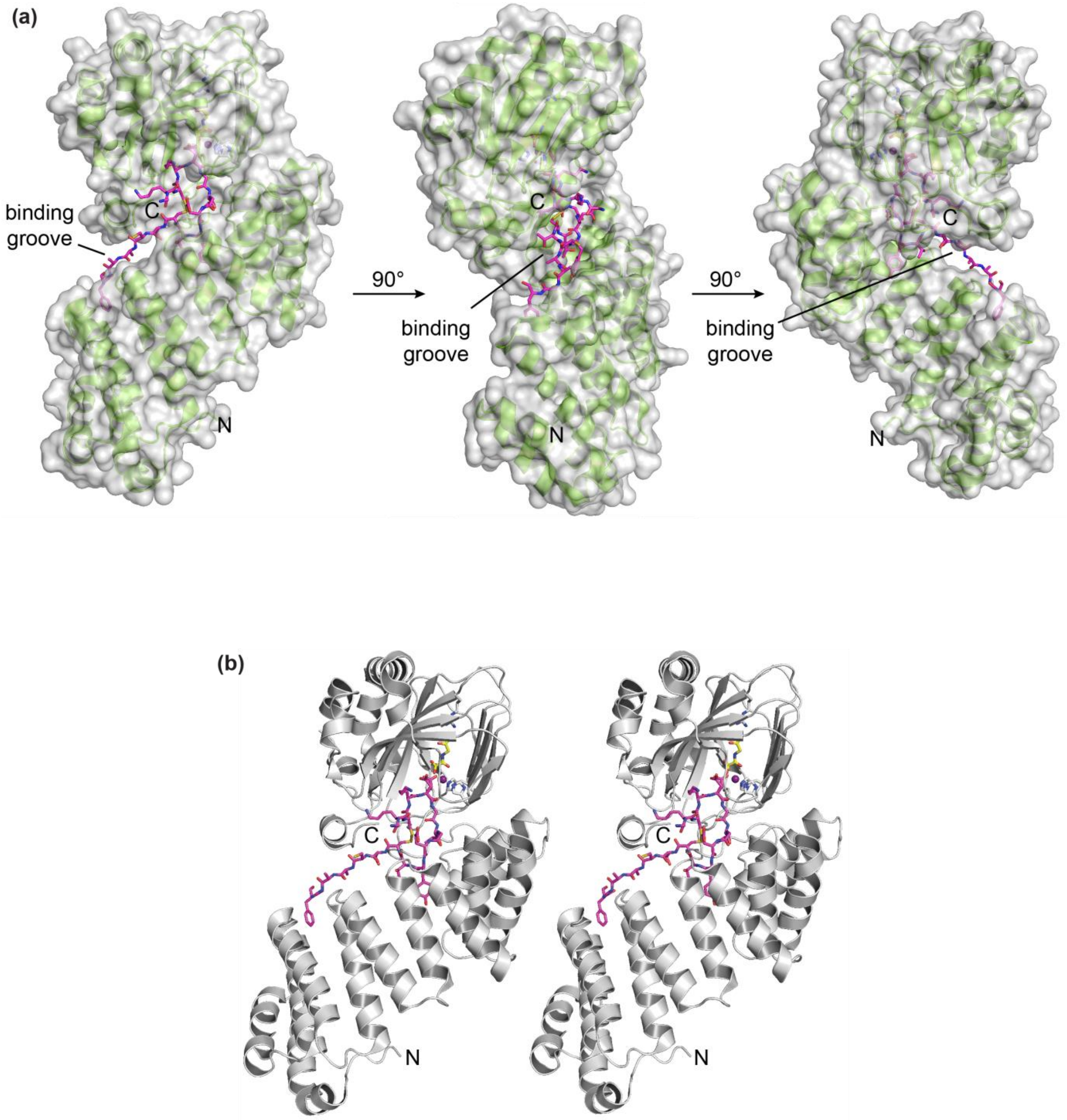

Supplementary Figure 10. Analysis of electron density OMIT maps (mFo−DFc) of the hFX derived NC39mer peptide supports the presence of two side chain conformers for the substrate hydroxylation target residue Asp103$_{hFX}$ in the AspH-TPR-Ox:hFX structure. (a) The blue density map (contoured to 3.0 σ) was calculated by omitting the entire NC39mer peptide from the map calculation. The green (contoured to 3.0 σ) and red (contoured to -3.0 σ) density maps were calculated by omitting Asp103$_{hFX}$ conformer B and setting occupancy of conformer A to 1.0. **(b)** The blue density map (contoured to 3.0 σ) was calculated by omitting the entire NC39mer peptide from the map calculation. Green (contoured to 3.0 σ) and red (contoured to -3.0 σ) density maps were calculated by omitting Asp103$_{hFX}$ conformer A and setting occupancy of conformer B to 1.0. Similar analysis of electron density maps of other AspH-bound substrates confirmed the presence of two side chain conformers in all other AspH-substrate crystal structures (see Supplementary Figures 16c, 17d, and 18d).

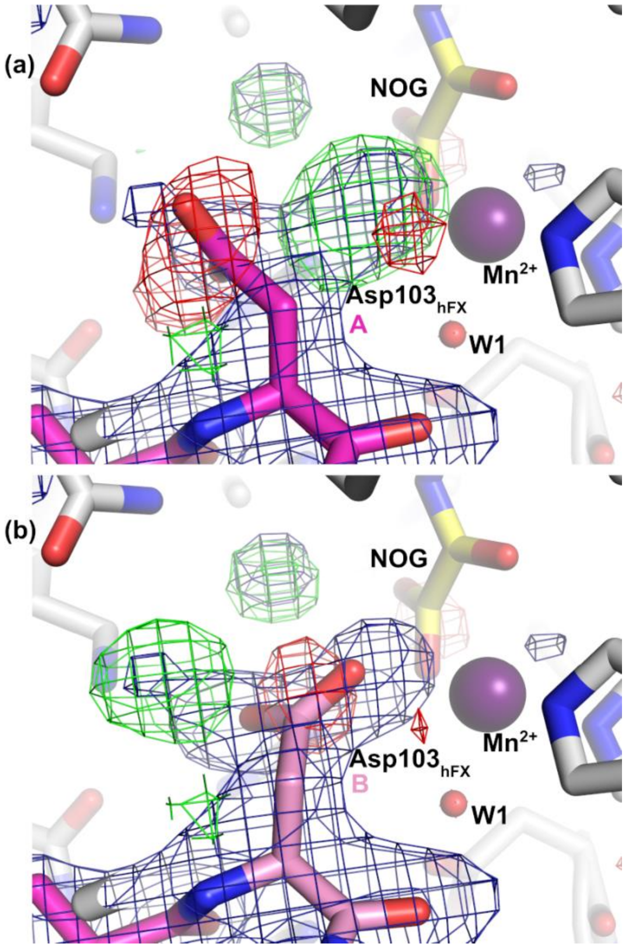

Supplementary Figure 11. Sequence alignment of five human EGFDs bearing the AspH-consensus sequence (human clotting factors X, IX, VII; human protein C and Z). The following residues are highlighted: Six cysteine residues of EGFDs bearing the AspH-consensus sequence (blue, columns 111-150); site of β-hydroxylation (red); conserved Phe- and Tyr-residues (green); conserved aromatic residues (Phe, Tyr, Try) interacting with the TPR-domain of AspH (yellow). Full-length sequences were obtained from the National Center for Biotechnology Information database[1] and processed using GeneDoc 2.7[2]; black shading indicates high sequence conservation.

```
             .
                      *        20         *        40         *        60         *        80
FA10_HUMAN : -MGRPLHLVLLSASLAGLLLLG-------------------ESLFIRREQANNILARVTRANS-FLEEMKKGHLERECME :  59
FA9_HUMAN  : -MQRVNMIMAESPGLITICLLGYLLSAE-------------CTVFLDHENANKILNRPKRYNSGKLEEFVQGNLERECME :  66
FA7_HUMAN  : MVSQALRLLCLLLGLQGCLAAGGVAKASGGETRDMPWKPGPHRVFVTQEEAHGVLHRRRRANA-FLEELRPGSLERECKE :  79
PROZ_HUMAN : ----MAGCVPLLQGLVLVLALHRVEP-----------------SVFLPASKANDVLVRWKRAGSYLLEELFEGNLEKECYE :  60
PROC_HUMAN : -MWQLTSLLLFVATWGISGTPAPLD-----------------SVFSSSERAHQVLRIRKRANS-FLEELRHSSLERECIE :  61

                      *       100         *       120         *       140         *       160
FA10_HUMAN : ETCSYEEAREVFEDSDKTNEFWNKYKDGDQCETSPCQN--------QGKCKDGLGEYTCTCLEGFEGKNCELFTRK--LC : 129
FA9_HUMAN  : EKCSFEEAREVFENTERTTEFWKQYVDGDQCESNPCLN--------GGSCKDDINSYECWCPFGFEGKNCELDVT----C : 134
FA7_HUMAN  : EQCSFEEAREIFKDAERTKLFWISYSDGDQCASSPCQN--------GGSCKDQLQSYICFCLPAFEGRNCETHKDDQLIC : 151
PROZ_HUMAN : EICVYEEAREVFENEVVTDEFWRRYKGGSPCISQPCLH--------NGSCQDSIWGYTCTCSPGYEGSNCELAKN--ECH : 130
PROC_HUMAN : EICDFEEAKEIFQNVDDTLAFWSKHVDGDQCLVLPLEHPCASLCCGHGTCIDGIGSFSCDCRSGWEGRFCQREVS-FLNC : 140

                      *       180         *       200         *       220         *       240
FA10_HUMAN : SLDNGDCDQFCHE-EQNSVVCSCARGYTLADNGKACIPTGPYPCGKQTLERRKRSVAQATSSSGEAPDSITWKPYDAADL : 208
FA9_HUMAN  : NIKNGRCEQFCKNSADNKVVCSCTEGYRLAENQKSCEPAVPFPCGRVSVSQTSKLTRAET----------VFPDVDYVNS : 204
FA7_HUMAN  : VNENGGCEQYCSDHTGTKRSCRCHEGYSLLADGVSCTPTVEYPCGKIPILEKR--------------------------- : 204
PROZ_HUMAN : PERTDGCQHFCLP-GQESYTCSCAQGYRLGEDHKQCVPHDQCACGVLTSEKRAP-------------------------- : 183
PROC_HUMAN : SLDNGGCTHYCLEEVGWRR-CSCAPGYKLGDDLLQCHPAVKFPCGRPWKRMEKKRSHLKR-------------------- : 199

                      *       260         *       280         *       300         *       320
FA10_HUMAN : DPTENPFDLLDFNQTQPERGDNNLTRIVGGQECKDGECPWQALLINEENEGFCGGTILSEFYILTAAHCLYQAKRFK--- : 285
FA9_HUMAN  : TEAETILD----NITQSTQSFNDFTRVVGGEDAKPGQFPWQVVLNG-KVDAFCGGSIVNEKWIVTAAHCVETGVKIT--- : 276
FA7_HUMAN  : ------------NASKPQG------RIVGGKVCPKGECPWQVLLLV-NGAQLCGGTLINTIWVVSAAHCFDKIKNWRNLI : 265
PROZ_HUMAN : ---------------------------------DLQDLPWQVKLTNSEGKDFCGGVIIRENFVLTTAKCSLLHRNIT--- : 227
PROC_HUMAN : --------------DTEDQEDQVDPRLIDGKMTRRGDSPWQVVLLDSKKKLACGAVLIHPSWVLTAAHCMDESKKLL--- : 262

                      *       340         *       360         *       380         *       400
FA10_HUMAN : VRVGDRNTEQEEGGEAVHEVEVVIKHNRFTKET--YDFDIAVLRLKTPITFRMNVAPACLPERDWAESTLMTQKTG-IVS : 362
FA9_HUMAN  : VVAGEHNIEETEHTEQKRNVIRIIPHHNYNAAINKYNHDIALLELDEPLVLNSYVTPICIADKEYTN-IFLKFGSG-YVS : 354
FA7_HUMAN  : AVLGEHDLSEHDGDEQSRRVAQVIIPSTYVPGT--TNHDIALLRLHQPVVLTDHVVPLCLPERTFSERTLAFVRFS-LVS : 342
PROZ_HUMAN : ---VKTYFNRTSQDPLMIKITHVHVHMRYDADAG--ENDLSLLELEWPIQCPGAGLPVCTPEKDFAEHLLIPRTRG-LLS : 301
PROC_HUMAN : VRLGEYDLRRWEKWELDLDIKEVFVHPNYSKSTT--DNDIALLHLAQPATLSQTIVPICLPDSGLAERELNQAGQETLVT : 340

                      *       420         *       440         *       460         *       480
FA10_HUMAN : GFGRTHE-----KGRQSTRLKMLEVPYVDRNSCKLSS-----SFIITQNMFCAGYDTKQEDACQGDSGGPHVTRFKDTYF : 432
FA9_HUMAN  : GWGRVFH-----KGRSALVLQYLRVPLVDRATCLRST-----KFTIYNNMFCAGFHEGGRDSCQGDSGGPHVTEVEGTSF : 424
FA7_HUMAN  : GWGQLLD-----RGATALELMVLNVPRLMTQDCLQQSRKVGDSPNITEYMFCAGYSDGSKDSCKGDSGGPHATHYRGTWY : 417
PROZ_HUMAN : GWARNGT-----DLGN--SLTTRPVTLVEGEECGQVLN-----VTVTTRTYCE---RSSVAAMHWMDGSVVTREHRGSWF : 366
PROC_HUMAN : GWGYHSSREKEAKRNRTFVLNFIKIPVVPHNECSEVMS-----NMVSENMLCAGILGDRQDACEGDSGGPMVASFHGTWF : 415

                      *       500         *       520         *
FA10_HUMAN : VTGIVSWGEGCARKGKYGIYTKVTAFLKWIDRSMKTRGLPKAKSHAPEVITSSPLK : 488
FA9_HUMAN  : LTGIISWGEECAMKGKYGIYTKVSRYVNWIKEKTKLT------------------- : 461
FA7_HUMAN  : LTGIVSWGQGCATVGHFGVYTRVSQYIEWLQKLMRSE-------PRPGVLLRAPFP : 466
PROZ_HUMAN : LTGVLGS-QPVGGQAHMVLVTKVSRYSLWFKQIMN--------------------- : 400
PROC_HUMAN : LVGLVSWGEGCGLLHNYGVYTKVSRYLDWIHGHIRDKEAPQKSWAP---------- : 461
```

Supplementary Figure 12 (continues on the next page). MS-Analysis of EGF1 of human Coagulation Factor X (hFX EGF1$_{39mer}$) synthesized by thiol oxidation from the linear peptide in air-saturated buffer to form disulfides suggests the presence of several disulfide isomers. MS-Analysis was performed using a Q Exactive^{TM} Hybrid Quadrupole-Orbitrap^{TM} mass spectrometer coupled to a Dionex^{TM} UltiMate$^{®}$ 3000 HPLC (Phenomenix Aeris^{TM} WIDEPORE 3.6 µm XB-C8 LC-column, 50x2.1 mm, pore size of 200 Å) and equipped with a heated ESI ionization source.

(a) MS-Analysis of hFX EGF1$_{39mer}$ (aa 86-124) synthesized by thiol oxidation of the reduced peptide in air-saturated buffer: **(1)** Schematic EGF domain structure and calculated mass of the expected disulfide isomer of hFX EGF1$_{39mer}$ featuring the 'canonical' disulfide connectivity pattern (Cys 1-3, 2-4, 5-6; green); **(2)** LC-MS analysis (UV absorbance and MS traces) of hFX EGF1$_{39mer}$ manifests a broad peak at 4.4 min consistent with co-elution of multiple isomers (red box); **(3)** Blank sample (buffer without hFX EGF1$_{39mer}$) demonstrating that the remaining peaks in (2) correspond to buffer (0.5 min) and plasticizers (5.0 to 9.0 min; light blue box); **(4)** Close-up of the peak of interest from spectrum **2** (4.0 to 5.0 min) showing the peak tailing in detail; **(5)** Only the most abundant +3, +4 (1062.2, 100% relative abundance), and +5 charge states corresponding to the mass of hFX EGF1$_{39mer}$ are visible excluding the possibility of the presence of a species with a different mass from hFX EGF1$_{39mer}$; **(6)** The deconvoluted mass spectrum showing only the expected mass (4242.7 Da) of hFX EGF1$_{39mer}$ supporting the presence of a mixture of 'canonical' and 'non-canonical' disulfide isomers.

(1)

hFX EGF1$_{39mer}$ (aa 86-124, Cys 1-3, 2-4, 5-6):

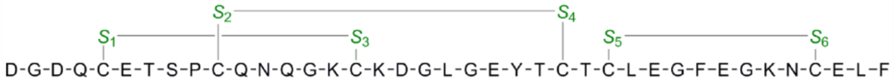

MS (ESI): m/z calculated for $C_{173}H_{259}O_{66}N_{47}S_6$ $[M+H]^+$: 4242.71, found: 4242.69

Purity (HPLC): >95%

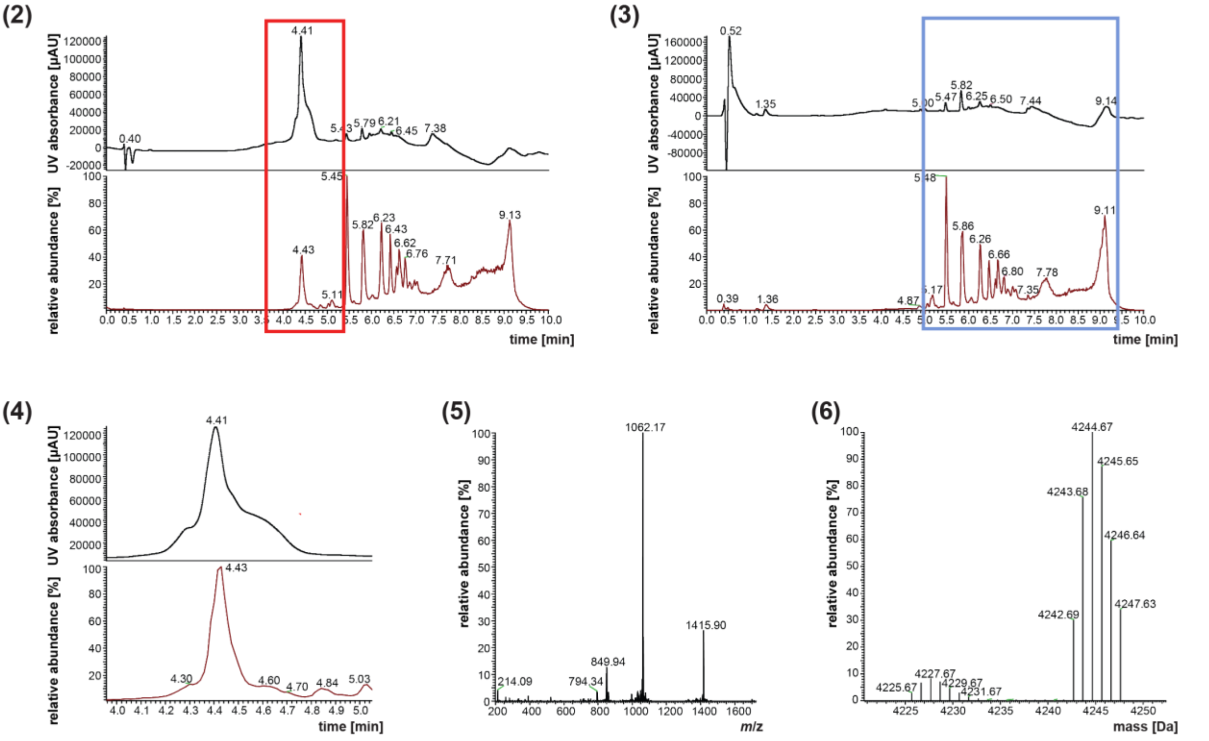

(b) MS-Analysis of hFX $EGF1_{39mer}$ (aa 86-124); the disulfide bonds were reduced using tris(2-carboxyethyl)phosphine (TCEP) in 50 mM aqueous citrate buffer (pH = 3.0): **(1)** Schematic structure and calculated mass of the reduced linear hFX $EGF1_{39mer}$; **(2)** LC-MS analysis (UV absorbance and MS traces) of the reduced linear hFX $EGF1_{39mer}$ shows a sharper peak at 4.6 min (red box) when compared to the broad peak of the oxidized form; **(3)** Blank sample (buffer without reduced linear hFX $EGF1_{39mer}$) demonstrating that the remaining peaks in (2) correspond to buffer (0.5 min) and plasticizers (5.0 to 9.0 min; light blue box); **(4)** Close-up view of the peak of interest from spectrum **2** (4.0 to 5.0 min); **(5)** Only the most abundant +3, +4 (1063.7, 100% relative abundance), and +5 charge states, corresponding to the mass of the reduced linear hFX $EGF1_{39mer}$, are visible; **(6)** Deconvoluted mass spectrum showing only the expected mass (4248.7 Da) of hFX $EGF1_{39mer}$ which supports the presence of a mixture of ‘canonical’ and ‘non-canonical’ disulfide isomers in the sample obtained by thiol oxidation in air-saturated buffer.

(1)

hFX $EGF1_{39mer}$ (reduced):

D-G-D-Q-C-E-T-S-P-C-Q-N-Q-G-K-C-K-D-G-L-G-E-Y-T-C-T-C-L-E-G-F-E-G-K-N-C-E-L-F

MS (ESI): m/z calculated for $C_{173}H_{265}O_{66}N_{47}S_6$ $[M+H]^+$: 4248.71, found: 4248.72
Purity (HPLC): >95%

(2)

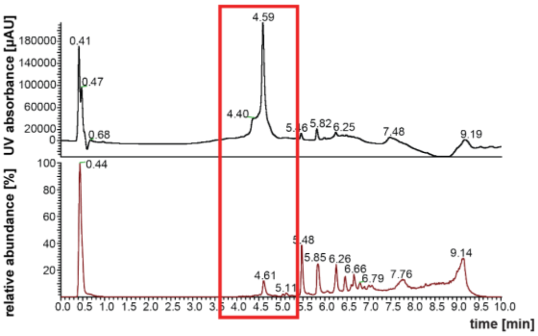

(3)

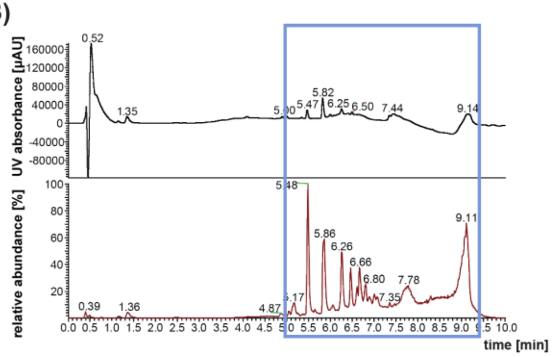

(4)

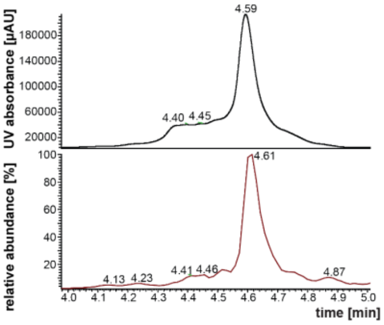

(5)

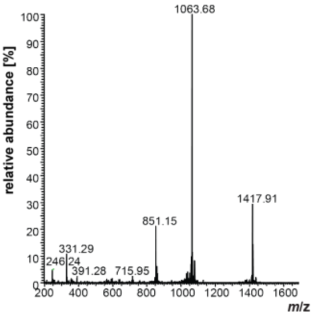

(6)

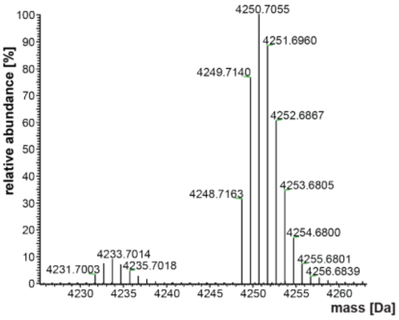

Supplementary Figure 13 (continues on the next page). MS-Studies of EGF1 of human Coagulation Factor X (hFX EGF1$_{39mer}$) synthesized by thiol oxidation from the linear peptide in air-saturated buffer to form disulfides indicates the presence of at least two major disulfide bond isomers, one featuring a 'non-canonical' disulfide connectivity pattern (Cys 1-2, 3-4, 5-6).

(a) MS/MS-Analysis using a Bruker Daltonics Ultraflex I^{TM} MALDI-ToF/ToF mass spectrometer of an aqueous solution of the commercial hFX EGF1$_{39mer}$ (aa 86-124). The peptide was neither exposed to His$_6$-tagged AspH$_{315-758}$, trypsin, nor to reducing agents. In general, cyclic peptides formed by disulfides bridges are resistant to fragmentation in the gas phase[7,8], giving rise to distinct MS-fragmentation patterns. The obtained MS/MS fragmentation data **(1)** indicate the presence of a characteristic disulfide isomer featuring a non-canonical disulfide connectivity pattern (Cys 1-2, 3-4, 5-6) as evidenced by the presence of the y_{24}–y_{29} fragment ions **(2)**, which cannot be explained considering the canonical (Cys 1-3, 2-4, 5-6) nor other disulfide isomers **(3)**.

(1)

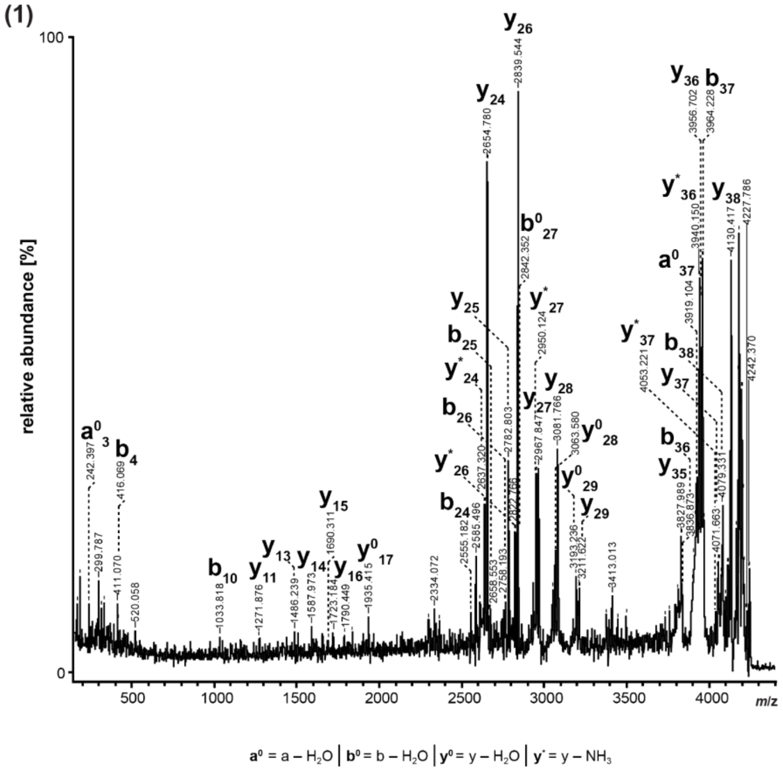

(2)

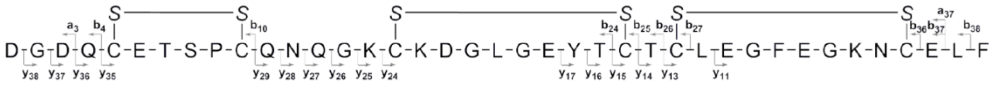

Purity (HPLC): >95%

MS (MALDI): m/z calculated for $C_{173}H_{259}O_{65}N_{47}S_6$ $[M+H]^+$: 4242.71, found: 4243.2

(3)

'non-canonical' hFX EGF1$_{39mer}$ isomer (aa 86-124, Cys 1-2, 3-4, 5-6):

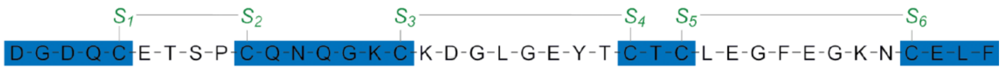

'canonical' hFX EGF1$_{39mer}$ isomer (aa 86-124, Cys 1-3, 2-4, 5-6):

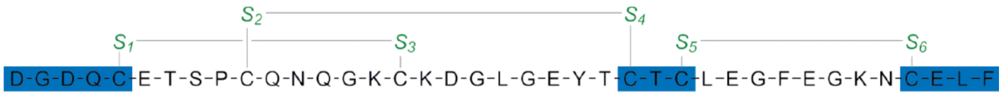

: expected fragment ions

(b) MALDI-ToF MS Analysis of hFX EGF1$_{39mer}$ (aa 86-124, synthesized by thiol oxidation in air-saturated buffer) after denaturation (6 M urea) and digestion with trypsin (16 h at 37 °C in 100 mM phosphate buffer, pH = 6.6) under non-reducing conditions. The peptide was not exposed to His$_6$-tagged AspH$_{315-758}$. Trypsin cleavage sites[9] of the non-canonical (Cys 1-2, 3-4, 5-6; top, green) and canonical (Cys 1-3, 2-4, 5-6; bottom, green) disulfide isomers of hFX EGF1$_{39mer}$ are in purple **(1)**. The MS data indicate the presence of a characteristic disulfide isomer featuring a non-canonical disulfide connectivity pattern (Cys 1-2, 3-4, 5-6) and presumably the canonical isomer **(2)**: Trypsin cleavage fragments A-C originating from the non-canonical disulfide connectivity pattern and fragments D and E from the expected canonical disulfide pattern. Close-ups of the relevant peaks and assignment to the corresponding ions are shown in **(3)** to **(5)**. In some experiments, -18 Da peaks were observed which are MALDI artefacts likely resulting from loss of H_2O. Guanidination of Lys-residues with *O*-methylisourea improves the ionization properties of the fragments significantly; the relative abundance of fragment A is similar to that of fragments B/C.

(1)

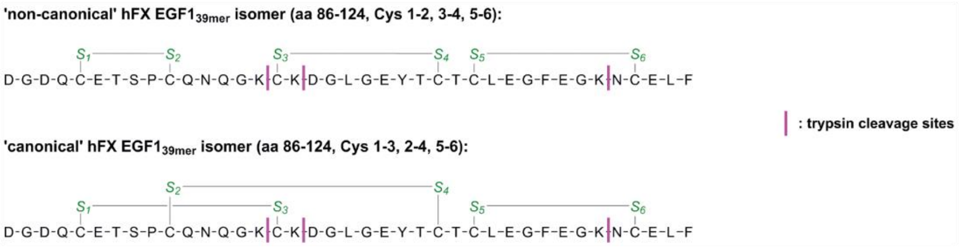

(2)

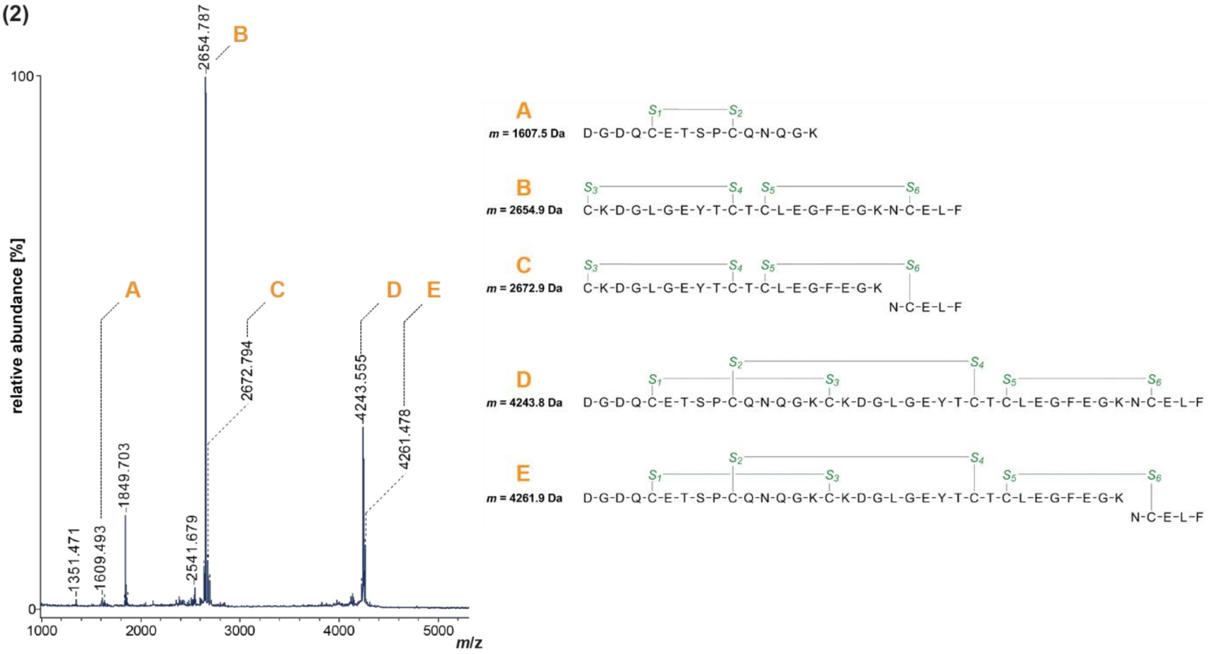

(3) **(4)** **(5)**

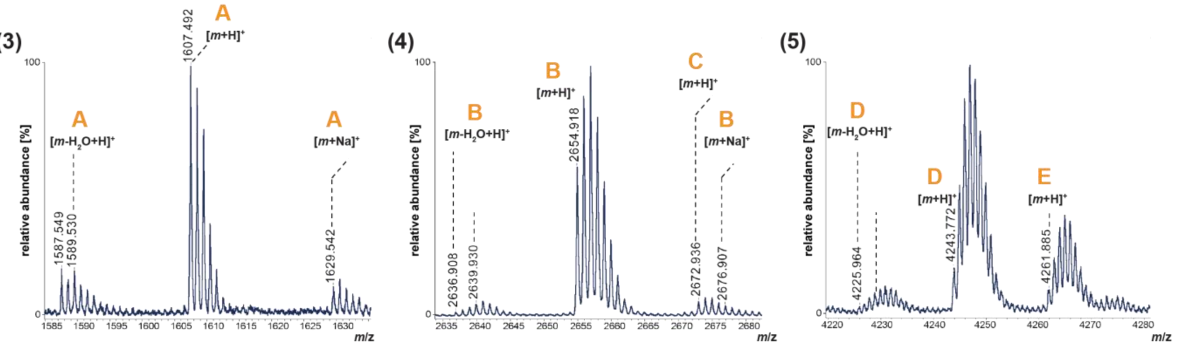

Supplementary Figure 14 (continues on the 3 following pages). Cyclic peptides (CP), mimicking the 10-membered Cys3−Cys4 ring formed by a ‘non-canonical’ EGFD-disulfide pattern (Cys 1−2, 3−4, 5−6), are substrates for His$_6$-AspH$_{315\text{-}758}$. Schematic structures, HPLC purification gradient, purity, and high resolution mass spectrometric data (HRMS) of the synthesized cyclic peptides derived from hFX EGF1$_{39mer}$ (aa 101-110 core ring residues) are shown for the 9 different potential AspH-substrates. The synthesis of the cyclic peptides derived from hFX EGF1$_{39mer}$ (aa 101-110 core ring residues) was performed as in the Methods Section; ‘^{D}‘ indicates D-stereochemistry of the amino acid. All end-point turnover assays were performed as outlined in the Methods Section. Light orange graphs represent parallel controls in which AspH was replaced by buffer (no-enzyme control). Only the mass difference corresponding to a single oxidation event (+16 Da) was detected, i.e. higher oxidized species (e.g. +32 Da) were not observed.
Standard non redox buffer: 50 mM HEPES, pH = 7.5, 150 mM NaCl; Redox buffer: 50 mM Tris, pH = 8.5, 3.0 mM L-glutathione thiol, 0.3 mM L-glutathione disulphide, 150 mM NaCl.

(a) Asp103$_{hFX}$-β-hydroxylation of CP$_{101\text{-}110}$: **(1)** Schematic structure, HPLC purification gradient, purity, and HRMS data of CP$_{101\text{-}110}$; the hydroxylation site (Asp103$_{hFX}$) is in red; **(2)** ~88% conversion was observed under standard (non-redox) buffer conditions.

(1)

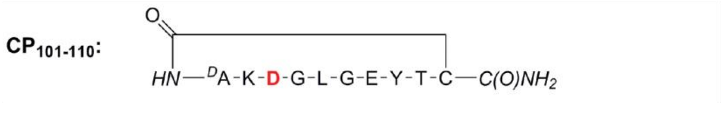

HPLC-gradient: 0-30% CH_3CN in H_2O containing 0.1% TFA (30 min).
Purity (HPLC): >95%
HRMS (ESI): m/z calculated for $C_{46}H_{71}O_{17}N_{12}S$ $[M+H]^+$: 1095.4775, found: 1095.4740.

(2)

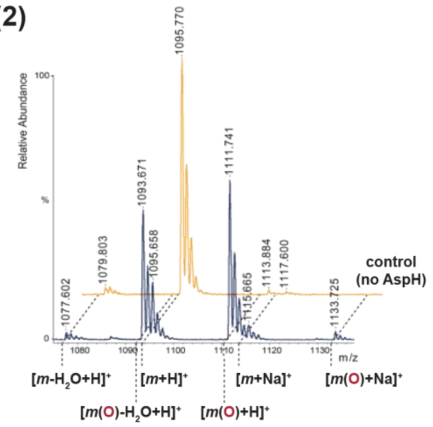

(b) Asp103$_{hFX}$-β-hydroxylation of CP$_{101\text{-}115}$ indicating that an extended substrate C-terminus does not substantially promote hydroxylation: **(1)** Schematic structure, HPLC purification gradient, purity, and HRMS data of CP$_{101\text{-}115}$; the hydroxylation site (Asp103$_{hFX}$) is in red; **(2)** ~85% conversion was observed under standard (non-redox) buffer conditions.

(1)

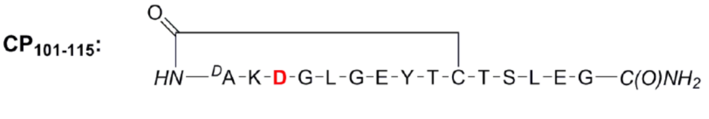

HPLC-gradient: 0-35% CH_3CN in H_2O containing 0.1% TFA (35 min).
Purity (HPLC): >95%
HRMS (ESI): m/z calculated for $C_{66}H_{104}O_{26}N_{17}S$ $[M+H]^+$: 1582.7054, found: 1582.7013.

(2)

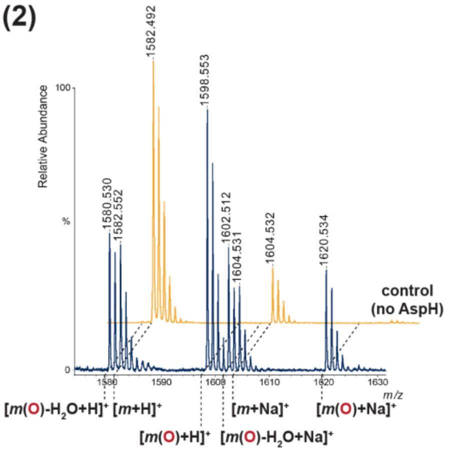

(c) Efficient Asp103$_{hFX}$-β-hydroxylation of CP$_{101-119}$ indicating that CP$_{101-119}$ is a more efficient AspH-substrate than CP$_{101-110}$ or CP$_{101-115}$. This may relate to the interaction of the side chain of the Phe-residue with the TPR-domain as evidenced by X-ray analysis (see Main Text): **(1)** Schematic structure, HPLC purification gradient, purity, and HRMS data of CP$_{101-119}$; the hydroxylation site (Asp103$_{hFX}$) is in red; **(2)** >95% conversion was observed under standard (non-redox) buffer conditions.

(1)

CP$_{101-119}$:

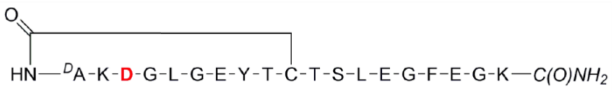

HPLC-gradient: 0-35% CH_3CN in H_2O containing 0.1% TFA (35 min).
Purity (HPLC): >95%
HRMS (ESI): m/z calculated for $C_{88}H_{135}O_{32}N_{22}S$ $[M+H]^+$: 2043.9328, found: 2043.9347

(2)

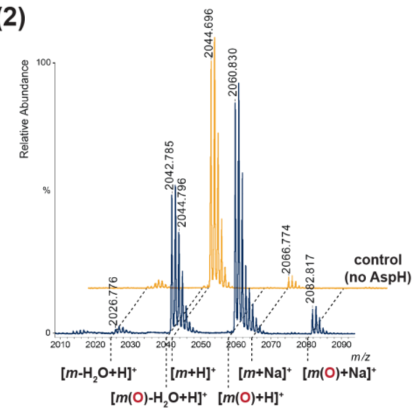

(d) Efficient Asn103$_{hFX}$-β-hydroxylation of CP$_{101-110}$(Asn103) indicates a potential slight preference of His$_6$-AspH$_{315-758}$ for the Asn- over the Asp-containing CP$_{101-110}$-substrate: **(1)** Schematic structure, HPLC purification gradient, purity, and HRMS data of CP$_{101-110}$(Asn103); the hydroxylation site (Asn103$_{hFX}$) is in red; **(2)** >95% conversion was observed under standard (non-redox) buffer conditions.

(1)

CP$_{101-110}$(Asn103):

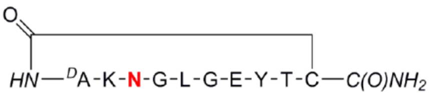

HPLC-gradient: 0-30% CH_3CN in H_2O containing 0.1% TFA (30 min).
Purity (HPLC): >95%
HRMS (ESI): m/z calculated for $C_{46}H_{72}O_{16}N_{13}S$ $[M+H]^+$: 1094.4935, found: 1094.4906.

(2)

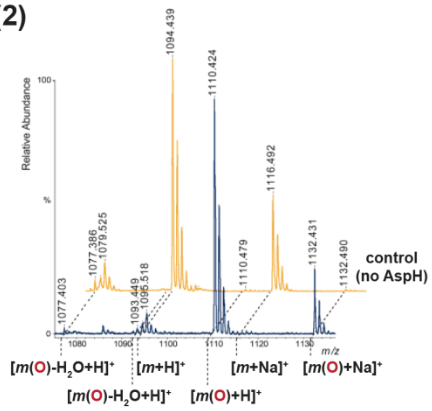

(e) Asp103$_{hFX}$-β-hydroxylation of CP$_{101-110}$(Ala108) does not proceed supporting the proposed consensus sequence of AspH-substrates: **(1)** Schematic structure, HPLC purification gradient, purity, and HRMS data of CP$_{101-110}$(Ala108); the hydroxylation site (Asp103$_{hFX}$) is in red; **(2)** no conversion was observed under standard (non-redox) buffer conditions.

(1)

CP$_{101-110}$(Ala108):

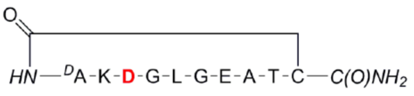

HPLC-gradient: 0-50% CH_3CN in H_2O containing 0.1% TFA (50 min).
Purity (HPLC): >95%
HRMS (ESI): m/z calculated for $C_{40}H_{67}O_{16}N_{12}S$ $[M+H]^+$: 1003.4513, found: 1003.4522.

(2)

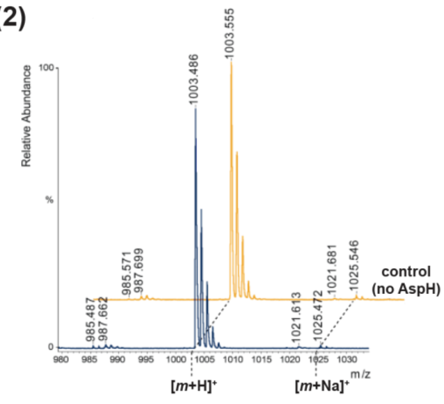

(f) Asp103$_{hFX}$-β-hydroxylation of CP$_{101\text{-}110}$(Val108) proceeds suggesting a positive impact on AspH-catalysis when a more lipophilic valine residue is incorporated at residue 108 compared to alanine as in **(e)**: **(1)** Schematic structure, HPLC purification gradient, purity, and HRMS data of CP$_{101\text{-}110}$(Val108); the hydroxylation site (Asp103$_{hFX}$) is in red; **(2)** ~5% conversion was observed under standard (non-redox) buffer conditions. Note, that peptide hydroxylation is significantly less efficient than for CP$_{101\text{-}110}$ bearing Tyr108 (see **a**).

(1)

CP$_{101\text{-}110}$(Val108):

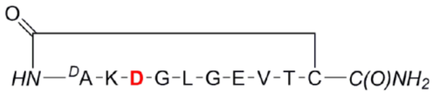

HPLC-gradient: 0-35% CH_3CN in H_2O containing 0.1% TFA (35 min).
Purity (HPLC): >95%
HRMS (ESI): m/z calculated for $C_{42}H_{71}O_{16}N_{12}S$ $[M+H]^+$: 1031.4826, found: 1031.4794.

(2)

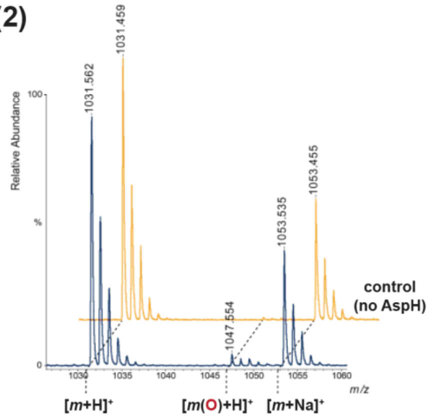

(g) Asp103$_{hFX}$-β-hydroxylation of CP2$_{101\text{-}110}$ **(a)**: **(1)** Schematic structure, HPLC purification gradient, purity, and HRMS data of CP2$_{101\text{-}110}$; the hydroxylation site (Asp103$_{hFX}$) is in red; **(2)** >95% conversion was observed under standard (non-redox) buffer conditions; **(3)** time-dependent hydroxylation of CP2$_{101\text{-}110}$ (% conversion in diamonds) was determined by MS at ambient temperature using a RapidFire RF360 high-throughput sampling robot attached to an Agilent 6530 Accurate-Mass Quadrupole time-of-flight (Q-TOF) mass spectrometer operating in the positive ionization mode (Agilent)[10]. Conditions: Fragment voltage: 150 V; solvents: acetonitrile/water (85/15) and water containing 0.1% aqueous formic acid; sampling interval: 43 seconds; **(4)** ion counts of non-hydroxylated (circles) and hydroxylated (squares) CP2$_{101\text{-}110}$ decrease/increase are shown in a time-dependent manner. The total ion count (triangles, sum of hydroxylated and non-hydroxylated CP2$_{101\text{-}110}$ ions) is constant within experimental error throughout the experiment; the extent of hydroxylation of AspH-substrates can be quantified based on the ion counts observed in MS.

(1)

CP2$_{101\text{-}110}$:

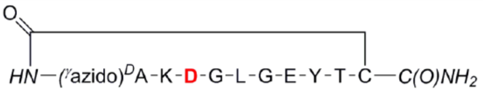

HPLC-gradient: 0-30% (linear) CH_3CN in H_2O containing 0.1% TFA (30 min).
Purity (HPLC): >95%
HRMS (ESI): m/z calculated for $C_{46}H_{70}O_{17}N_{15}S$ $[M+H]^+$: 1136.4789, found: 1136.4756.

(2)

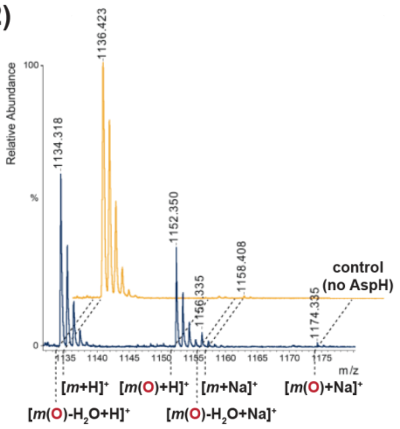

(3)

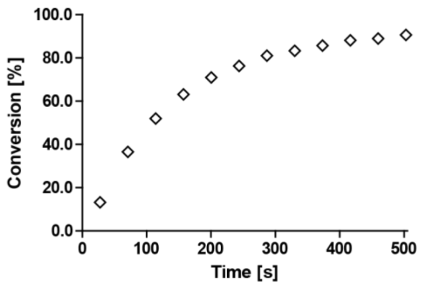

(4)

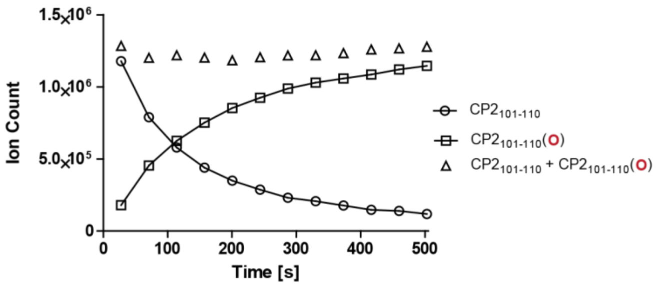

(h) Asn103$_{hFX}$-β-hydroxylation of CP2$_{101\text{-}110}$(Asn) has no detrimental influence on hydroxylation efficiency upon Asp/Asn-exchange when compared to **(g)**: **(1)** Schematic structure, HPLC purification gradient, purity, and HRMS data of CP2$_{101\text{-}110}$(Asn); the hydroxylation site (Asn103$_{hFX}$) is in red; **(2)** >95% conversion was observed under standard (non-redox) buffer conditions.

(1)

CP2$_{101\text{-}110}$(Asn103):

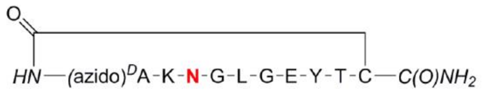

HPLC-gradient: 0-38% CH_3CN in H_2O containing 0.1% TFA (38 min).
Purity (HPLC): >95%
HRMS (ESI): m/z calcd for $C_{46}H_{71}O_{16}N_{16}S$ $[M+H]^+$: 1135.4949, found: 1135.4963.

(2)

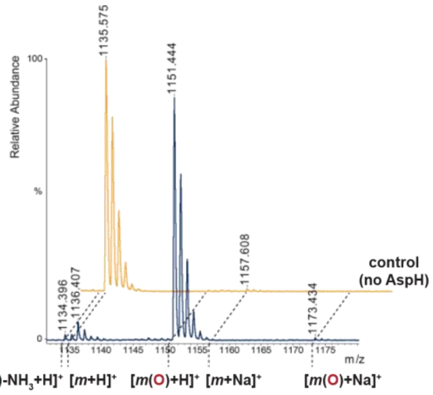

(i) Asn103$_{hFX}$-β-hydroxylation of LP2$_{101\text{-}110}$(Asn103) does not proceed consistent with the proposal that His$_6$-AspH$_{315\text{-}758}$ does not catalyze the hydroxylation of linear peptides: **(1)** Schematic structure, HPLC purification gradient, purity, and HRMS data of LP2$_{101\text{-}110}$(Asn103); the hydroxylation site (Asn103$_{hFX}$) is in red; **(2)** no conversion was observed under standard (non-redox) buffer conditions.

(1)

LP2$_{101\text{-}110}$(Asn103):

H$_2$N—(azido)^{D}A-K-**N**-G-L-G-E-Y-T-C—*C(O)NH$_2$*

HPLC-gradient: 0-30% CH_3CN in H_2O containing 0.1% TFA (30 min).
Purity (HPLC): >95%
HRMS (ESI): m/z calculated for $C_{44}H_{71}O_{15}N_{16}S$ $[M+H]^+$: 1095.5000, found: 1095.4753.

(2)

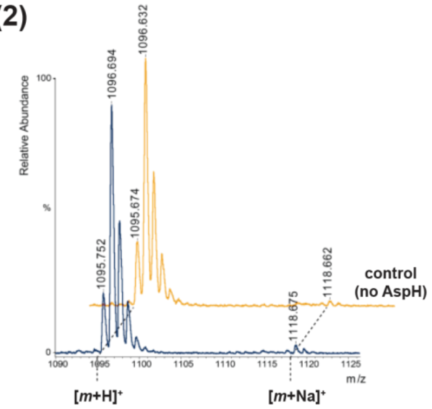

Supplementary Figure 15. NMR spectra of cyclic peptide $CP2_{101\text{-}110}$, before and after exposure to His_6-$AspH_{315\text{-}758}$ under standard assay conditions indicate that the AspH-catalyzed oxidation is regio- and stereo-selective. (a) Schematic structure of $CP2_{101\text{-}110}$ (The hydroxylation site, $Asp103_{hFX}$, is in red); **(b)** ^1H NMR (700 MHz, Bruker Avance III) of the non-hydroxylated $CP2_{101\text{-}110}$ (signals assigned based on 2D NMR spectra) in deuterated Tris-buffer (prepared in D_2O) in the presence of cofactors before addition of His_6-$AspH_{315\text{-}758}$; **(c)** 2-D ^1H,^{13}C-HSQC correlation spectrum (700 MHz, Bruker Avance III) of the lyophilized and resuspended hydroxylated $CP2_{101\text{-}110}$ (blue), obtained by exposure to His_6-$AspH_{315\text{-}758}$ under standard (non-reducing) conditions as given in the Methods Section, overlaid with the ^1H,^{13}C-HSQC correlation spectrum of the non-hydroxylated $CP2_{101\text{-}110}$ (green). Evidence for oxidation of the thioether sulfur atom was not accrued; the appearance of a single new signal (red circle; chemical shifts: 4.1 and 73.2 ppm) supports the expected stereoselective β-oxidation of Asp (aa 103). The degree of peptide hydroxylation was quantified in a separate experiment using MS which is shown in Supplementary Figures 14g(3) and 14g(4).
Standard non redox buffer: 50 mM HEPES, pH = 7.5, 150 mM NaCl; Redox buffer: 50 mM Tris, pH = 8.5, 3.0 mM L-glutathione thiol, 0.3 mM L-glutathione disulphide, 150 mM NaCl.

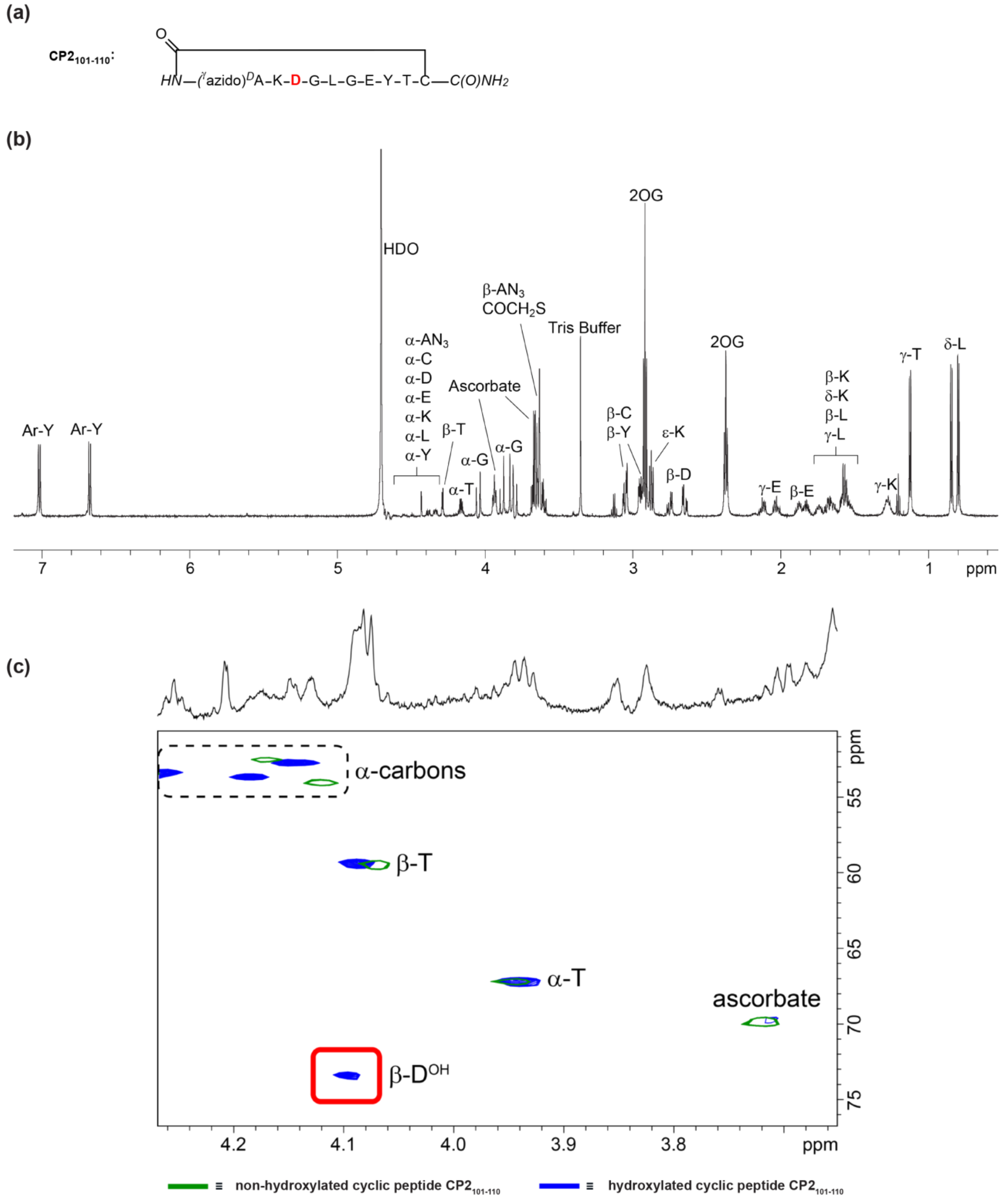

Supplementary Figure 16 (continues on the next page). Crystal structure of AspH-TPR-Ox:$CP_{101\text{-}119}$ (PDB entry: 6RK9). Color code: grey: His_6-$AspH_{315\text{-}758}$; yellow: carbon-backbone of *N*-oxalylglycine (NOG); green: carbon-backbone of the $CP_{101\text{-}119}$ peptide; violet: Mn(II); red: oxygen; blue: nitrogen; pale yellow: sulfur. w: water.

(a) Two His_6-$AspH_{315\text{-}758}$ molecules are present in the asymmetric unit of the AspH-TPR-Ox:$CP_{101\text{-}119}$ crystal structure, only one (chain A) contains bound $CP_{101\text{-}119}$ peptide (shown below): **(1)** Overview of His_6-$AspH_{315\text{-}758}$ bound to $CP_{101\text{-}119}$; **(2)** Interactions of $CP_{101\text{-}119}$ with His_6-$AspH_{315\text{-}758}$: His493 and Arg526 of the TPR domain interact with $Tyr108_{hFX}$ (3.0 respectively 2.8 Å) of the $CP_{101\text{-}119}$ peptide while Tyr565 interacts with $Cys110_{hFX}$ (3.0 Å); **(3)** Close-up of additional interactions of $CP_{101\text{-}119}$ with the AspH-TPR domain: Asn395 interacts with $Phe116_{hFX}$ of $CP_{101\text{-}119}$ (2.8 Å), while the main chain carbonyl of Phe432 interacts with $Gly115_{hFX}$ (2.9 Å); **(4)** Close-up of the key residues engaged in (co-)substrate binding in the AspH-active site: Two alternative conformations of $Asp103_{hFX}$ are present in the crystal structure (productive conformation shown in green, non-productive conformation shown in olive); **(5)** Close-up of the AspH-active site: The *pro-R* hydrogen at the $Asp103_{hFX}$ β-position of the productive $CP_{101\text{-}119}$ conformational isomer is positioned to interact with Mn(II) (distance Cβ-Mn: 4.3 Å).

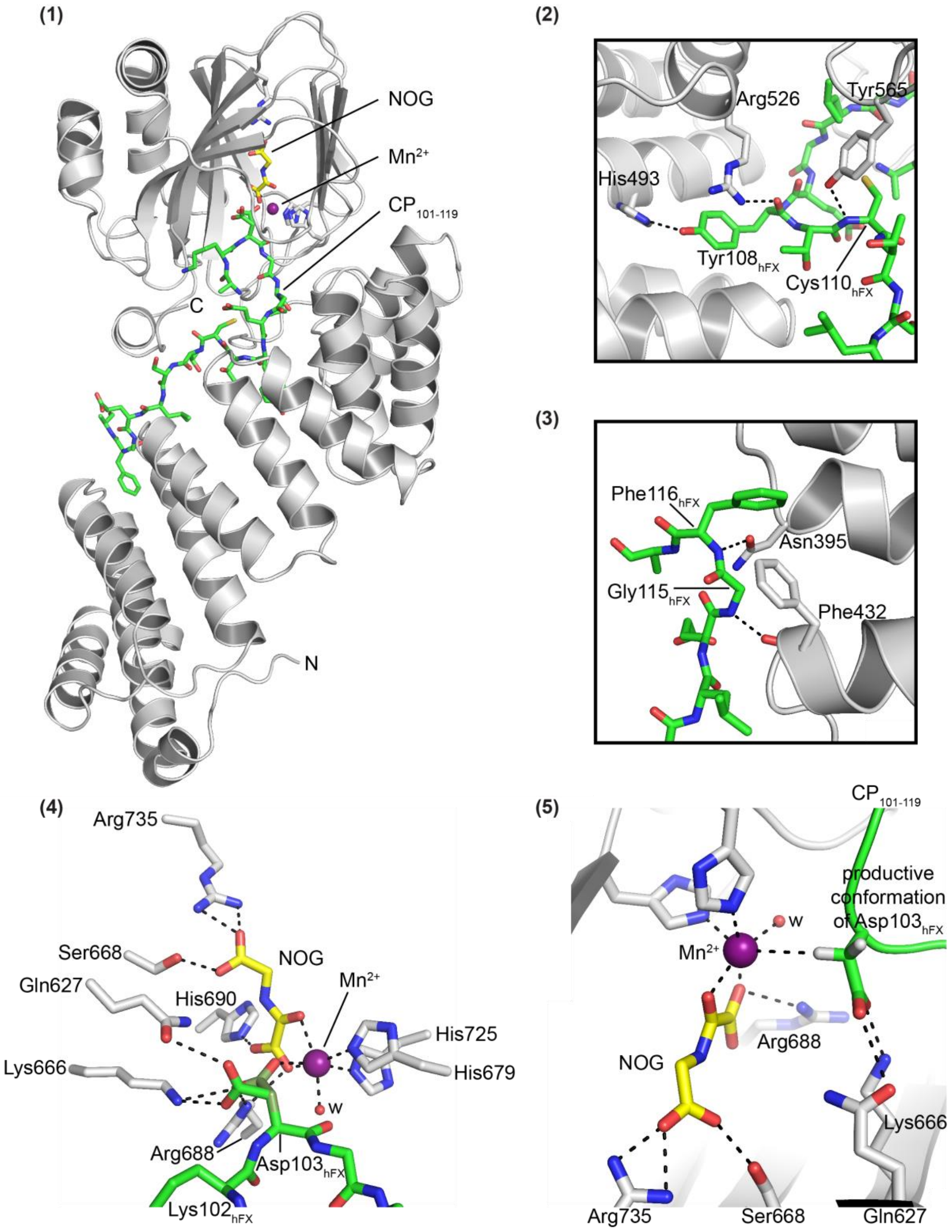

(b) Stereoview of the AspH-TPR-Ox:$CP_{101\text{-}119}$ crystal structure.

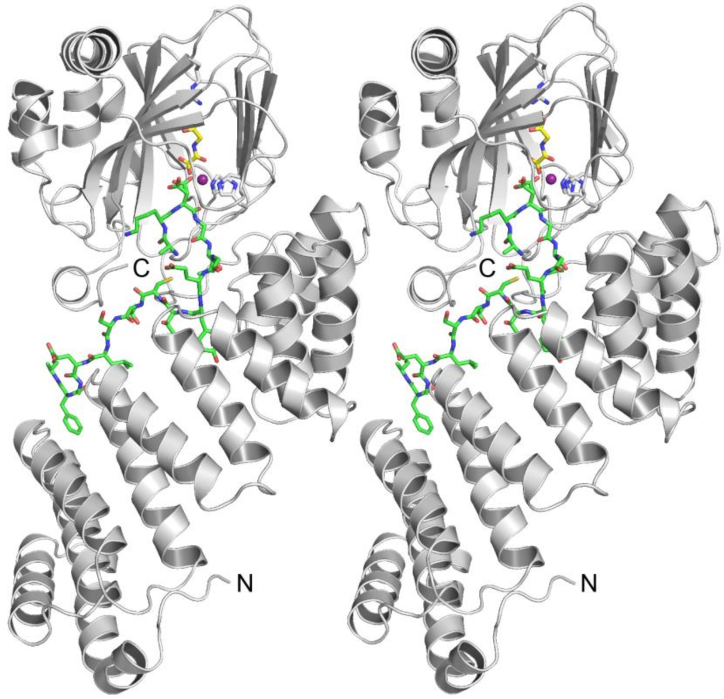

(c) OMIT electron density map ($mF_o{-}DF_c$) contoured to 3σ around the $CP_{101\text{-}119}$ peptide from the AspH-TPR-Ox:$CP_{101\text{-}119}$ crystal structure reveals electron density for two alternative conformations of $Asp103_{hFX}$. The thioether linking *N*-acetyl D-$Ala101_{hFX}$ and $Cys110_{hFX}$ could not be modelled accurately; however, the results of other analytical methods (MS, NMR) clearly indicate the presence of a macrocyclic thioether (see above).

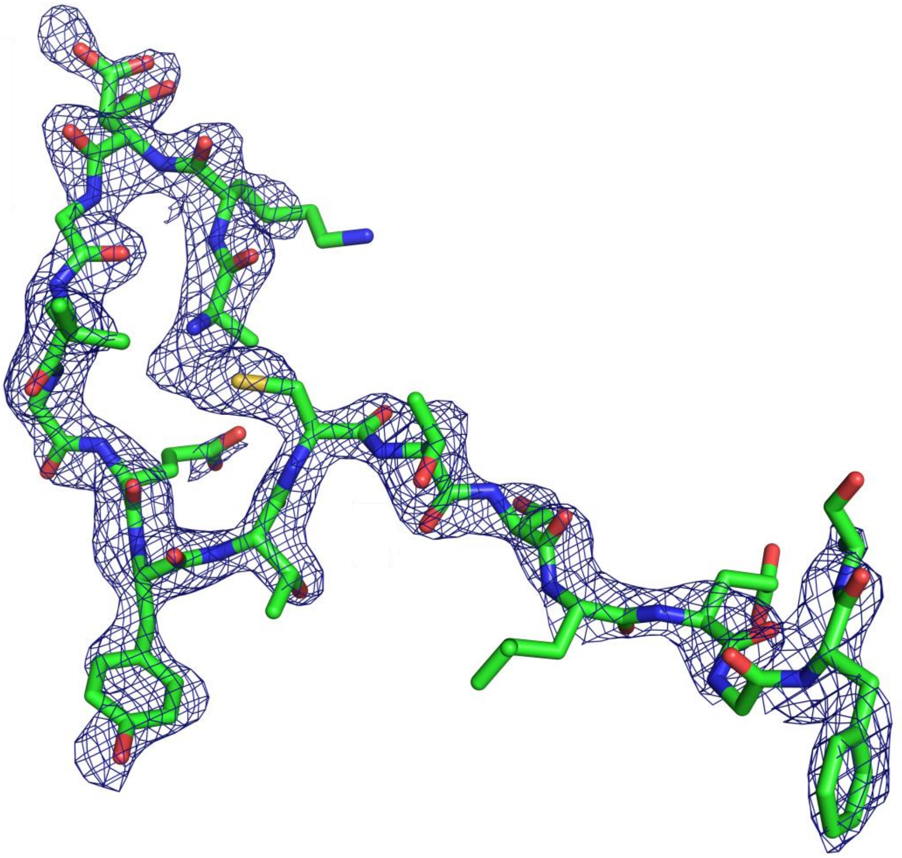

Supplementary Figure 17 (continues on the following 2 pages). Crystal structure of AspH-TPR-Ox:NC$_{26mer}$ (PDB entry: 5JZU). Color code: grey: His$_6$-AspH$_{315\text{-}758}$; yellow: carbon-backbone of *N*-oxalylglycine (NOG); salmon: carbon-backbone of the NC$_{26mer}$ peptide; violet: Mn(II); red: oxygen; blue: nitrogen; pale yellow: sulfur.

(a) His$_6$-AspH$_{315\text{-}758}$ binds the NC$_{26mer}$ peptide through interactions with active site residues, the hinge region, and the TPR domain: **(1)** Overview of the AspH-TPR-Ox:NC$_{26mer}$ crystal structure; **(2)** Close-up of the active site (different view with respect to **1**): Asp616 and Glu617 of the acidic loop interact with the NC$_{26mer}$ AspH-substrate. Furthermore, Gln627, Lys666, and Arg688 interact with Asp103$_{hFX}$ while Arg686 interacts with Lys102$_{hFX}$ (2.9 Å). Two alternative conformations of Asp103$_{hFX}$ are present in the crystal structure; **(3)** Interactions of NC$_{26mer}$ peptide with His$_6$-AspH$_{315\text{-}758}$: His493 and Arg526 of the TPR domain interact with Tyr108$_{hFX}$ of the NC$_{26mer}$ AspH-substrate (3.1 respectively 2.8 Å) while Tyr565 interacts with Cys110$_{hFX}$ (2.9 Å).

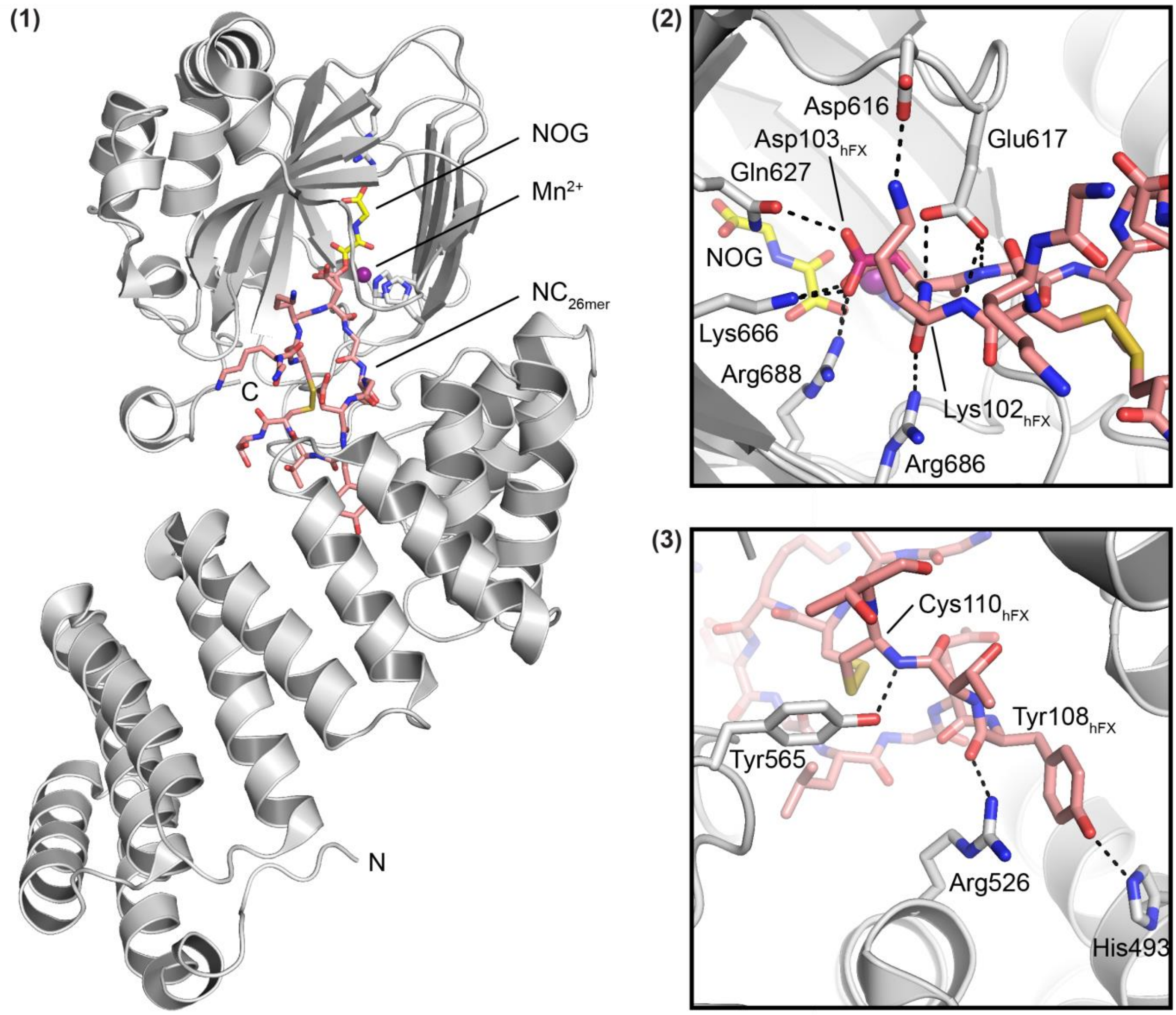

(b) Stereoview of the AspH-TPR-Ox:NC_{26mer} crystal structure.

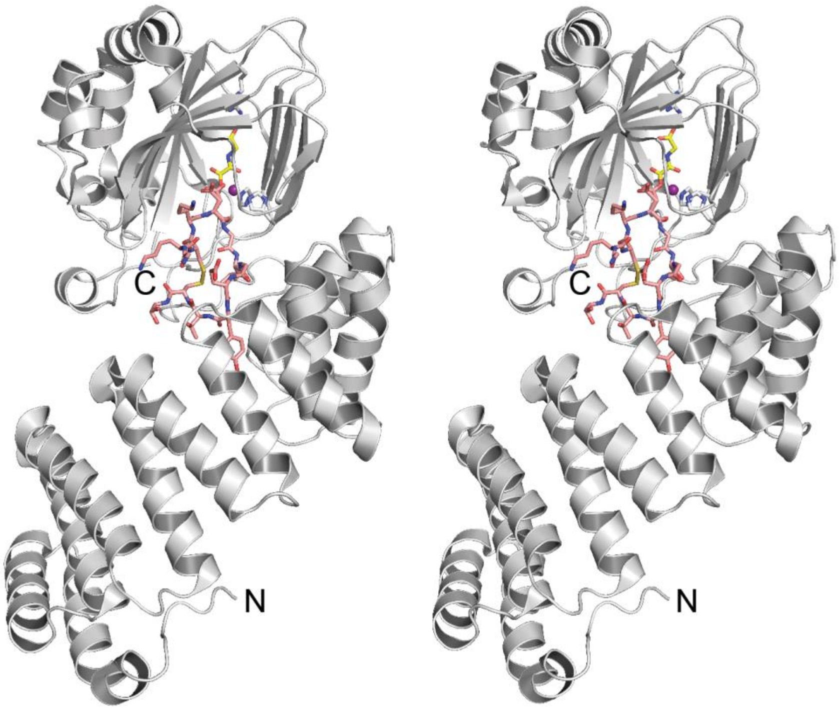

(c) Stereoview of the AspH-TPR-Ox:NC_{26mer} crystal structure active site: Mn(II) (violet sphere) coordinates two AspH-His residues (His679 and His725), a water molecule (red sphere), and NOG (yellow sticks) and is positioned to interact with the *pro-R* hydrogen of $Asp103_{hFX}$, the likely productive NC_{26mer} conformation (distance Cβ-Mn: 4.3 Å).

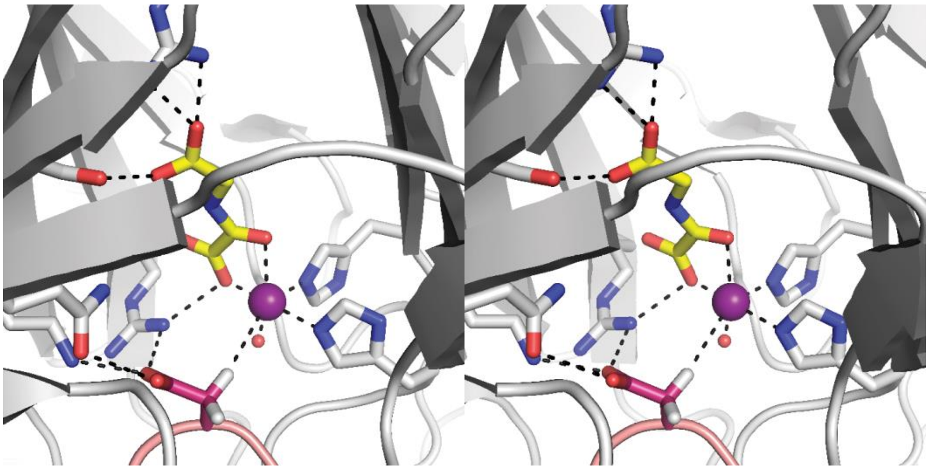

(d) OMIT electron density map ($mF_o{-}DF_c$) contoured to 3σ around the NC_{26mer} peptide from the AspH-TPR-Ox:NC_{26mer} crystal structure reveals electron density for two alternative conformations of $Asp103_{hFX}$. Furthermore, electron density is observed for $Tyr108_{hFX}$, which is part of the AspH-substrates consensus sequence requirements as it interacts with the TPR domain (shown above). A disulfide bridge between $Cys101_{hFX}$ and $Cys110_{hFX}$ forming a non-canonical macrocyclic peptide is clearly observed.

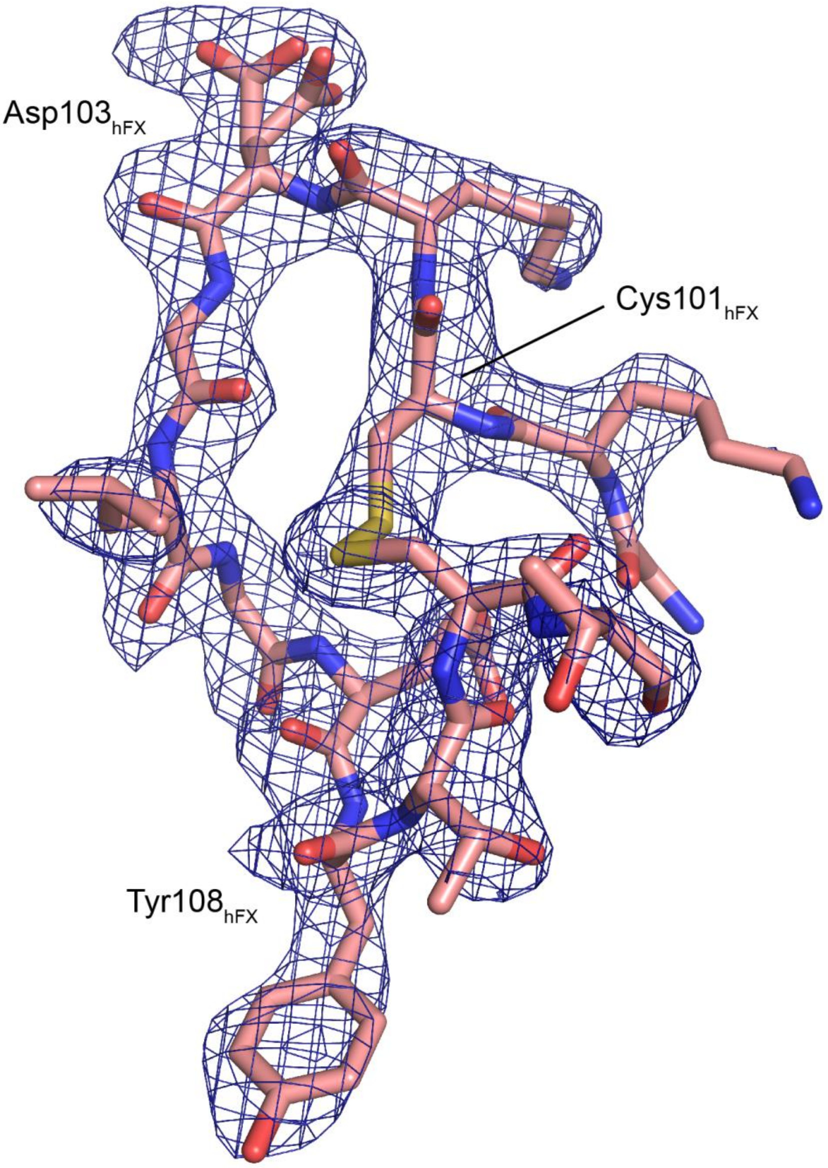

Supplementary Figure 18 (continues on the following 2 pages). Structure of AspH-TPR-Ox:NC-Ser$_{39mer}$ (PDB entry: 5JQY). Color code: grey: His$_6$-AspH$_{315-758}$; yellow: carbon-backbone of *N*-oxalylglycine (NOG); slate blue: carbon-backbone of the NC-Ser$_{39mer}$ peptide; violet: Mn(II); red: oxygen; blue: nitrogen; pale yellow: sulfur.

(a) His$_6$-AspH$_{315-758}$ binds the NC-Ser$_{39mer}$ peptide through interactions with active site residues, the hinge region, and the TPR domain: **(1)** Overview of the AspH-TPR-Ox:NC-Ser$_{39mer}$ structure; **(2)** Close-up of the active site (different view with respect to **1**): The acidic loop residue Asp616 interacts with the side chain of Lys102$_{hFX}$ (2.9 Å) and Glu617 with Lys102$_{hFX}$ (3.2 Å), Asp103$_{hFX}$ (2.8 Å), and Gly104$_{hFX}$ (2.8 Å) of the Ser$_{39mer}$ peptide. Furthermore, Gln627, Lys666, and Arg688 interact with Asp103$_{hFX}$ while Arg686 interacts with Lys102$_{hFX}$ (2.9 Å). Two alternative conformations of Asp103$_{hFX}$ are present in the crystal structure; **(3)** Additional interactions of the NC-Ser$_{39mer}$ peptide with His$_6$-AspH$_{315-758}$ are observed: His493 and Arg526 of the TPR domain interact with Tyr108$_{hFX}$ of the NC-Ser$_{39mer}$ peptide (3.0 respectively 2.8 Å) while Tyr565 interacts with Cys110$_{hFX}$ (2.9 Å); **(4)** Close-up of the interaction of Phe116$_{hFX}$ of the NC-Ser$_{39mer}$ peptide with Asn395 of the TPR domain (2.6 Å).

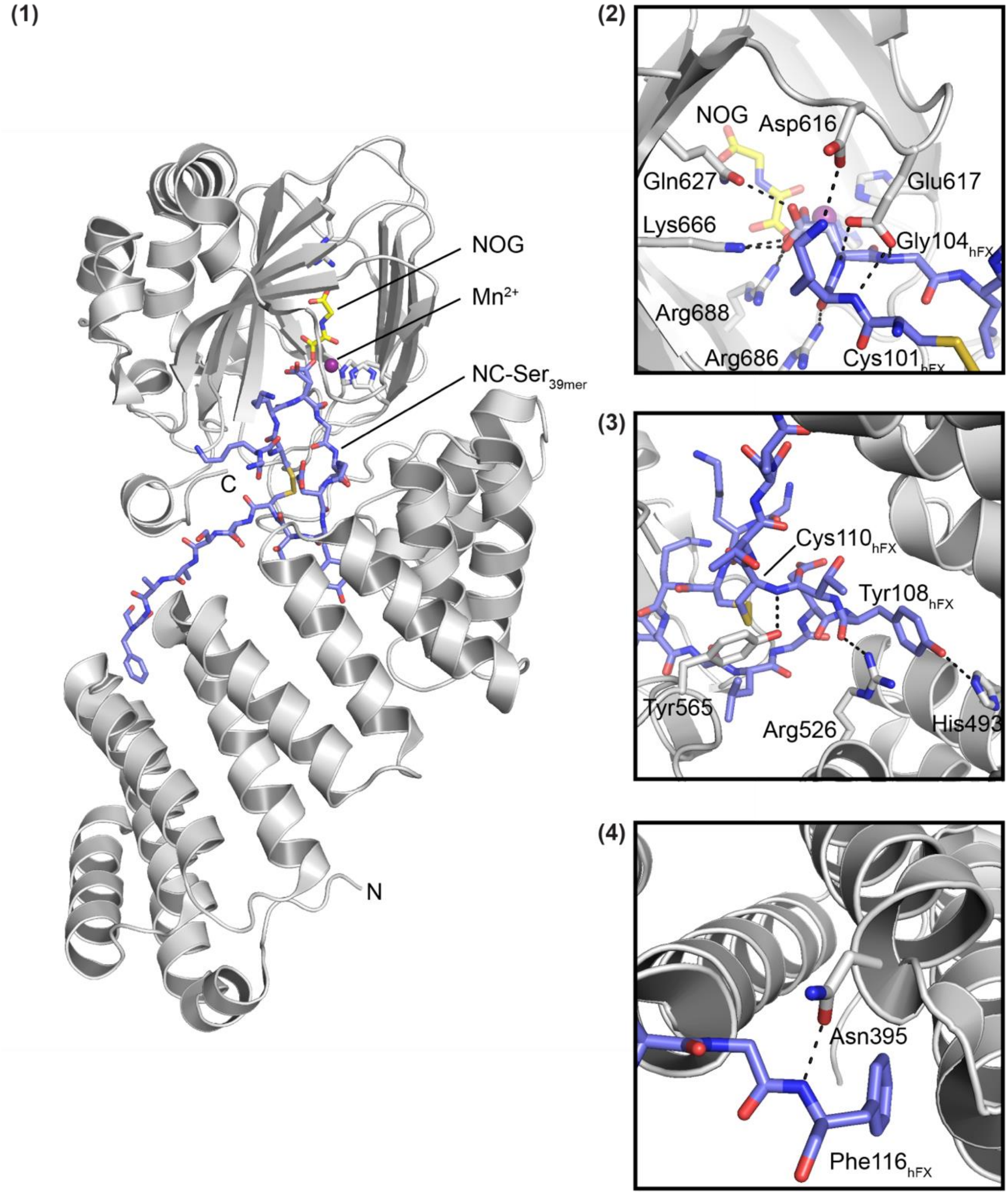

(b) Stereoview of the AspH-TPR-Ox:NC-Ser_{39mer} crystal structure.

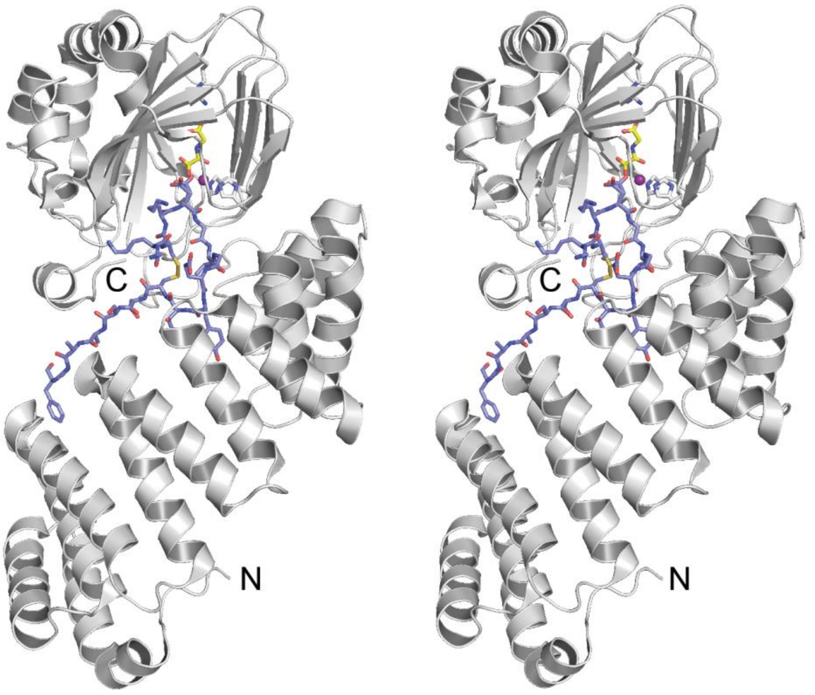

(c) Stereoview of the AspH-active site of the AspH-TPR-Ox:NC-Ser_{39mer} crystal structure: Mn(II) (violet sphere) coordinates two AspH-His residues (His679 and His725), a water molecule (red sphere), and NOG (yellow sticks) and is positioned to interact with the *pro-R* hydrogen of $Asp103_{hFX}$, the likely productive NC-Ser_{39mer} conformation (distance Cβ-Mn: 4.4 Å).

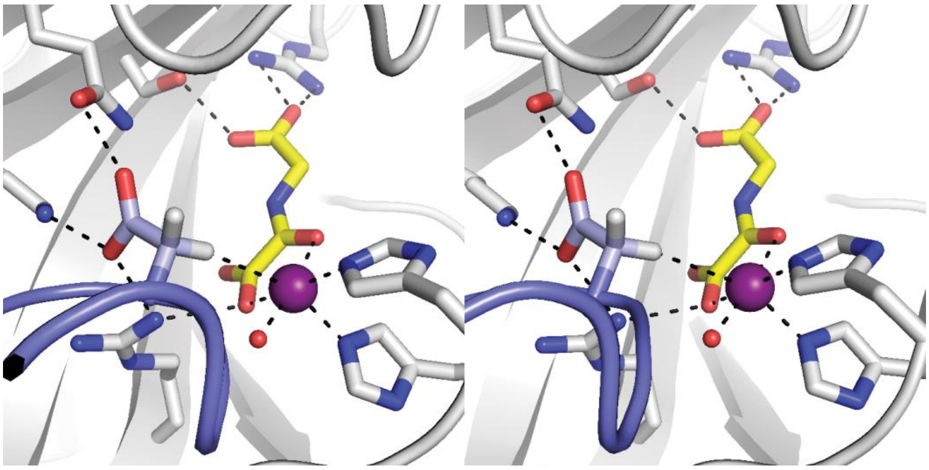

(d) OMIT electron density map ($mF_o{-}DF_c$) contoured to 3σ around the NC-Ser$_{39mer}$ peptide from the AspH-TPR-Ox:NC-Ser$_{39mer}$ crystal structure reveals electron density for two alternative conformations of Asp103$_{hFX}$. A macrocycle composed of 10 amino acids is formed by a disulfide bridge between Cys101$_{hFX}$ and Cys110$_{hFX}$. Furthermore, electron density for the N-terminal amino acids until Phe116$_{hFX}$ is, at least in part, observed.

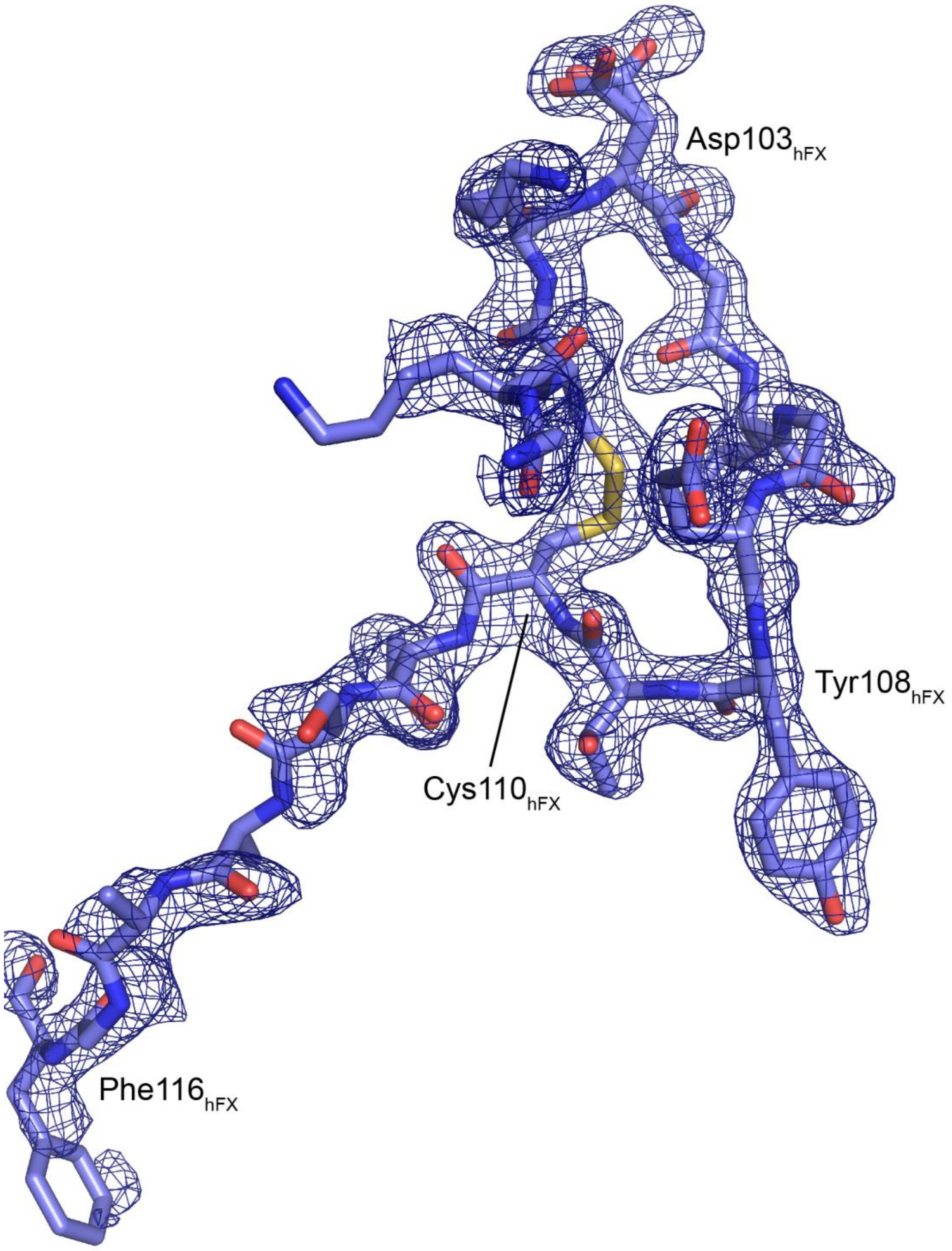

Supplementary Figure 19 (continues on the 2 following pages). Human multidomain EGFDs are Asn-β-hydroxylated by His$_6$-AspH$_{315-758}$; the hydroxylation is significantly enhanced under redox conditions. End-point turnover reactions were performed as outlined in the Methods Section with an incubation time of 180 min rather than 60 min; Prior to analysis, the substrate was reduced, and cysteine thiols were derivatized as thioethers by reaction with *N*-ethylmaleimide. Digestion using trypsin affording the predicted fragments indicated by fragmentation arrows as analysed using MALDI-ToF-MS. Light orange graphs represent controls in which AspH was replaced by buffer (no-enzyme control). Standard non redox buffer: 50 mM HEPES, pH = 7.5, 150 mM NaCl; Redox buffer: 50 mM Tris, pH = 8.5, 3.0 mM L-glutathione thiol, 0.3 mM L-glutathione disulphide, 150 mM NaCl.

(a) Asn1624$_{hFIB1}$-β-hydroxylation of human fibrillin-1, TB4cbEGF23 (hFIB1_TB4cbEGF23, aa 1527-1647; corresponding to TB6EGF27 in the Uniprot Database, entry: FBN1_HUMAN): **(1)** sequence and fragmentation pattern, the hydroxylation site (Asn1624$_{hFIB1}$) is in red; **(2)** <5% hydroxylation was observed under standard (non-redox) buffer conditions; **(3)** ~85% hydroxylation was observed under redox buffer conditions.

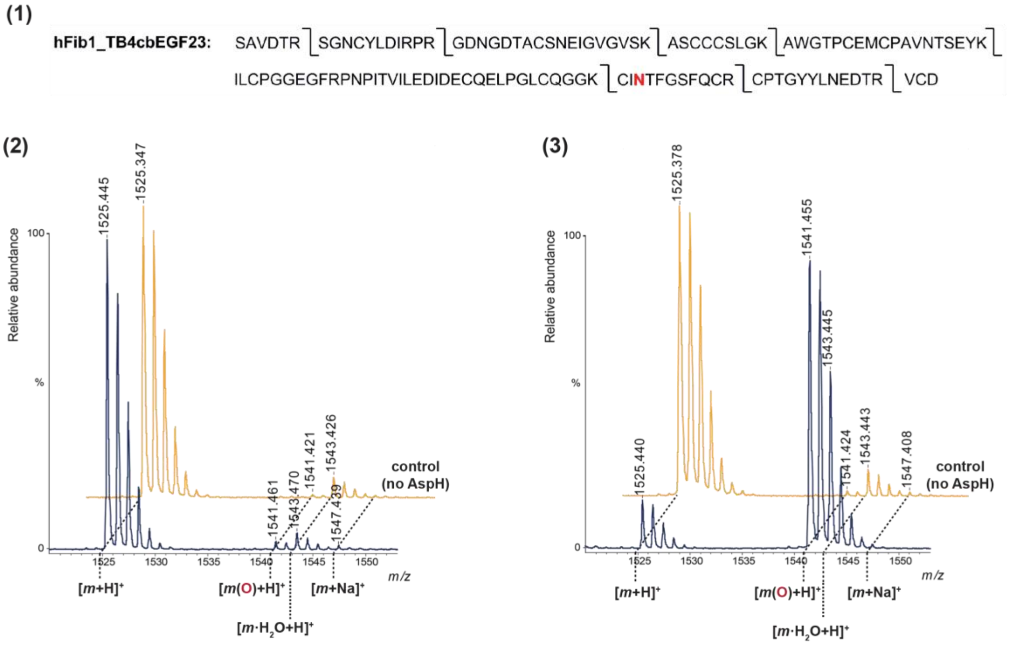

(b) Asn2144$_{hFIB1}$-β-hydroxylation of the N-terminal EGFD of human fibrillin-1, cbEGF32-33 (hFIB1_cbEGF32-33, aa 2124-2205; corresponding to EGF36-37 in the Uniprot Database, entry: FBN1_HUMAN): **(1)** sequence and fragmentation pattern, the hydroxylation site (Asn2144$_{hFIB1}$) is in red; **(2)** ~15% hydroxylation was observed under standard (non-redox) buffer conditions; **(3)** ~30% hydroxylation was observed under redox buffer conditions.

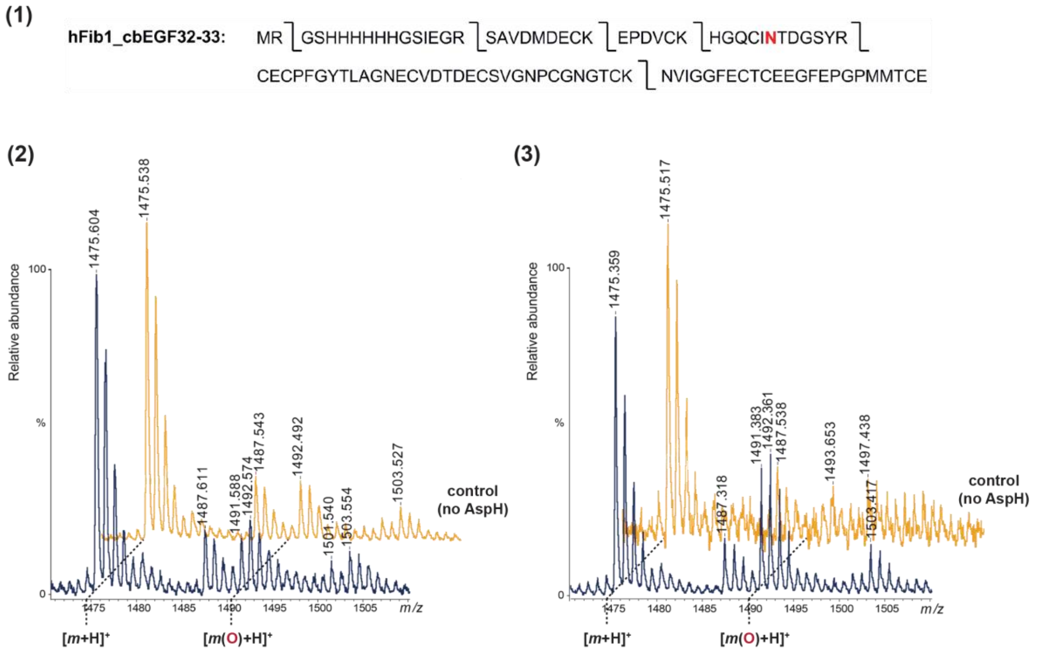

(c) Asn2583$_{hFIB1}$-β-hydroxylation of the N-terminal EGFD of human fibrillin-1, cbEGF41-43 (hFIB1_cbEGF41-43, aa 2567-2687; corresponding to EGF45-47 in the Uniprot Database, entry: FBN1_HUMAN): **(1)** sequence and fragmentation pattern, the hydroxylation site (Asn2583$_{hFIB1}$) is in red; **(2)** ~30% hydroxylation was observed under standard (non-redox) buffer conditions; **(3)** ~55% hydroxylation was observed under redox buffer conditions.

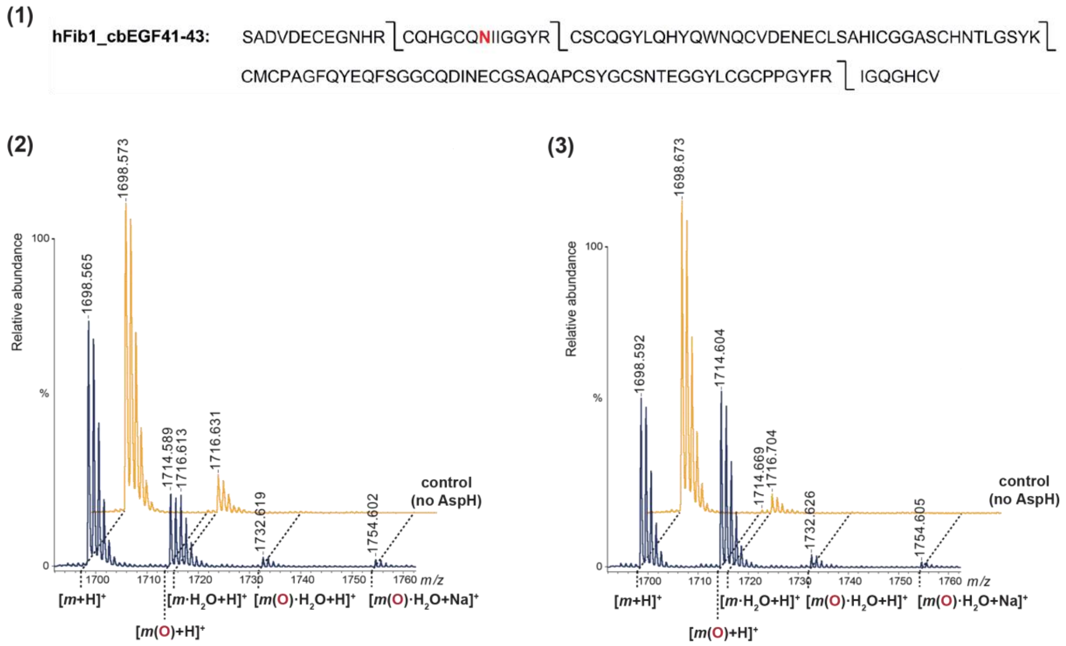

(d) Asn431$_{NOTCH1}$-β-hydroxylation of the N-terminal EGFD of human Notch-1, cbEGF11-13 (hNOTCH1_cbEGF11-13, aa 411-526): **(1)** sequence and fragmentation pattern, the hydroxylation site (Asn431$_{hNOTCH1}$) is in red; **(2)** no hydroxylation was observed under standard (non-redox) buffer conditions; **(3)** ~30% hydroxylation was observed under redox buffer conditions.

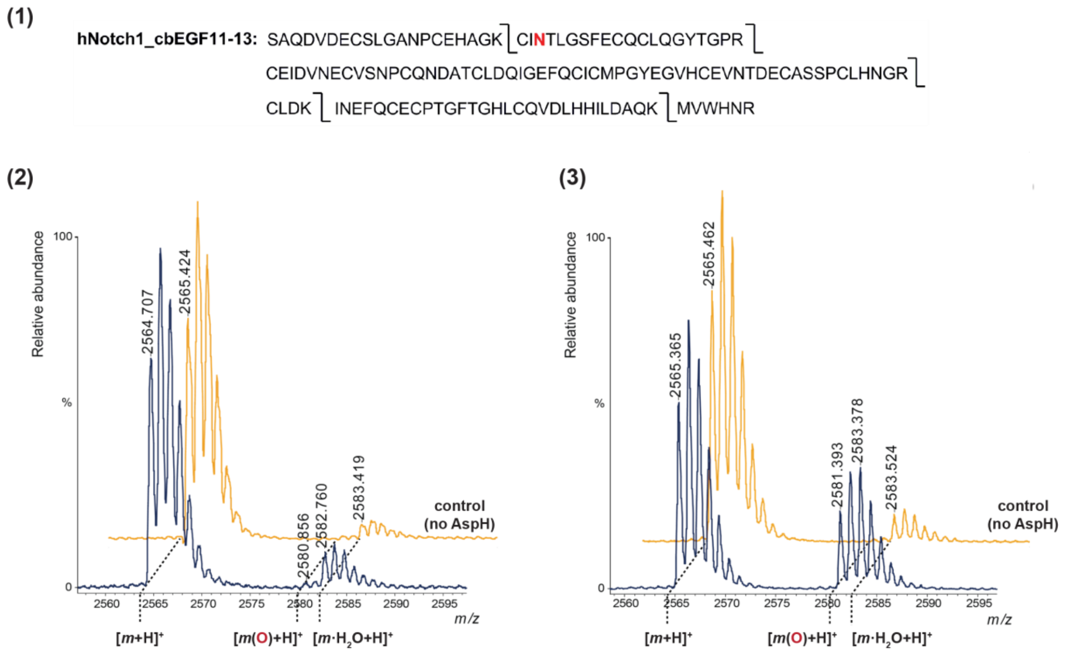

Supplementary Figure 20. Control experiments showing that Ca(II) ions inhibit the AspH-catalyzed Asn-β-hydroxylation of hFIB1_TB4cbEGF23 likely by binding to the EGFD-Ca(II)-binding site. End-point turnover reactions were performed as outlined in the Methods Section; hFIB1_TB4cbEGF23 was then reduced, and cysteine thiols were derivatized with *N*-ethylmaleimide. Digestion using trypsin was followed by analysis using MALDI-ToF-MS. Light orange graphs represent controls in which AspH was replaced by buffer (no-enzyme control)

(a) No effect of Mg(II) ions on the Asn1624$_{hFIB1}$-β-hydroxylation of hFIB1_TB4cbEGF23 was observed, out-ruling a chelation effect: **(1)** >95% hydroxylation under redox buffer conditions in the presence of 3 mM Mg(II).

(1)

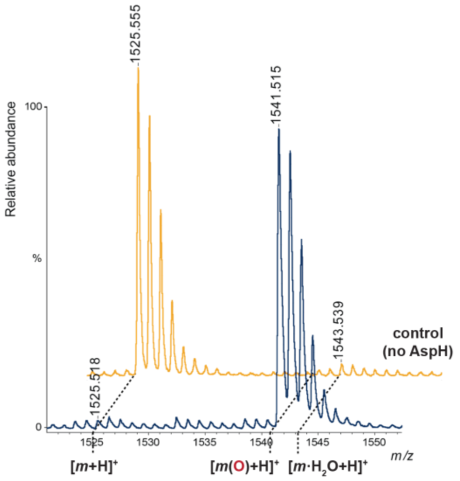

(b) No effect of Ca(II) ions on the Asp-β-hydroxylation of cyclic peptide CP2$_{101-110}$ was observed, further supporting the proposed effect on Ca(II) on the substrate rather than by binding to AspH since CP2$_{101-110}$ does not bind Ca(II): **(1)** Schematic structure of CP2$_{101-110}$ (the hydroxylation site, Asp103$_{hFX}$, is in red); **(2)** >95% hydroxylation under redox buffer conditions; **(3)** >95% hydroxylation was observed under redox buffer conditions in the presence of 10 mM Ca(II).

(1)

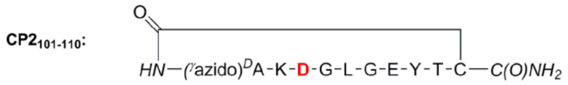

(2)

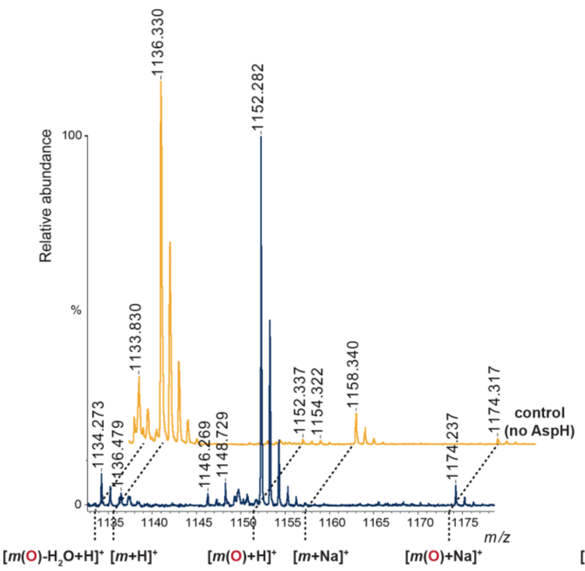

(3)

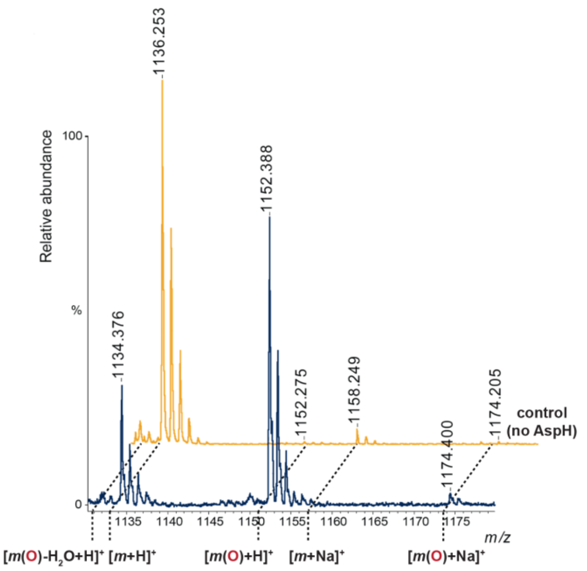

Supplementary Figure 21. Comparison of the active site of the AspH-TPR-Ox:NC-Ser$_{39mer}$ (PDB entry: 5JQY) crystal structure with those of FIH (PDB entry: 1H2K)[11] and AsnO (PDB entry: 2OG7)[12]. Color code: yellow: carbon-backbone of *N*-oxalylglycine (NOG); gold: succinate; violet: Mn(II); orange: Fe(II); red: oxygen; blue: nitrogen; pale yellow: sulfur. **(1)** Active site of AspH (grey: His$_6$-AspH$_{315-758}$; slate blue: carbon-backbone of the NC-Ser$_{39mer}$ peptide); **(2)** active site of FIH (green: FIH; cyan: carbon-backbone of the HIF-1α CAD$_{786-826}$ peptide); **(3)** active site of AsnO (pink: AsnO; limon: 3-hydroxyasparagine).
Comparison of the AspH, FIH, and AsnO active sites reveals the importance of arginine residues for binding substrate Asp/Asn-residues undergoing hydroxylation (**1**, AspH: Arg688 is positioned to interact with Asp103$_{hFX}$; **2**, FIH: Arg238$_{FIH}$ is positioned to interact with Asn803$_{HIF}$; **3**, AsnO: Arg305$_{AsnO}$ is positioned to interact with the C1-carboxylate of the 3-hydroxyasparagine product).

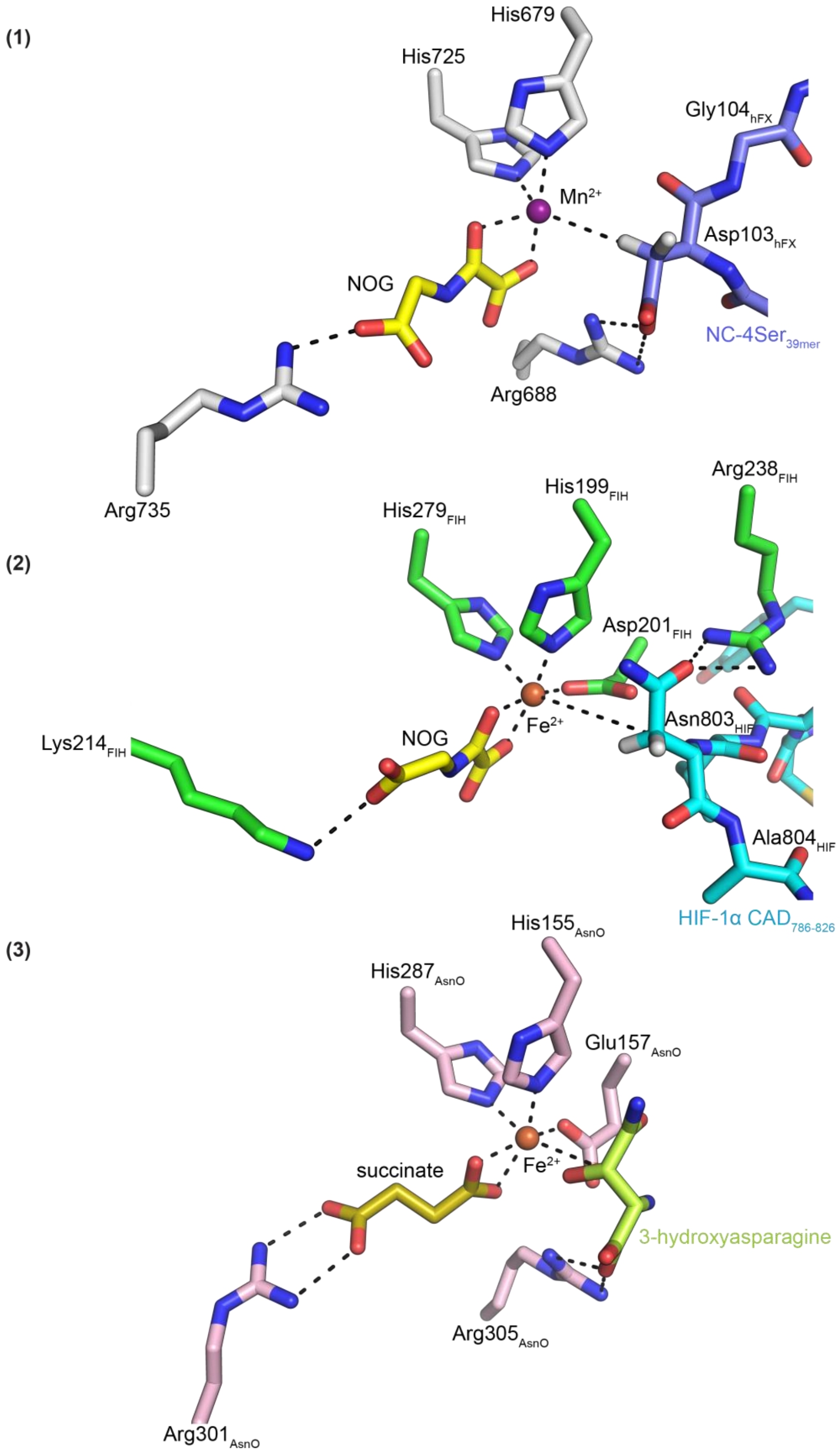

Supplementary Table 1: Crystallization conditions and data collection of His_6-AspH-complexes.

	$AspH_{562\text{-}758}{\cdot}Ni^{II}{\cdot}$ L-malate (AspH-Ox)	$AspH_{315\text{-}758}{\cdot}Mn^{II}{\cdot}$ NOG (“apo”) (AspH-TPR-Ox)	$AspH_{315\text{-}758}{\cdot}Mn^{II}{\cdot}$ L-malate (“apo”) (AspH-TPR-Ox:malate)	$AspH_{315\text{-}758}{\cdot}Mn^{II}{\cdot}$ NOG·hFX_{39mer} (AspH-TPR-Ox: hFX)	$AspH_{315\text{-}758}{\cdot}Mn^{II}{\cdot}$ NOG·NC-$4Ser_{39mer}$ (AspH-TPR-Ox: NC-$4Ser_{39mer}$)	$AspH_{315\text{-}758}{\cdot}Mn^{II}{\cdot}$ NOG·NC_{26mer} (AspH-TPR-Ox: NC_{26mer})	$AspH_{315\text{-}758}{\cdot}Mn^{II}{\cdot}$ NOG·$CP_{101\text{-}119}$ (AspH-TPR-Ox: $CP_{101\text{-}119}$)
**PDB ID**	5APA	5JZA	5JZ6	5JZ8	5JQY	5JZU	6RK9
**Crystallization**							
Method	Vapour diffusion, sitting drop (150 nL), protein-to-well ratio 1:2	Vapour diffusion, sitting drop (300 nL), protein-to-well ratio 1:2	Vapour diffusion, sitting drop (300 nL), protein-to-well ratio 1:2	Vapour diffusion, sitting drop (300 nL), protein-to-well ratio 1:2	Vapour diffusion, sitting drop (300 nL), protein-to-well ratio 2:1	Vapour diffusion, sitting drop (200 nL), protein-to-well ratio 1:1	Vapour diffusion, sitting drop (200 nL), protein-to-well ratio 1:1
Temperature (K)	293	277	277	277	277	277	277
Precipitation cond.	9.5 mg/mL His_6-$AspH_{562\text{-}758}$, 20 mM $NiSO_4$, 1 mM *N*-oxalylglycine, 50 mM sodium malate, $20\%_{w/v}$ PEG3350, 30% dextran sulfate	18 mg/mL His_6-$AspH_{315\text{-}758}$, 1 mM $MnCl_2$, 2 mM *N*-oxalylglycine, 100 mM bicine, pH 9.0, 100 mM sodium chloride, $27\%_{w/v}$ PEG550 MME	18 mg/mL His_6-$AspH_{315\text{-}758}$, 1 mM $MnCl_2$, 2 mM *N*-oxalylglycine, 1 mM MMT buffer (DL-malic acid, MES, tris base), pH 7.0, $25\%_{w/v}$ PEG1500	18 mg/mL His_6-$AspH_{315\text{-}758}$, 1 mM $MnCl_2$, 2 mM *N*-oxalylglycine, 100 mM Bis Tris Propane, pH 6.5, 200 mM sodium acetate, $20\%_{w/v}$ PEG3350, 726 µM hFX_$EGF1_{39mer}$	18 mg/mL His_6-$AspH_{315\text{-}758}$, 1 mM $MnCl_2$, 2 mM *N*-oxalylglycine, 100 mM SPG buffer, pH 8.0, $25\%_{w/v}$ PEG1500, 726 µM NC-$4Ser_{39mer}$	18 mg/mL His_6-$AspH_{315\text{-}758}$, 1 mM $MnCl_2$, 2 mM *N*-oxalylglycine, 100 mM Bis Tris Propane, pH 6.5, 20 mM sodium/potassiu m phosphate, $20\%_{w/v}$ PEG3350, 1.65 mM NC_{26mer}	18 mg/mL His_6-$AspH_{315\text{-}758}$, 1 mM $MnCl_2$, 2 mM *N*-oxalylglycine, 100 mM Bis Tris Propane, pH 6.5, 200 mM potassium thiocyanate, $20\%_{w/v}$ PEG3350, 3.3 mM $CP2_{19mer}$
AspH:substrate ratio	-	-	-	1:2.2 (330:726 µM)	1:2.2 (330:726 µM)	1:5 (330:1650 µM)	1:10 (330:3300 µM)
**Data collection**							
Space group	$P3_121$	$P2_12_12_1$	$P2_12_12_1$	$P2_12_12_1$	$P2_12_12_1$	$P2_12_12_1$	$P1$
Symmetry	trigonal	orthorhombic	orthorhombic	orthorhombic	orthorhombic	orthorhombic	triclinic
Cell dimensions:							
a, *b*, *c* (Å)	133.22, 133.22, 44.64	48.73, 70.24, 170.42	49.02, 70.64, 172.54	50.25, 91.02, 122.67	50.17, 89.54, 123.55	50.23, 90.81, 124.09	49.47, 59.24, 95.67
α, β, γ (°)	90.0, 90.0, 120.0	90.00, 90.00, 90.00	90.00, 90.00, 90.00	90.00, 90.00, 90.00	90.00, 90.00, 90.00	90.00, 90.00, 90.00	103.97, 91.49, 92.70
X-Ray source[a)]	Synchrotron (DLS I04)	Synchrotron (DLS I02)	Synchrotron (DLS I04)	Synchrotron (DLS I04)	Synchrotron (DLS I04)	Synchrotron (DLS I04)	Copper rotating anode (CuKα)
Temperature (K)	100	100	100	100	100	100	100
Detector	ADSC CCD	Pilatus 6M	Pilatus 6M-F	Pilatus 6M-F	Pilatus 6M-F	Pilatus 6M-F	Saturn 944+ CCD
Resolution (Å)	19.87-2.05 (2.12-2.05)[b)]	9.57-2.14 (2.20-2.14)	47.15-2.35 (2.43-2.35)	29.56-2.09 (2.18-2.10)	46.48-1.99 (2.04-1.99)	11.18-2.50 (2.56-2.50)	32.00-2.30 (2.38-2.30)
R_{merge}	0.09 (0.82)	0.049 (0.652)	0.14 (0.896)	0.083 (0.639)	0.061 (0.654)	0.134 (0.870)	0.069 (0.466)
$I / \sigma I$	11.5 (2.3)	18.6 (3.1)	14.2 (2.5)	12.8 (2.8)	28.8 (4.5)	14.6 (3.2)	10.4 (1.7)
Completeness (%)	99.9 (100)	99.9 (99.9)	99.8 (99.9)	99.4 (99.2)	100.0 (100.0)	99.9 (100.0)	99.9 (100.0)
Multiplicity	7.4 (7.5)	6.4 (6.8)	6.5 (5.9)	6.0 (5.6)	13.1 (13.5)	12.9 (13.8)	5.6 (5.5)

[a)]DLS: Diamond Light Source; [b)]Values in parentheses are for highest-resolution shell.

Supplementary Table 2: Refinement statistics of His_6-AspH-complexes.

	$AspH_{562-758}\cdot Ni^{II}\cdot$ L-malate (AspH-Ox)	$AspH_{315-758}\cdot Mn^{II}\cdot$ NOG ("apo") (AspH-TPR-Ox)	$AspH_{315-758}\cdot Mn^{II}\cdot$ L-malate ("apo") (AspH-TPR-Ox:malate)	$AspH_{315-758}\cdot Mn^{II}\cdot$ NOG·hFX_{39mer} (AspH-TPR-Ox: hFX)	$AspH_{315-758}\cdot Mn^{II}\cdot$ NOG·NC-$4Ser_{39mer}$ (AspH-TPR-Ox: NC-$4Ser_{39mer}$)	$AspH_{315-758}\cdot Mn^{II}\cdot$ NOG·NC_{26mer} (AspH-TPR-Ox: NC_{26mer})	$AspH_{315-758}\cdot Mn^{II}\cdot$ NOG·$CP_{101-119}$ (AspH-TPR-Ox: $CP_{101-119}$)
**PDB ID**	5APA	5JZA	5JZ6	5JZ8	5JQY	5JZU	6RK9
**Refinement**							
Resolution (Å)	2.05	2.14	2.35	2.10	1.99	2.50	2.30
No. reflections	28718	33109	25573	33608	38985	20301	46871
R_{work} / R_{free}	0.1529/ 0.1855	0.1751/ 0.1976	0.1944/ 0.2214	0.1881/ 0.2162	0.1804/ 0.1959	0.1889/ 0.2213	0.2107/ 0.2350
No. atoms:							
Protein	1616	3462	3453	3580	3580	3580	6966
Ligand/ion	18	35	34	15	15	15	27
Water	139	125	202	305	283	105	230
B-factors:							
Protein	46.8	68.8	50.4	41.9	43.7	45.8	54.6
Ligand/ion	44.3	77.1	40.2	32.2	38.3	42.2	42.8
Water	52.1	56.8	65.1	42.0	44.5	53.5	45.4
R.m.s. deviations:							
Bond lengths (Å)	0.007	0.003	0.003	0.003	0.004	0.004	0.003
Bond angles (°)	0.994	0.706	0.723	0.700	0.829	0.797	0.515

Supplementary References

1. Geer, L. Y. *et al.* The NCBI BioSystems database. *Nucleic Acids Res.* **38**, D492-D496 (2010).
2. Nicholas, K. B., Nicholas Jr., H. B. & Deerfield II., D. W. GeneDoc - Analysis and visualization of genetic variation. *embnet.news* **4**, 1-4 (1997).
3. Geoghegan, K. F. *et al.* Spontaneous α-N-6-Phosphogluconoylation of a "His Tag" in Escherichia coli: The Cause of Extra Mass of 258 or 178 Da in Fusion Proteins. *Anal. Chem.* **267**, 169-184 (1999).
4. Yan, Z., Caldwell, G. W. & McDonell, P. A. Identification of a gluconic acid derivative attached to the N-terminus of histidine-tagged proteins expressed in bacteria. *Biochem. Biophys. Res. Commun.* **262**, 793-800 (1999).
5. Passmann, J. M., Radin, N. S. & Cooper, J. A. D. Liquid scintillation technique for measuring carbon-14-dioxide activity. *Anal. Chem.* **28**, 484-486 (1956).
6. Gouet, P. & Robert, X. Deciphering key features in protein structures with the new ENDscript server. *Nucleic Acids Res.* **42**, W320-W324 (2014).
7. Bean, M. F. & Carr, S. A. Characterization of disulfide bond position in proteins and sequence analysis of cystine-bridged peptides by tandem mass spectrometry. *Anal. Chem.* **201**, 216-226 (1992).
8. Gorman, J. J., Wallis, T. P. & Pitt, J. J. Protein disulfide bond determination by mass spectrometry. *Mass Spectrom. Rev.* **21**, 183-216 (2002).
9. Olsen, J. V., Ong, S.-E. & Mann, M. Trypsin cleaves exclusively C-terminal to arginine and lysine residues. *Mol. Cell. Proteom.* **3**, 608-614 (2004).
10. Bonnici, J., Tumber, A., Kawamura, A. & Schofield, C. J. Inhibitors of both the N-methyl lysyl- and arginyl-demethylase activities of the JmjC oxygenases. *Phil. Trans. R. Soc. B* **373**, 20170071 (2018).
11. Elkins, J. M. *et al.* Structure of factor-inhibiting hypoxia-inducible Factor (HIF) reveals mechanism of oxidative modification of HIF-1α. *J. Biol. Chem.* **278**, 1802-1806 (2003).
12. Strieker, M., Kopp, F., Mahlert, C., Essen, L.-O. & Marahiel, M. A. Mechanistic and structural basis of stereospecific Cβ-hydroxylation in calcium-dependent antibiotic, a daptomycin-type lipopeptide. *ACS Chem. Biol.* **2**, 187-196 (2007).
